# MS-275, a class 1 histone deacetylase inhibitor augments glucagon-like peptide-1 receptor agonism to improve glycemic control and reduce obesity in diet-induced obese mice

**DOI:** 10.7554/eLife.52212

**Published:** 2020-12-22

**Authors:** Shilpak Bele, Shravan Babu Girada, Aramita Ray, Abhishek Gupta, Srinivas Oruganti, Phanithi Prakash Babu, Rahul SR Rayalla, Shashi Vardhan Kalivendi, Ahamed Ibrahim, Vishwajeet Puri, Venkateswar Adalla, Madhumohan R Katika, Richard DiMarchi, Prasenjit Mitra

**Affiliations:** 1 Dr. Reddy’s Institute of Life Sciences University of Hyderabad CampusHyderabadIndia; 2 Manipal Academy of Higher EducationManipalIndia; 3 Department of Biomedical Sciences and Diabetes Institute, Ohio UniversityAthensUnited States; 4 School of Life Sciences, University of HyderabadHyderabadIndia; 5 Department of Applied Biology, Indian Institute of Chemical TechnologyHyderabadIndia; 6 Division of Lipid Chemistry, National Institute of Nutrition HyderabadHyderabadIndia; 7 Medical Genomics, QIMR Berghofer Medical Research InstituteHerstonAustralia; 8 Stem Cell and Regenerative Medicine Department, Nizam’s Institute of Medical SciencesHyderabadIndia; 9 Department of Chemistry, Indiana UniversityBloomingtonUnited States; University of California, San FranciscoUnited States; Utrecht UniversityNetherlands

**Keywords:** glucagon-like peptide-1 receptor, HDAC inhibition, insulin secretion, obesity, energy expenditure, glycemic control, Mouse

## Abstract

Given its glycemic efficacy and ability to reduce the body weight, glucagon-like peptide 1 receptor (GLP-1R) agonism has emerged as a preferred treatment for diabetes associated with obesity. We here report that a small-molecule Class 1 histone deacetylase (HDAC) inhibitor Entinostat (MS-275) enhances GLP-1R agonism to potentiate glucose-stimulated insulin secretion and decrease body weight in diet-induced obese (DIO) mice. MS-275 is not an agonist or allosteric activator of GLP-1R but enhances the sustained receptor-mediated signaling through the modulation of the expression of proteins involved in the signaling pathway. MS-275 and liraglutide combined therapy improved fasting glycemia upon short-term treatment and a chronic administration causes a reduction of obesity in DIO mice. Overall, our results emphasize the therapeutic potential of MS-275 as an adjunct to GLP-1R therapy in the treatment of diabetes and obesity.

## Introduction

Type 2 diabetes (T2D) and obesity have reached global epidemic levels and required a therapeutic intervention to reduce the burden of the disease. Incretin-based therapy and specifically glucagon-like peptide 1 receptor (GLP-1R) agonists provide sizable glycemic benefit and modest improvement in the body weight (Astrup et al., 2009). Unfortunately, not all patients achieve normal glucose control, and even fewer show reversal of obesity (Amori et al., 2007). The unmet medical need warrants additional complementary mechanisms to incretin action (Tschöp and DiMarchi, 2017) or a novel approach to supplement incretin pharmacology.

Incretin receptors can be activated by orthosteric peptide-based agonists (Drucker et al., 2010), dual agonists (Finan et al., 2013), and small-molecule allosteric modulators (Knudsen et al., 2007; Bueno et al., 2016). In each instance, the receptor stabilizes in an active conformation suitable for the association with heterotrimeric G-protein subunit Gα_s_ and subsequent activation of the adenylate cyclase. The activation of the receptor propels a cellular signaling cascade that eventually potentiates glucose-stimulated insulin secretion (GSIS) (Drucker, 2006). The canonical pathway of GPCR activation postulates increases in the second messenger cAMP following the receptor activation that rapidly attenuates with the receptor internalization and desensitization. More recent reports, however, demonstrate sustained cAMP generation for several GPCRs following internalization and the formation of a multi-protein complex at endosomes where activated receptor-ligand complex, the Gα_s_ subunit of the heterotrimeric G-protein and beta arrestin-1 contribute as key components (Thomsen et al., 2016). Previous observations from our laboratory have shown that prolonged association of the Gα_s_ subunit with the activated and internalized GLP-1R at Rab5 endosomes sustains cAMP generation to support GSIS in pancreatic beta cells (Girada et al., 2017). These results led to our hypothesis to assess whether the increase in the expression of the auxiliary proteins that supports GPCR-mediated sustained cAMP generation could enhance GLP-1R function. If so, the metabolic and body weight benefits of GLP-1 therapy would be sizably enhanced.

We report herein the identification of a small molecule inhibitor of Class 1 histone deacetylases (HDACs) that significantly enhances the GLP-1R signaling. From a relatively smaller compound library, four Class 1 HDAC inhibitors were confirmed to enhance GLP-1R-mediated signaling, the most prominent among them being the Class 1 HDAC-inhibitor named Entinostat (MS-275) (Suzuki et al., 1999; Saito et al., 1999). Our findings show that MS-275 enhances the expression of the genes involved in the GLP-1R signaling cascade improving fasting glycemia upon a short-term treatment and a chronic combined therapy reduces obesity in the DIO rodent model. The data, taken together thus provides a new dimension to the treatment of T2D and obesity.

## Results

### Screening for augmentation of incretin signaling in pancreatic beta cells

**Figure 1.**
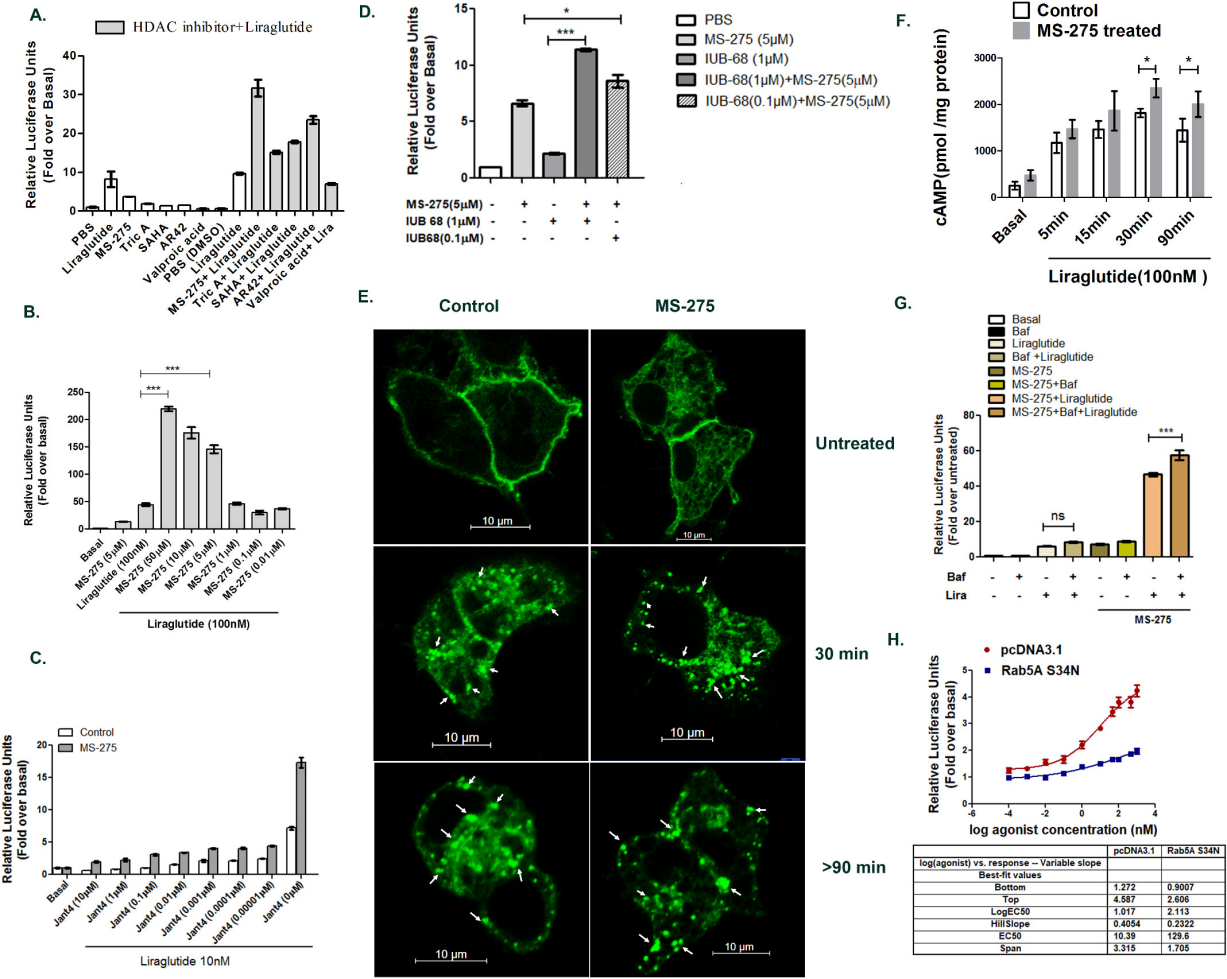
Class 1 HDAC inhibitor MS-275 promotes incretin receptor signaling. (**A**) Primary screening using BRIN-BD11 pancreatic beta cells to identify activators of GLP-1R-mediated cAMP generation as assessed by a luciferase reporter assay. Y-axis represents relative Luciferase units normalized by β-galactosidase expression. Liraglutide (100 nM) enhanced cAMP generation over basal, untreated control by 8.20 ± 2.84 fold which was further increased to 17 ± 0.4 fold, 15.2 ± 0.6 fold, 23.5 ± 1.4 fold, and 31.7 ± 3.1 fold in presence of SAHA, TSA, AR-42, and MS-275 respectively (n = 2 replicates per treatment). The concentration of each compound used in primary screening is 10 μM. The final DMSO concentration is 0.01% (**B**) Generation of cAMP in BRIN-BD11 pancreatic beta cells pretreated for 18 h with MS-275 at different concentrations. Liraglutide at 100 nM provides GLP-1R agonism and results represent mean (± SE) for three independent experiments, each treatment being conducted in duplicate. ***p<0.001 was determined by analysis of variance (ANOVA) using Tukey’s multiple comparison test comparing different concentrations of MS-275 upon liraglutide-induced cAMP generation as shown in normalized relative luciferase units as fold-over basal. (**C**) Effect of Jant 4 on GLP-1R-mediated cAMP generation in control and MS-275-treated BRIN-BD11 pancreatic beta cells. Results were reported as fold-increase relative to basal (untreated control). Data represented as mean ± SD (n = 2 replicates per treatment). (**D**) GIPR-mediated cAMP generation in cultured pancreatic beta cells pretreated for 18 h with MS-275(5 μM). IUB68 at different concentrations provides GIPR agonism. Data represent mean (± SE) of three independent experiments, each treatment being conducted in replicate. ***p<0.001 was determined by analysis of variance (ANOVA) using Tukey’s multiple comparison test comparing the effect of MS-275 upon IUB68 treatment at different concentrations. The generation of cAMP is measured in relative luciferase units and is represented as a fold-over basal cAMP generation. (**E**) The GLP-1R GFP trafficking in control and MS-275-treated pancreatic beta cells upon activation by liraglutide. BRIN-BD11 pancreatic beta cells were transfected with GFP-tagged GLP-1R and stimulated with 100 nM liraglutide for different time intervals. They were then fixed and visualized by confocal microscopy. White arrows pointing at the punctate dots represented internalized activated GLP-1R. Images were representative of three independent experiments, n = 25 cells for each time point. (**F**) The time course of GLP-1R-mediated cAMP generation in cultured pancreatic beta cells pretreated for 18 h with MS-275 (5 μM). The control and MS-275-treated cells were incubated with liraglutide (100 nM) and 5 min after the incubation the excess ligand was washed with KRB buffer. The cAMP was measured 5, 15, 30, and 90 min after KRB wash using Direct cAMP Enzyme Immunoassay. The statistically significant increase in the cAMP generation on liraglutide agonism between the control and MS-275-treated cells was assessed at 30 and 90 min time points (p<0.05, two-way ANOVA, Bonferroni’s post-tests). Results represent the mean (± SE) of three independent experiments. (**G**) Effect of Bafilomycin A1 (100 nM) on MS-275-mediated induction of GLP-1R-mediated cAMP generation. Results represent mean (± SE) of three independent experiments and are presented as fold-over basal (untreated control); ***p<0.001 determined by analysis of variance (one-way ANOVA, Tukey’s multiple comparison test) comparing the effect of Bafilomycin A1 in control and MS-275-treated cells on liraglutide-stimulated cAMP generation. (**H**) Effect of Rab5A S34N dominant negative plasmid on the MS-275-mediated augmentation of GLP-1R signaling measured by cAMP generation using luciferase assay. BRIN-BD11 pancreatic beta cells were transfected with Rab5A S34N dominant-negative plasmid and 12 h post-transfection was treated with MS-275. After 24 h of treatment cAMP assay was performed. The data was presented as a four-parameter-logistic curve analyzed in Prism (version 6.0), and each data point was assessed in duplicates. The dose-response curve represents the mean ± SEM of three independent experiments. Figure 1—source data 1.Source Data 1A: Primary screening data of small molecules for stimulation of GLP-1R-mediated cAMP generation.Source Data 1B: MS-275 dose-response for induction of GLP-1R-mediated cAMP generation in cultured pancreatic beta cells. Source Data 1C: Impact of GLP-1R antagonist Jant4 on liraglutide-mediated cAMP generation in control and MS-275-treated cultured pancreatic beta cells. Source Data 1D: MS-275-mediated potentiation of GIPR agonist IUB68-mediated cAMP generation in BRIN-BD11 pancreatic beta cells. Source Data 1F: Time course of GLP-1R-mediated cAMP generation in control and MS-275-treated pancreatic beta cells. Source Data 1G: Impact of Bafilomycin A1 on MS-275-induced stimulation of GLP-1R-mediated cAMP generation. Source Data 1H: Effect of Rab5A S34N dominant-negative plasmid on the MS-275-mediated augmentation of GLP-1R signaling.

We screened an unbiased compound library comprising of 150 small molecules to search for the enhancement of the activity of GLP-1R agonist, liraglutide. The screening paradigm involved incubation of BRIN–BD11 pancreatic beta cells with each small molecule for 18h before being stimulated with liraglutide for the cAMP response. Four compounds enhanced the GLP-1R-mediated cAMP generation (Figure 1A, Supplementary file 1-Table 1). Interestingly, each of these compounds is an HDAC inhibitor possessing either a hydroxamate or an orthoanilide group. These compounds are not direct GLP-1R agonists as assessed by their independent inability to promote cAMP generation acutely in BRIN-BD11 pancreatic beta cells (Figure 1—figure supplement 1). Our data showed that Suberoylanilide Hydroxamic Acid (SAHA), Trichostatin A, AR-42, and MS-275 enhanced GLP-1R agonism of liraglutide in cultured pancreatic beta cells. The most effective molecule in our screening was MS-275 (also known as entinostat), a Class 1 HDAC inhibitor that inhibits HDAC1 and HDAC3 with comparable potency (Lauffer et al., 2013). As our data showed, MS-275 significantly enhanced liraglutide-mediated GLP-1R agonism as measured by the enhancement of receptor-mediated cAMP response. Treatment of BRIN-BD11 pancreatic beta cells with increasing concentrations of MS-275 demonstrated a dose-dependent enhancement of liraglutide-mediated cAMP generation (Figure 1B) revealing an EC50 of 4.09 μM (Figure 1—figure supplement 2). Overall, we observed a 3.464 ± 0.244 fold enhancement of liraglutide-mediated cAMP generation upon MS-275 treatment. The GLP-1R antagonist Jant 4 repressed this GLP-1R-mediated response highlighting the specificity of the induction (Figure 1C). In a similar fashion to GLP-1R, MS-275 enhanced the agonism of IUB68, a specific glucose-dependent insulinotropic peptide receptor (GIPR) ligand in BRIN-BD11 pancreatic beta cells (Figure 1D). The combination of the MS-275 with IUB68 was superior to either agent when tested separately. Collectively, these results indicated that pancreatic beta cells were more responsive to incretin stimulation when cultured in the presence of MS-275 and indicated the upregulation of the intracellular molecular signaling that promoted basal and incretin-mediated cAMP generation.

**Figure 1—figure supplement 1.**
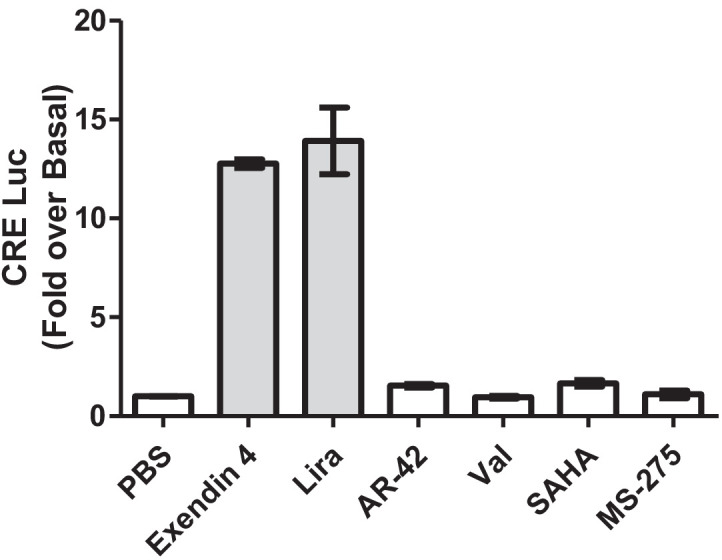
Direct GLP-1R agonism assessment of primary hits obtained from the cell-based screening of GLP-1R-mediated cAMP generation as measured by fold-increase over vehicle control in cAMP-responsive luciferase (Cre) reporter assay in BRIN-BD11 pancreatic beta cells.

**Figure 1—figure supplement 2.**
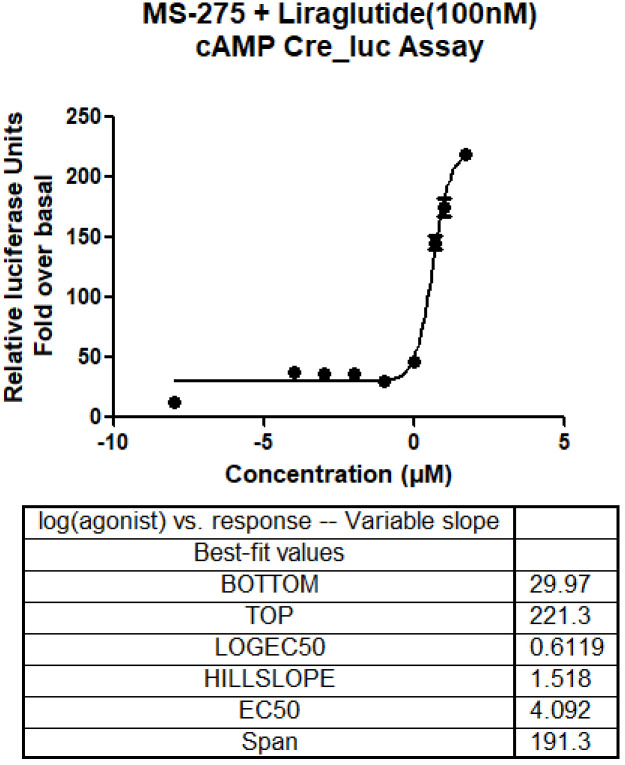
Dose-response curve depicting the role of MS-275 on GLP-1R-mediated cAMP generation.

**Figure 1—figure supplement 3.**
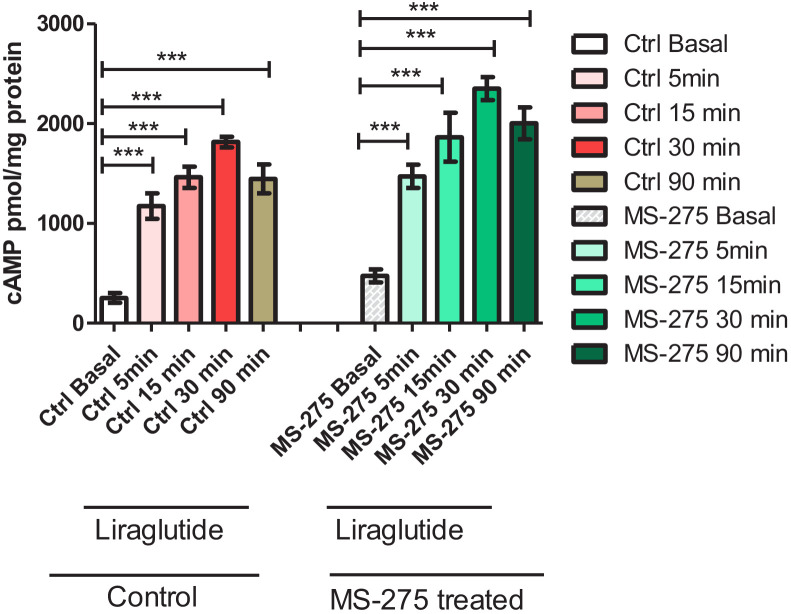
Liraglutide-mediated cAMP generation as compared to basal in control and MS-275-treated pancreatic beta cells. Test for statistical significance between basal and upon liraglutide treatment is carried out using one-way ANOVA (Tukey’s multiple comparison test).

### MS-275 promotes prolonged GLP-1R signaling

The canonical pathway of class B-GPCR activation involved rapid desensitization following the ligand binding and receptor internalization; however, recent findings support sustained signaling of the activated GPCR–ligand complex from endosomes (Calebiro et al., 2009; Ferrandon et al., 2009; Irannejad and von Zastrow, 2014; Ismail et al., 2016; Kuna et al., 2013).GLP-1R trafficking and the sustained cAMP generation from Rab5 endosomes upon activation by exendin-4 or GLP-1 tetra methyl-rhodamine (GLP-1Tmr) had previously been reported from our laboratory (Girada et al., 2017; Kuna et al., 2013). Our present study of GLP-1R trafficking following the activation with liraglutide revealed distinct receptor-trafficking kinetics as we observed a substantial localization of the activated receptor as cytoplasmic dots even after 90 min post-internalization (Figure 1E). Consequently, we evaluated the time course of cAMP generation upon liraglutide treatment in the presence and absence of MS-275. Pancreatic beta cells were treated with liraglutide, and the excess ligand was washed away with Krebs Ringer Buffer (KRB) 5 min following the treatment. The cAMP generation was determined 5, 15, 30, and 90 min after KRB wash by direct immunoassay (Baggio et al., 2004; Girada et al., 2017). The data presented in this study reveals that liraglutide causes a significant increase in cAMP generation both in control and MS-275-treated pancreatic beta cells (Figure 1—figure supplement 3). As Figure 1F shows, MS-275 treatment enhanced cAMP generation at 30 min and 90 min following liraglutide treatment (p<0.05 two-way ANOVA, Bonferroni post-tests) over control cells when the activated receptor had achieved substantial residence in the cytoplasm as punctate dots (Figure 1E, *white arrows*). The data thus highlighted the efficiency of the Class 1 HDAC inhibitor in increasing the GLP-1R-mediated cAMP generation upon internalization of the activated receptor. To validate our observation, we treated the cells with Bafilomycin A1, a specific inhibitor of v-ATPase that prevents late-stage vesicle maturation by suppressing endosomal acidification (Bayer et al., 1998). In control cells, we observed an increase in GLP-1R-mediated cAMP generation over basal values upon Bafilomycin A1 treatment. However, in MS-275-treated pancreatic beta cells, Bafilomycin inhibition further enhanced GLP-1R-mediated cAMP generation from 46.68 ± 0.95 fold to 57.7 ± 2.84 fold (p<0.001, n = 3; one way ANOVA Tukey’s multiple comparison test), relative to the basal cAMP generation (Figure 1G). The data thus indicated that the inhibition of endosomal maturation enhanced MS-275-mediated GLP-1R response. In contrast, the inhibition of GLP-1R internalization upon expression of Rab5A S34N dominant-negative plasmid (Girada et al., 2017) significantly reduced the MS-275-mediated augmentation of GLP-1R signaling (EC_50_129.6 nM in Rab5A S34N transfected cells as compared to EC_50_10.39 nM) upon control plasmid transfection (Figure 1H). These results, taken together, indicated the efficacy of MS-275 to augment the sustained cAMP generation post-activation and internalization of the receptor thereby promoting prolonged GLP-1R action in the pancreatic beta cells.

### MS-275 alters transcriptome profile in BRIN-BD11 pancreatic beta cells to promote GLP-1R signaling and function

**Figure 2.**
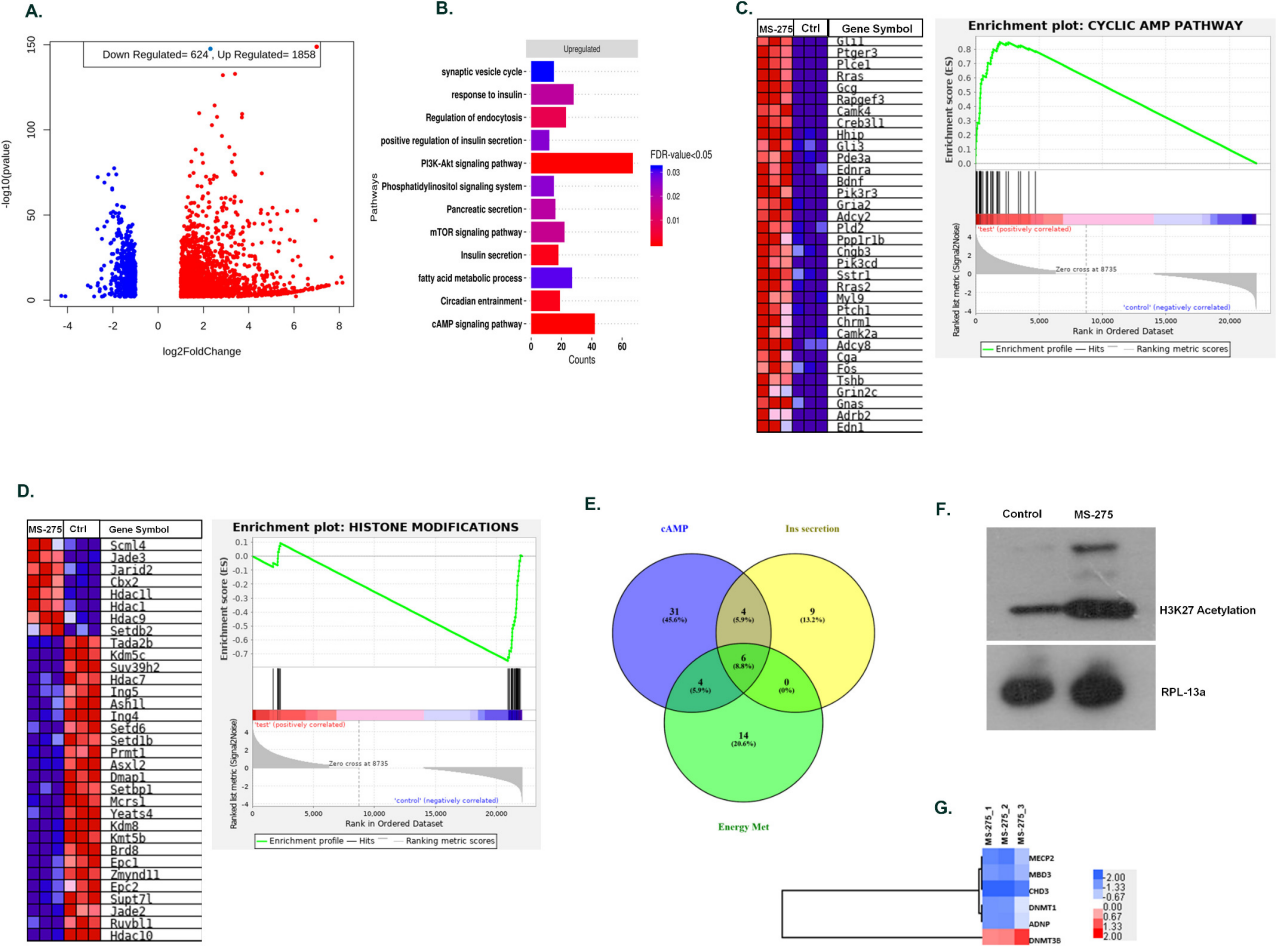
MS—275 alters transcriptome profile in BRIN-BD11 pancreatic beta cells. (**A**) Volcano plot from hierarchical clustering of differentially expressed genes on MS-275 treatment in BRIN-BD11 pancreatic beta cells. The log_2_ fold change is represented in the x-axis, whereas –log_10_ of the corrective p-value is represented in the y-axis. Red dots show upregulated while blue dots represent downregulated genes. (**B**) Gene ontology (GO) pathway enrichment upon MS-275 treatment in BRIN-BD11 pancreatic beta cells; only significantly enriched terms shown; FDR < 0.05. (**C**) GSEA Blue–Pink O’ gram of the cAMP pathway in the control and MS-275-treated pancreatic beta cells. Enrichment plot of the cAMP pathway in the control and MS-275 (test)-treated pancreatic beta cells depicting the profile of the running enrichment score (ES) and the position of the representative gene-set members in the rank order list. NES = Normalized enrichment score, FDR = False Discovery Rate. (**D**) GSEA Blue–Pink O’ gram of the genes related to Histone modifications in the control and MS-275-treated pancreatic beta cells. The graph represents the profile of the running enrichment score and positions of the Gene Set members in the rank order list. NES = Normalized enrichment score, FDR = False Discovery Rate. (**E**) Venn diagram of differentially expressed genes related to the cAMP-signaling pathway, insulin secretion pathway, and pathways involved in the energy metabolism. All Venn Diagrams were produced with Venny 2.0.2 (http://bioinfogp.cnb.csic.es/tools/venny/index.html). The numbers on the Venn diagram indicates the number of genes shared among the pathways. (**F**) Effect of MS-275 on H3K27 acetylation; the immunoblot images are representative of three independent experiments; RPL-13a immunoblot served as the loading control. (**G**) Differential Expressed Gene (DEG) heat map by GO terms of select genes related to chromatin modification in BRIN-BD11 pancreatic beta cells (log_2_ fold enrichment ≥2.0, p<0.05); upregulated genes in red, downregulated in blue. Figure 2—source data 1.Source Data Figure 2G.Western blot pictures (uncut) showing the impact of MS-275 on H3K27 acetylation; RPL-13a immunoblot served as the loading control.

We performed the mRNA sequence analysis to gain a deeper understanding of MS-275 -mediated regulation of GLP-1R signaling in BRIN-BD11 pancreatic beta cells. The experiment was conducted in triplicates (three control libraries treated with 0.01% DMSO versus three test libraries treated with 5 µM MS-275). There was *97***%** alignment of the reads to the reference rat genome. We found that *1858* genes were upregulated and 624 genes downregulated upon MS-275 treatment (log fold change ≥±2, p-value<0.05). The volcano plot (Figure 2A) provides an overview of the differentially expressed genes upon MS-275 treatment. Gene ontology analysis of the upregulated pathways included endocytosis, cAMP signaling, insulin secretion, and the PI3K-Akt signaling, whereas the downregulated genes include histone modification, chromosome organization, and cell cycle regulation (Figure 2B). Gene set enrichment analysis (GSEA) (classical scoring with 1000 permutations) revealed the cAMP signaling pathway to be significantly upregulated (FDR < 0.25) (Figure 2C). Differentially expressed genes (DEGs) of the pathways that regulate insulin secretion and glucose sensing also demonstrated a significant upregulation. In contrast, we observed a significant suppression of genes in the pathway related to histone modification (Figure 2D). The upregulation of the genes involving the cAMP pathway is in alignment with our experimental results of increased basal and incretin receptor-mediated cAMP generation upon MS-275 treatment. Venn diagram comparison between cAMP, insulin secretion, and energy metabolism pathways revealed modest gene sharing as shown in Figure 2E implicating the role of MS-275 in stimulating cAMP signaling pathway as well as regulating insulin secretion and energy homeostasis. The modulation of the transcriptome profile was reflected in the reversible chromatin alteration upon MS-275 treatment as has been manifested in increased H3K27 acetylation (Figure 2F) and in the alteration of the expression of the genes involved in global transcriptional regulation (Figure 2G) that has a significant influence on the GLP-1R signaling.

### MS-275 enhances the expression of genes involved in GLP-1R-mediated sustained cAMP generation

**Figure 3.**
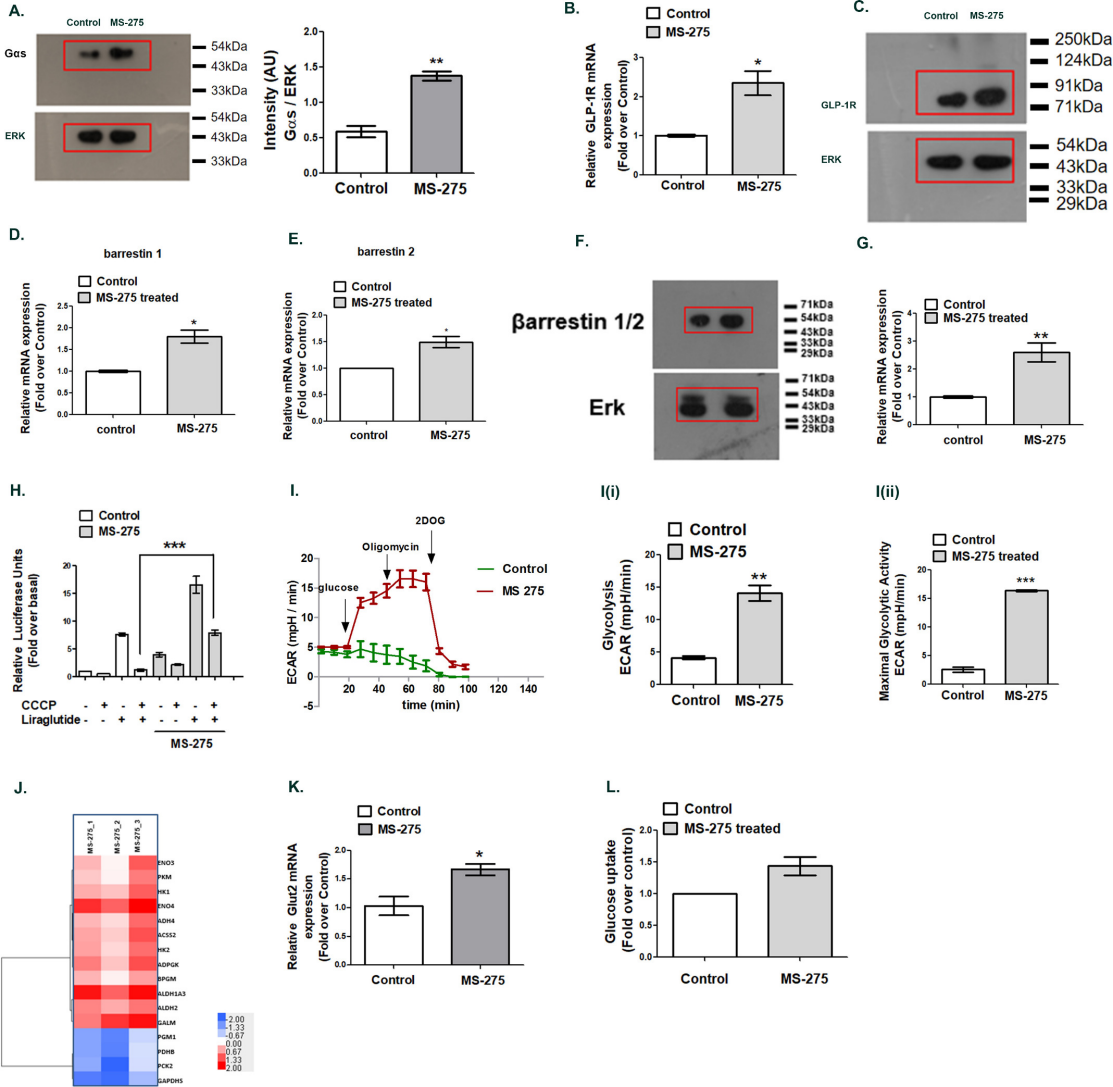
MS—275 augments GLP-1R-mediated cAMP generation. (**A**) Effect of MS-275 on Gαs protein expression; the immunoblot images are representative of three independent experiments; images being quantified using Image J. Total p44/42 (ERK) protein expression considered as the loading control; the data is quantified as the ratio of Gαs and total p44/42 expression (arbitrary units (AU)). **p<0.01 for Student’s t-test (unpaired) comparing the effect of MS-275 in control and MS-275-treated cells. (**B**) Effect of MS-275 on the mRNA expression of GLP-1R; the quantification being carried out using the 2^-ΔΔC^_T_ method and the data normalized using GAPDH as reference. Results are represented as the mean (± SE) of three independent experiments. *p<0.05 was determined using Welch’s t-test comparing the effect of MS-275 in control and MS-275-treated cells. (**C**) Effect of MS-275 on GLP-1R protein expression; the immunoblot images are representative of three independent experiments; total p44/42 (ERK) protein expression is considered as the loading control, the data being quantified as the ratio of GLP-1R and total p44/42 expression (arbitrary units(AU)). (**D**) Effect of MS-275 on the mRNA expression of beta arrestin1; the quantification being carried out using the 2^-ΔΔC^_T_ method and the data normalized using GAPDH as reference. Results are represented as the mean (± SE) of three independent experiments. *p<0.05 was determined by Welch’s t-test (unpaired) comparing the effect of MS-275 in control and MS-275-treated pancreatic beta cells. (**E**) Effect of MS-275 on the mRNA expression of beta-arrestin 2; the quantification being carried out using the 2^-ΔΔC^_T_ method and the data normalized using GAPDH as the reference. Results are represented as the mean (± SE) of three independent experiments. *p<0.05 was determined by Welch’s t-test (unpaired) comparing the effect of MS-275 in control and MS-275-treated pancreatic beta cells. (**F**) Effect of MS-275 on beta-arrestin protein expression; the immunoblot images are representative of three independent experiments; images being quantified using Image J. Total p44/42 (ERK) protein expression considered as the loading control. *p<0.05 for Student’s t-test (unpaired) comparing the effect of MS-275 in control and MS-275-treated pancreatic beta cells. (**G**) Effect of MS-275 on the expression of adenylyl cyclase 8; relative mRNA expression quantified using the 2^-ΔΔC^_T_ method and the data normalized using GAPDH as reference. Results are represented as the mean (± SE) of three independent experiments. **p<0.01 was determined by Welch’s t-test comparing the expression in control and MS-275-treated pancreatic beta cells. (**H**) Effect of chemical uncoupling of mitochondrial oxidative phosphorylation by CCCP (10 μM) on GLP-1R-mediated cAMP generation in control and MS-275-treated pancreatic beta cells measured by a luciferase reporter assay. Results represent mean (± SE) of four independent experiments and expressed as fold-over basal.***p<0.001 was determined by analysis of variance (ANOVA) using Tukey’s multiple comparison test comparing the impact of CCCP in control and MS-275-treated cells treated in the presence of liraglutide (**I**) The effect of MS-275 on the glycolytic activity is approximated by the Extracellular acidification rate (ECAR) in BRIN-BD11 pancreatic beta cells. I (i) represents glycolysis and I (ii) maximal glycolytic activity. Results are mean (± S.E) of three experiments ***p<0.001, **p<0.01 for Student’s t-test (unpaired) comparing cellular acidification parameters that approximate glycolysis. (**J**) Heat map of differentially expressed genes involved in glycolysis (log_2_ fold enrichment ≥2.0, p<0.05). Upregulated genes are in red and downregulated in blue. (**K**) Effect of MS-275 on GLUT2 mRNA expression; the relative mRNA expression quantified using the 2^-ΔΔC^_T_ method and the data normalized using 18S rRNA as reference. Results are represented as the mean (± SE) of three independent experiments. **p<0.01 was determined by Welch’s t-test comparing the expression in control and MS-275-treated pancreatic beta cells (**L**) Effect of MS-275 on Basal Glucose uptake in BRIN-BD11 pancreatic beta cells; results are mean (± S.E) of three independent sets of experiments carried out in replicate. Figure 3—source data 1.Source Data Figure 3A.Western blot pictures (uncut) showing the impact of MS-275 on Gαs protein expression; ERK immunoblot was considered as the loading control. Source Data Figure 3B: Source Data Figure 3C: Source Data Figure 3D and Figure 3E: Source Data Figure 3F: Source Data Figure 3G: Source Data Figure 3H: Source Data Figure 3K: Source Data Figure 3. Figure 3—source data 2.Source Data Figure 3B: Relative GLP-1R mRNA expression; the quantification was carried out using the 2^-ΔΔC^_T_ method and the data normalized using GAPDH as reference.Source Data Figure 3D and Figure 3E: Relative beta arrestin1 mRNA and beta-arrestin 2 mRNA expression; the quantification was carried out using the 2^-ΔΔC^_T_ method and the data normalized using GAPDH as reference. Source Data Figure 3G: Relative Adcy8 mRNA expression; the quantification was carried out using the 2^-ΔΔC^_T_ method and the data normalized using GAPDH as reference. Source Data Figure 3H: Effect of chemical uncoupling of mitochondrial oxidative phosphorylation by CCCP (10 μM) on GLP-1R-mediated cAMP generation in control and MS-275-treated cultured pancreatic beta cells. Source Data Figure 3K: Relative GLUT2 mRNA expression; the quantification was carried out using the 2^-ΔΔC^_T_ method and the data normalized using 18S rRNA as reference Source Data Figure 3L: Effect of MS-275 on Basal Glucose uptake in BRIN-BD11 pancreatic beta cells. Figure 3—source data 3.Source Data Figure 3C: Western blot pictures (uncut) showing the impact of MS-275 on GLP-1R protein expression; ERK immunoblot was considered as the loading control. Figure 3—source data 4.Source Data Figure 3F: Western blot pictures (uncut) showing the impact of MS-275 on beta-arrestin protein expression; ERK immunoblot was considered as the loading control.

We recently showed that the prolonged association of the Gα_s_ subunit of the heterotrimeric G proteins with the receptor-ligand complex at Rab5 endosomes contributes to the sustained GLP-1R signaling (Girada et al., 2017). The phenomenon was attributed to the formation of the megaplexes upon the association of Gα_s_, beta arrestin-1, adenylate cyclase, and the activated receptor at endosomes (Thomsen et al., 2016; Girada et al., 2017). To explain the molecular mechanism by which MS-275 treatment increased GLP-1R-mediated sustained cAMP generation, we explored the expression of the genes that participate in the process. As Figure 3A shows, MS-275 treatment significantly upregulated Gα_s_ protein expression (2.44 ± 0.45 fold; n = 3) in cultured pancreatic beta cells. The data aligned with the RNA seq data which showed the upregulation of Gnas gene expression (log_2_ fold 3.54; p adjusted 0.00003). We observed a significant increase in GLP-1R (Figure 3B and C) and beta-arrestin−1 expression as well (Figure 3D, Figure 3F) upon MS-275 treatment that participates in the process of sustained GPCR signaling from endosomes. The association of Gα_s_ GTP with the adenylyl cyclase is a prerequisite for the receptor-mediated cAMP generation. Various subtypes of adenylyl cyclase (Adcy1-8) that generate cAMP response upon GPCR activation have been reported in pancreatic beta cells (Leech et al., 1999; Roger et al., 2011; Kitaguchi et al., 2013). The isoform Adcy-8, essential for GLP-1R signaling (Roger et al., 2011) was enhanced upon MS-275 treatment (Figure 3G). As our data revealed, MS-275 treatment increased the Adcy8 expression by 2.59 ± 0.33 fold thereby defining the mechanism by which MS-275 enhanced the GLP-1R-mediated cAMP generation.

**Figure 3—figure supplement 1.**
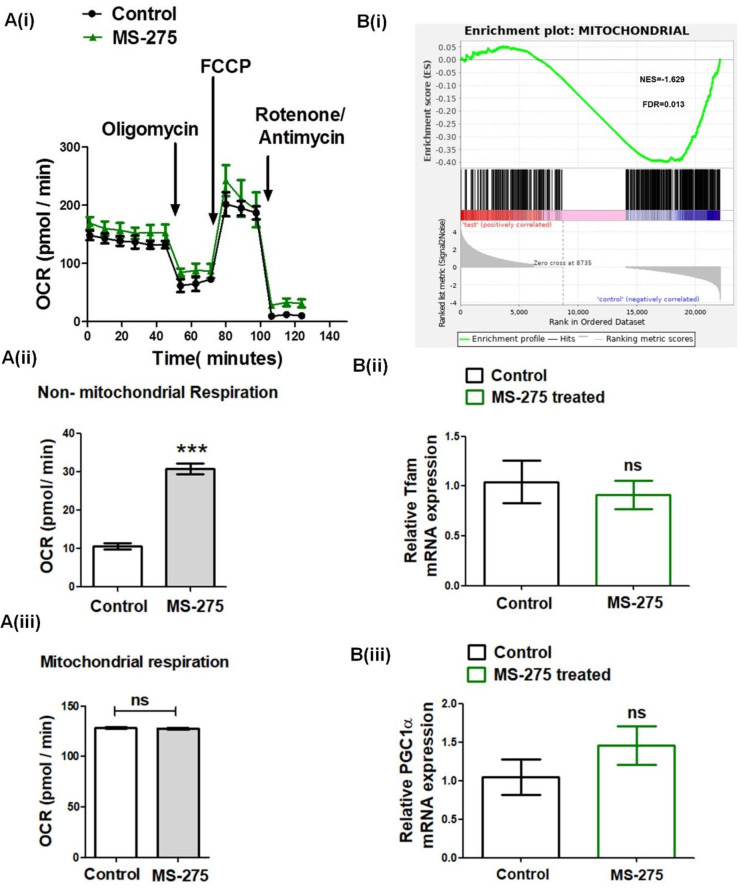
A (i) Real-time respirometry depicting oxygen consumption rate in the control and MS-275-treated pancreatic beta cells measured in Seahorse XF24 extracellular flux analyzer. The cellular respiration was dissected into non-mitochondrial (A (ii)) and mitochondrial respiration (A (iii)). Data are mean (± S.E) of three stimulated wells measured in triplicate. ***p<0.001, **p<0.01 for Student’s t-test (unpaired) comparing respiratory parameters in control and MS-275-treated cells; n.s = nonsignificant. B(i). GSEA of the mitochondrial genes in control and MS-275-treated pancreatic beta cells. The graph represents the profile of the running enrichment score and positions of the Gene Set members in the rank order list. NES = Normalized enrichment score, FDR = False Discovery Rate B (ii) The Effect of MS-275 on the mRNA expression of Tfam; the quantification being carried out using the 2^-ΔΔC^_T_ method and the data normalized using 18S rRNA as reference. Results are represented as the mean (± SE) of three independent experiments. ns = non-significant B(iii). The Effect of MS-275 on the mRNA expression of PGC-1α; the quantification was carried out using the 2^-ΔΔC^_T_ method and the data normalized using 18S rRNA as reference. Results are represented as the mean (± SE) of three independent experiments. ns: non-significant.

In the next step, we sought to determine the energy source for the MS-275 that promoted GLP-1R-mediated cAMP generation. GLP-1R signaling response was evaluated in the presence of chemical uncoupling agent carbonyl cyanide-4-(trifluoromethoxy) phenyl hydrazone (CCCP) that disrupts mitochondrial ATP synthesis. CCCP treatment completely abolished the GLP-1R agonism in control cells; in contrast, significant GLP-1R-mediated cAMP generation was retained in MS-275-treated pancreatic beta cells despite CCCP dosage (Figure 3H). These results suggested that MS-275-treated pancreatic beta cells, unlike the control cells, retain the substantial capacity to promote ATP synthesis even after the intensive mitochondrial chemical uncoupling. To explore it further, we carried out real-time respirometry that recorded a significant increase of glycolysis and maximal glycolytic activity upon MS-275 treatment (Figure 3I and I (i), 3*I* (ii)). A similar enhancement of non-mitochondrial respiration was observed without any alteration of mitochondrial respiration. (Figure 3—figure supplement 1). The mechanism by which MS-275 treatment enhanced the glycolysis is incompletely understood. Our RNA seq analysis in BRIN-BD11 cells revealed the enhancement of the genes involved in the glycolytic pathway upon MS-275 treatment (Figure 3J). We also observed a significant upregulation of GLUT2 mRNA expression upon MS-275 treatment (Figure 3K) and a corresponding increase in the basal glucose uptake (Figure 3L) that may account for the enhanced glycolysis to support the sustained cAMP generation in MS-275-treated cultured pancreatic beta cells.

### MS-275 potentiates GLP-1R-mediated GSIS

**Figure 4.**
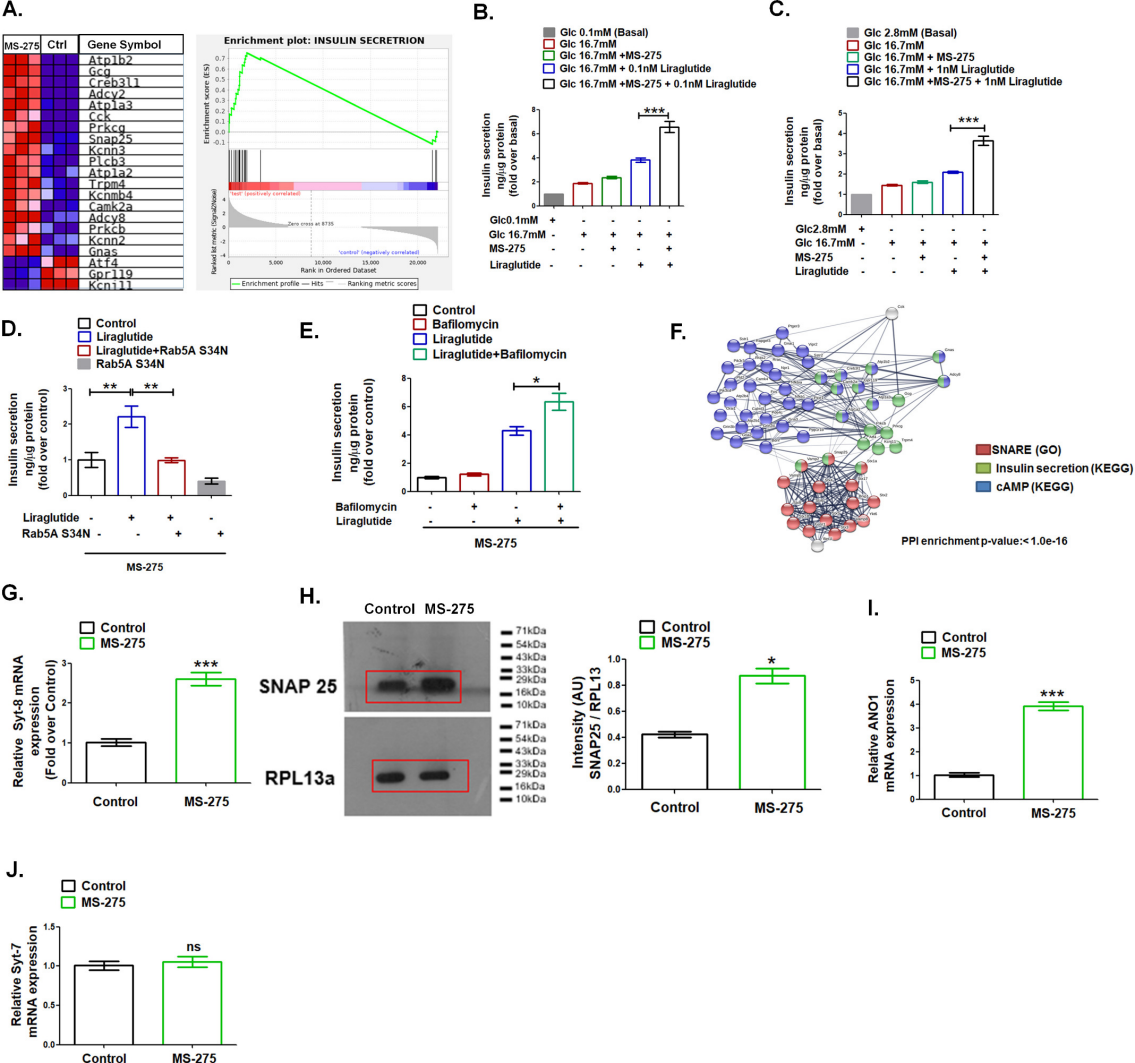
MS—275 stimulates GLP-1R-mediated GSIS and prevents fatty-acid-induced pancreatic beta-cell death. (**A**) GSEA Blue–Pink O’ gram of the representative genes involved in insulin secretion in control and MS-275-treated pancreatic beta cells. The graph depicting the profile of the running enrichment score (ES) and the position of the representative Gene Set members in the rank order list. NES = Normalized enrichment score, FDR = False Discovery Rate. (**B**) Effect of MS-275 on GLP-1R-mediated GSIS in BRIN-BD11 pancreatic beta cells. Insulin secretion is reported as ng/mg protein and expressed as fold over the basal secretion. Data are mean (± S.E) of three independent experiments; ***p<0.001 was determined by analysis of variance (ANOVA) using Tukey’s multiple comparison test comparing the effect of MS-275 on GSIS in the presence and absence of 0.1 nM liraglutide. (**C**) Effect of MS-275 on GLP-1R-induced GSIS mediated by 1 nM liraglutide in cultured rat islets. Results are the mean (± S.E) of three independent experiments; ***p<0.001 was determined by analysis of variance (ANOVA) using Tukey’s multiple comparison test comparing the effect of MS-275 on GSIS in rat islets in the presence and absence of liraglutide. (**D**) Effect of Rab5A S34N on GLP-1R-induced GSIS mediated by 1 nM liraglutide in MS-275-treated pancreatic beta cells. GSIS was evaluated in the presence of 16.7 mM glucose. Results are mean (± S.E) of three independent experiments; **p<0.01 was determined by analysis of variance (ANOVA) using Tukey’s multiple comparison test. (**E**) Effect of Bafilomycin on GLP-1R-induced GSIS mediated by 1 nM liraglutide in control and MS-275-treated pancreatic beta cells. Results are mean (± S.E) of three independent experiments; *p<0.05 was determined by the analysis of variance (ANOVA) using Tukey’s multiple comparison test. GSIS was evaluated in the presence of 16.7 mM glucose. (**F**) String analysis of DEGs related to the cAMP signaling cascade, SNARE, and insulin secretion pathway that is modulated upon MS-275 treatment. Networks in which there are overlaps between pathways based on the co-occurrence of genes are shown. Enrichment score: 1.0e-16. SNARE (GO) red; cAMP pathway (KEGG) blue; insulin secretion pathway (KEGG) green. The white/gray node indicates the second shell of interactors. (**G**) The effect of MS-275 on the mRNA expression of Syt-8; the quantification being carried out using the 2^-ΔΔC^_T_ method and the data normalized using 18S rRNA as reference. Results are represented as the mean (± SE) of three independent experiments. ***p<0.001 was determined by Welch’s t-test (unpaired) (**H**) Impact of MS-275 on SNAP 25 protein expression in cultured pancreatic beta cells. The immunoblot, representative of three independent experiments, is quantified using image J and the intensity (arbitrary units) is expressed as a ratio of SNAP 25 and RPL13a that serves as a loading control. Results are represented as the mean (± SE) of three independent experiments. *p<0.05 was determined by Welch’s t-test (unpaired) (**I**) The effect of MS-275 on the mRNA expression of Ano1; the quantification being carried out using the 2^-ΔΔC^_T_ method and the data normalized using 18S rRNA as reference. Results are represented as the mean (± SE) of three independent experiments. ***p<0.001 was determined by Welch’s t-test (unpaired) (**J**) The effect of MS-275 on the mRNA expression of Syt-7; the quantification being carried out using the 2^-ΔΔC^_T_ method and the data normalized using 18S rRNA as reference. Results are represented as the mean (± SE) of three independent experiments. ns: non-significant. Figure 4—source data 1.Source Data Figure 4B: The effect of MS-275 on GLP-1R-mediated GSIS in BRIN-BD11 pancreatic beta cells. Figure 4—source data 2.Source Data Figure 4H: Western blot pictures (uncut) showing the impact of MS-275 on SNAP 25 protein expression in cultured pancreatic beta cells; RPL-13a immunoblot was considered as the loading control.

GSEA analysis highlighted the upregulation of the genes involved in the insulin secretion pathway upon MS-275 treatment (Figure 4A, NES = 1.73). The observation guided us to interrogate the impact of the Class 1 HDAC inhibitor on GLP-1R-mediated GSIS. As Figure 4B revealed, MS-275 treatment amplified the GLP-1R-mediated insulin secretion in cultured pancreatic beta cells. Similarly, in rat islets, we observed a significant increase of GLP-1R-mediated insulin exocytosis (Figure 4C). MS-275-mediated augmentation of GLP-1R-induced GSIS was completely abrogated upon the expression of the Rab 5A S34N plasmid (Figure 4D) that reduced GLP-1R response (Figure 1H) and has earlier been reported to hinder GLP-1R internalization upon activation (Girada et al., 2017). Conversely, MS-275-treated cultured pancreatic beta cells, upon the exposure to Bafilomycin A1 significantly stimulated liraglutide-induced GSIS (Figure 4E). The data taken together signifies that MS-275-mediated augmentation of the sustained GLP-1R signaling, post-internalization of the receptor, translates to a physiological response in the form of enhanced GSIS. The string analysis of DEGs that includes SNARE, insulin secretion, and cAMP signaling revealed a significant interaction between these three pathways upon MS-275 treatment implying that the regulation may be at the level of the insulin vesicle fusion (Figure 4F). In agreement with the analysis, we observed MS-275-mediated upregulation of Synaptotagmin-8 (Syt-8) (Figure 4G), the t-SNARE SNAP25 (Figure 4H) as well as Anoctamin 1 (Ano 1) (Figure 4I), that regulate GSIS. (Xu et al., 2012; Al-Daghri, 2016; Crutzen et al., 2016), Though Syt-8 and SNAP 25 expression were upregulated, we did not observe any alteration in the expression of Synaptotagmin-7 (Syt-7) (Figure 4J), which is a PKA substrate and has been reported to contribute to the GLP-1R-induced GSIS (Wu et al., 2015). The data thus implies the existence of a unique SNARE complex that could promote GSIS upon the Class 1 HDAC inhibition.

### MS-275 upregulates the redox enzymes that prevent oxidative stress and palmitate-induced pancreatic beta-cell death

**Figure 5.**
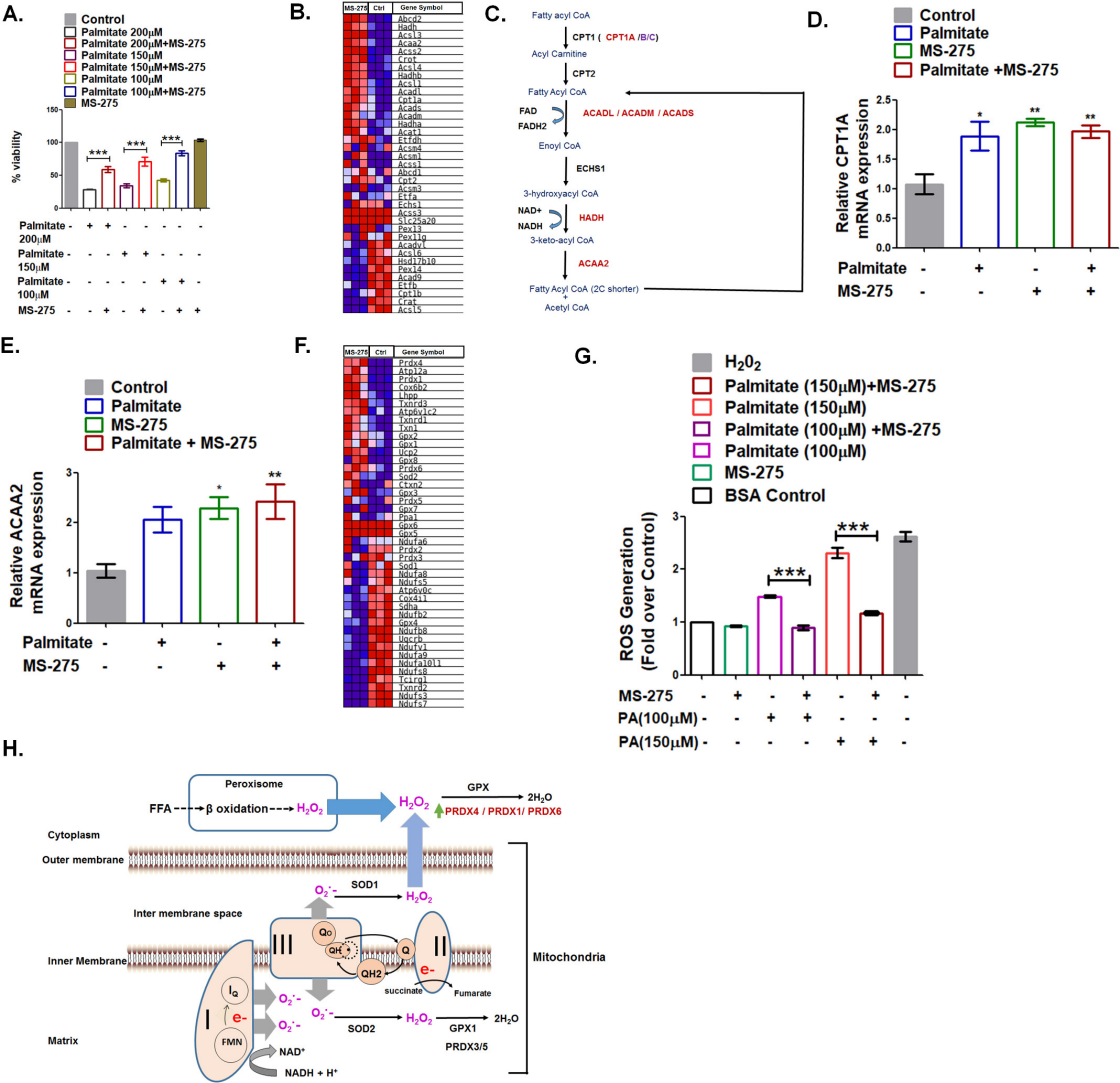
MS275 prevents palmitate-induced cell death in cultured pancreatic beta cells. (**A**) MS-275 treatment and its impact on palmitate-mediated pancreatic beta-cell death as assessed by MTT assay. Results are mean (± S.E) of three independent experiments; ***p<0.001 was determined by one-way ANOVA, Tukey’s multiple comparison test. (**B**) GSEA Blue–Pink O’ gram of the genes of the fatty acid degradation pathway in control and MS-275-treated pancreatic beta cells. The graph represents the profile of the running enrichment score and positions of the gene-set members in the rank order list. NES = Normalized enrichment score, FDR = False Discovery Rate. (**C**) Flow diagram of the β-oxidation pathway; the enzymes that are upregulated on MS-275 treatment are in red. (**D**) The Effect of palmitate on the mRNA expression of Cpt1A in the control and MS-275-treated pancreatic beta cells; the quantification being carried out using the 2^-ΔΔC^_T_ method and the data normalized using 18S rRNA as reference. Results are represented as mean (± SE) of three independent experiments; **p<0.01, *p<0.05 was determined by one way ANOVA, Tukey’s multiple comparison test. (**E**) The effect of palmitate on the ACAA2 mRNA expression in the control and MS-275-treated pancreatic beta cells; the quantification being carried out using the 2^-ΔΔC^_T_ method and the data normalized using 18S rRNA as reference. Results are represented as mean (± SE) of three independent experiments; **p<0.01, *p<0.05 was determined by one-way ANOVA, Tukey’s multiple comparison test. (**F**) GSEA Blue–Pink O’ gram of the representative antioxidant genes in the control and MS-275-treated pancreatic beta cells. (**G**) The effect of palmitate on ROS generation in the control and MS-275-treated pancreatic beta cells. The results are represented as mean (± SE) of three independent experiments; ***p<0.001, determined by one-way ANOVA, Tukey’s multiple comparison test. (**H**) Graphical representation of the generation of reactive oxidants upon free-fatty acid oxidation and their quenching upon MS-275-mediated upregulation of Prdx1, Prdx4, and Prdx6 genes in pancreatic beta cells. Figure 5—source data 1.a.Source Data Figure 5A.MS-275 treatment and its impact on palmitate-mediated pancreatic beta-cell death. b. Source Data Figure 5G: The effect of palmitate on ROS generation in control and MS-275-treated pancreatic beta cells.

A significant element in the pancreatic beta-cell health is its susceptibility to premature death (Rojas et al., 2018). Fatty acids constituted one of the prominent pathological factors that impact pancreatic beta-cell viability (Oh et al., 2018). The saturated fatty acid palmitate promotes fatty acid oxidation leading to the enhanced generation of reactive oxidants that causes lipotoxicity (Oprescu et al., 2007). We observed palmitate-induced death of cultured BRIN-BD11 pancreatic beta cells in a dose-dependent manner, which was prevented upon MS-275 treatment. (Figure 5A). These data in BRIN-BD11 pancreatic beta cells are in agreement with the observations reported in MIN-6 cells and human islets treated with MS-275 (Plaisance, 2014). In our present study, we explored the mechanism of prevention of lipotoxicity in pancreatic beta cells upon MS-275 treatment. GSEA analysis revealed significant upregulation of the key enzymes of the β-oxidation pathway like Cpt1A, ACADL, ACADM, ACADS, HADH, and ACAA2 when cultured pancreatic beta cells were treated with MS-275 (Figure 5B, Figure 5C). Since the β-oxidative activity was associated with increased superoxide generation (Rosca et al., 2012), oxidative stress, and apoptosis (Ye et al., 2019; Roma and Jonas, 2020), we compared the impact of palmitate on the expression of the genes involved in the pathway. As Figure 5D and Figure 5E revealed, there was a comparable expression of CPT1A and ACAA2 upon overnight 200 μM palmitate treatment implying comparable β oxidation flux in control, as well as in MS-275-treated pancreatic beta cells. However, higher expression of the redox enzymes like Prdx4, Prdx1, Prdx6 as well as Gpx2, Txnrd1, and Txnrd3 in the cytoplasm of the pancreatic beta cells was observed upon MS-275 treatment (Figure 5F), which is indicative of the increased neutralization of the reactive oxidants as explained in Figure 5H. In agreement with our RNA seq data, we observed decreased ROS generation in MS-275-treated cells upon palmitate exposure (Figure 5G) that may account for the reduced pancreatic beta cell death. The decreased pancreatic beta-cell death upon MS-275 treatment is reflective of the increased insulin content in pancreatic islets that we observed in the DIO mice receiving MS-275 monotherapy or MS-275 and liraglutide combined therapy.

### MS-275 promotes energy expenditure in cultured adipocytes

**Figure 6.**
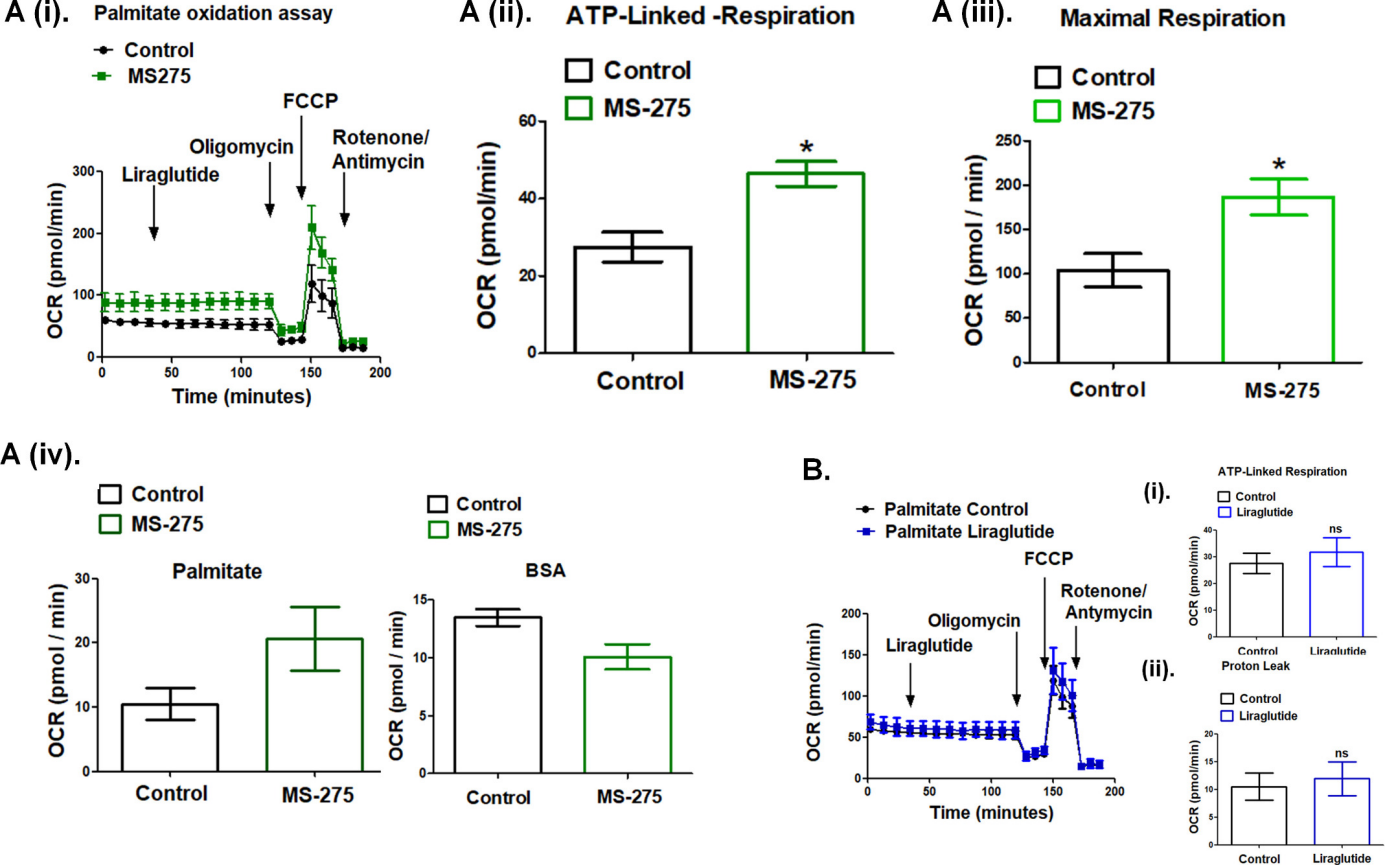
MS—275 promotes fatty acid oxidation in cultured adipocytes. (**A**) (**i**) Oxygen consumption rate (OCR) over time using palmitate (500 μM) as the substrate in the control and MS-275-treated cultured mouse adipocytes; after 30 min of recording the basal respiration, liraglutide (1 μM) was added and the OCR was recorded for another 90 min. Oligomycin (2 μM), FCCP (5 μM), and Rotenone/Antimycin (8 μM each) were added at 120, 145, and 170 min, respectively. (ii) ATP-linked Respiration in control and MS-275-treated 3T3L1 adipocytes as determined upon the addition of ATP-synthase inhibitor Oligomycin. Results were represented as mean (± SE); *p<0.05 was determined using Welch’s t-test. (iii) Maximal respiration in control and MS-275-treated 3T3L1 adipocytes that were obtained upon the addition of FCCP and subtracting non-mitochondrial respiration rates. Results are represented as mean (± SE); *p<0.05 was determined using Welch’s t-test. (iv) Proton leak in the control and MS-275-treated adipocytes derived by subtracting ATP-linked respiration from the mitochondrial respiration using (**i**) Palmitate (500 μM), as substrate and (ii) Fat-free BSA as the substrate. With palmitate as the substrate, the proton leak increased from 10.51 ± 2.43 pmol/min to 20.62 ± 4.97 pmol/min. (**B**) Oxygen consumption rate (OCR) over time using palmitate (500 μM) as the substrate in the control and liraglutide-treated cultured mouse adipocytes. (**i**) ATP-linked respiration and (ii) OCR linked to Proton leak is comparable in control adipocytes and upon liraglutide treatment.

The data presented in Figure 5 demonstrate the upregulation of the key enzymes of the β-oxidation pathway upon MS-275 treatment in cultured pancreatic beta cells. Previous reports described MS-275-mediated enhanced oxidative metabolism and white adipose tissue (WAT) browning (Galmozzi et al., 2013; Ferrari et al., 2017); however, the reports on the effect of GLP-1R signaling on energy expenditure were contradictory. Although in some studies GLP-1 analogs have been shown to contribute to fatty acid oxidation and WAT browning (Kooijman et al., 2015), other studies in the animal model and humans reported no change or even a decrease in the energy expenditure upon incretin treatment (van Eyk et al., 2020; Horowitz et al., 2012). In our present study, we addressed the mechanism of energy expenditure upon fatty acid oxidation in MS-275-treated cultured mouse adipocytes in the presence or absence of liraglutide. We carried out real-time respirometry in 3T3 L1 adipocytes using palmitate as the substrate to assess MS-275 and liraglutide-mediated energy expenditure (Figure 6A (i)). As the data shows, liraglutide exerted no effect on oxygen consumption rate (OCR) both in control and MS-275-treated cultured adipocytes when palmitate was used as substrate. We observed increased maximal respiration (Figure 6A (ii)) as well as ATP-linked respiration (Figure 6A (iii)) and the OCR linked to proton leak (Figure 6A (iv)) in MS-275-treated adipocytes. There was no such increase in cellular respiration Figure 6B (i), ATP-linked respiration (Figure 6B (ii)), or the proton leak (Figure 6B (iii)) upon over-night liraglutide treatment. Replacement of palmitate with the fat-free BSA diminished MS-275-mediated proton leak, which highlighted the contribution of the beta-oxidation pathway in promoting energy expenditure through proton leak in MS-275-treated cultured adipocytes.

### MS-275 improves the efficacy of liraglutide in enhancing glucose tolerance in diet-induced glycemic-impaired mice

**Figure 7.**
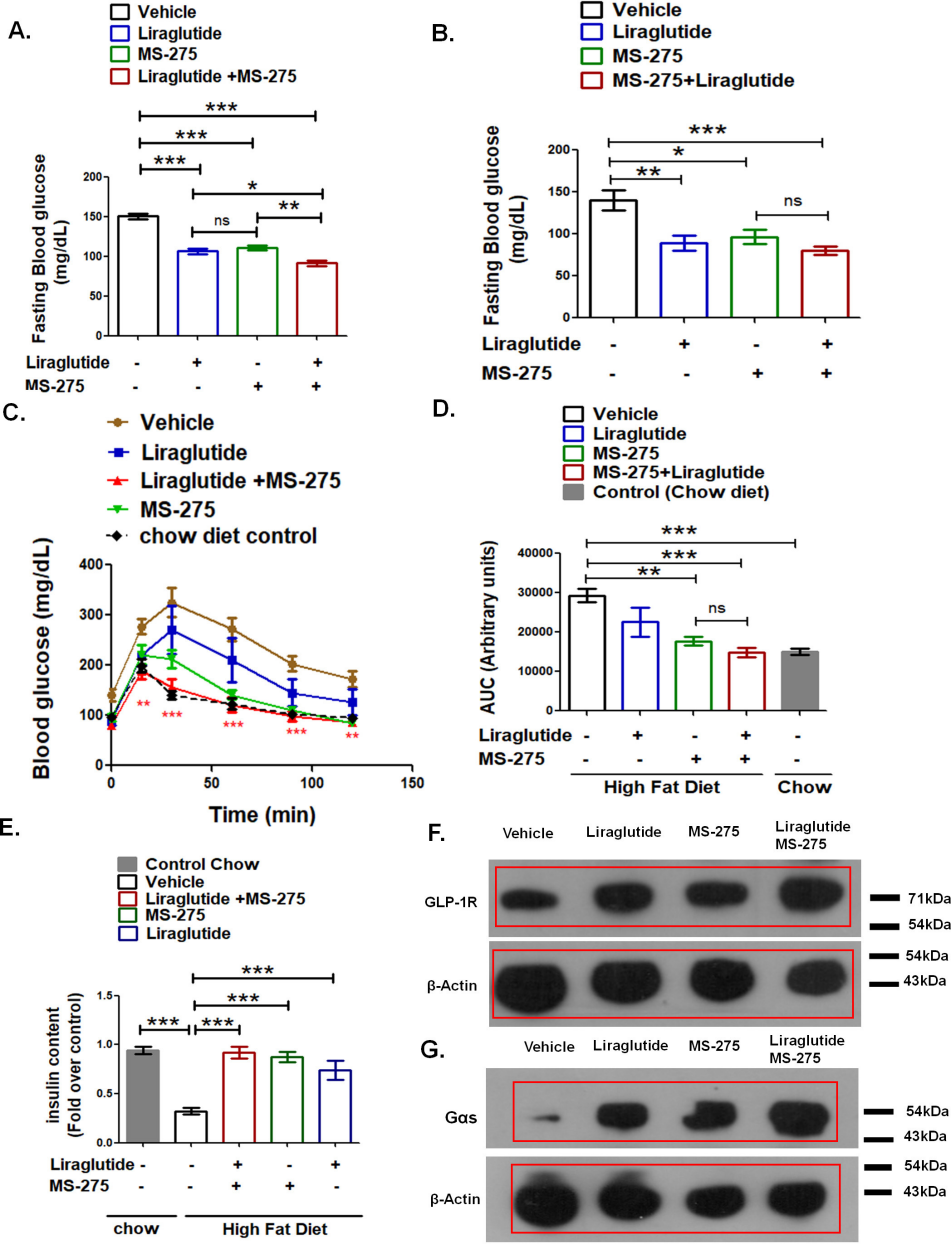
MS—275 enhances the efficiency of liraglutide in improving glucose tolerance. (**A**) Effect of acute MS-275 and liraglutide monotherapy versus combined therapy on the fasting blood sugar in C57BL/6 male mice fed on a high-fat diet (HFD). DIO mice received the intraperitoneal injection of MS-275 (5 mg/kg body weight every alternate day) or subcutaneous injection of liraglutide (3 nmol/kg body weight twice weekly) or a combination of the two drugs (n = 6 each group). The mice received a second dose of the injection after 24 h and fasted for 5 h following which the blood glucose was measured. Data represent mean ± S.E; ***p<0.001, **p<0.01, *p<0.05; as determined by one-way ANOVA, Tukey’s multiple comparison test; ns: non-significant (**B**) Effect of chronic MS-275 and liraglutide combined therapy on fasting blood sugar in C57BL/6 male mice fed on a HFD and receiving intraperitoneal injection of MS-275 (5 mg/kg body weight, every alternate day), subcutaneous injection of liraglutide (3 nmol/kg body weight twice weekly) or combined MS-275 and liraglutide co-therapy (n = 6 each group). Fasting blood glucose was measured at the end of the study. Data represent mean ± S.E.; ***p<0.001, **p<0.01, *p<0.05; as determined by one-way ANOVA, Tukey’s multiple comparison test; ns: non-significant (**C**) Intraperitoneal glucose tolerance test (IPGTT) in C57BL/6 male mice fed on the chow diet, HFD and receiving intraperitoneal injection of MS-275 (5 mg/kg body weight, every alternate day), subcutaneous injection of liraglutide (3 nmol/kg body weight twice weekly) or combined MS-275 and liraglutide co-therapy (n = 6 each group). Results represent mean ± S.E. ***p<0.001, **p<0.01, depicting the significant difference of glucose tolerance between the group receiving combined therapy versus the vehicle control at specific time points was determined by the analysis of variance (two-way ANOVA, Bonferroni posttests). (**D**) The AUC of the IPGTT in mice fed on chow diet, HFD and receiving intraperitoneal injection of MS-275 (5 mg/kg body weight, every alternate day), subcutaneous injection of liraglutide (3 nmol/kg body weight twice weekly) or combined MS-275 and liraglutide co-therapy (n = 6 each group). Data represent mean ± S.E.; ***p<0.001, **p<0.01, as determined by one-way ANOVA, Tukey’s multiple comparison test; ns = non significant. (**E**) Insulin content assessment in mice on chow diet, HFD and receiving an intraperitoneal injection of MS-275 (5mg/kg body weight, every alternate day), subcutaneous injection of liraglutide (3 nmol/kg body weight twice weekly) or combined MS-275 and liraglutide co-therapy (n = 5 each group) as evaluated by insulin ELISA. Data represent mean ± SE; ***p<0.001 as determined by one-way ANOVA, Tukey’s multiple comparison test. (**F**) GLP-1R Immunoblot from pooled pancreatic tissue isolated from DIO mice receiving vehicle, liraglutide, MS-275, and the combined therapy of liraglutide and MS-275. Beta-actin served as the loading control. (**G**) Gαs immunoblot from pooled pancreatic tissue isolated from DIO mice receiving vehicle, liraglutide, MS-275, and combined therapy of liraglutide and MS-275. Beta-actin served as a loading control. Figure 7—source data 1.a.Source Data Figure 7A.Effect of acute MS-275 and liraglutide monotherapy and combined therapy on fasting blood sugar in C57BL/6 DIO male mice b. Source Data Figure 7B: Effect of chronic MS-275 and liraglutide-combined therapy on fasting blood sugar in C57BL/6 male mice fed a high-fat diet. c. Source Data Figure 7C and Figure 7D: Intraperitoneal glucose tolerance test (IPGTT) in C57BL/6 male mice fed on chow diet, high-fat diet and receiving intraperitoneal injection of MS-275 (5mg/kg body weight, every alternate day), subcutaneous injection of liraglutide (3 nmol/kg body weight twice weekly) or combined MS-275 and liraglutide co-therapy. Figure 7—source data 2.a.Source Data Figure 7F.Western blot pictures (uncut) showing the impact of the vehicle, liraglutide, MS-275, and combined liraglutide and MS-275 co-therapy on GLP-1R protein expression in pancreatic tissue pooled from each group; beta-actin immunoblot served as the loading control. b. Source Data Figure 7G: Western blot pictures (uncut) showing the impact of the vehicle, liraglutide, MS-275, and combined liraglutide and MS-275 co-therapy on Gαs protein expression in pancreatic tissue pooled from each group. Beta-actin immunoblot served as the loading control.

**Figure 8.**
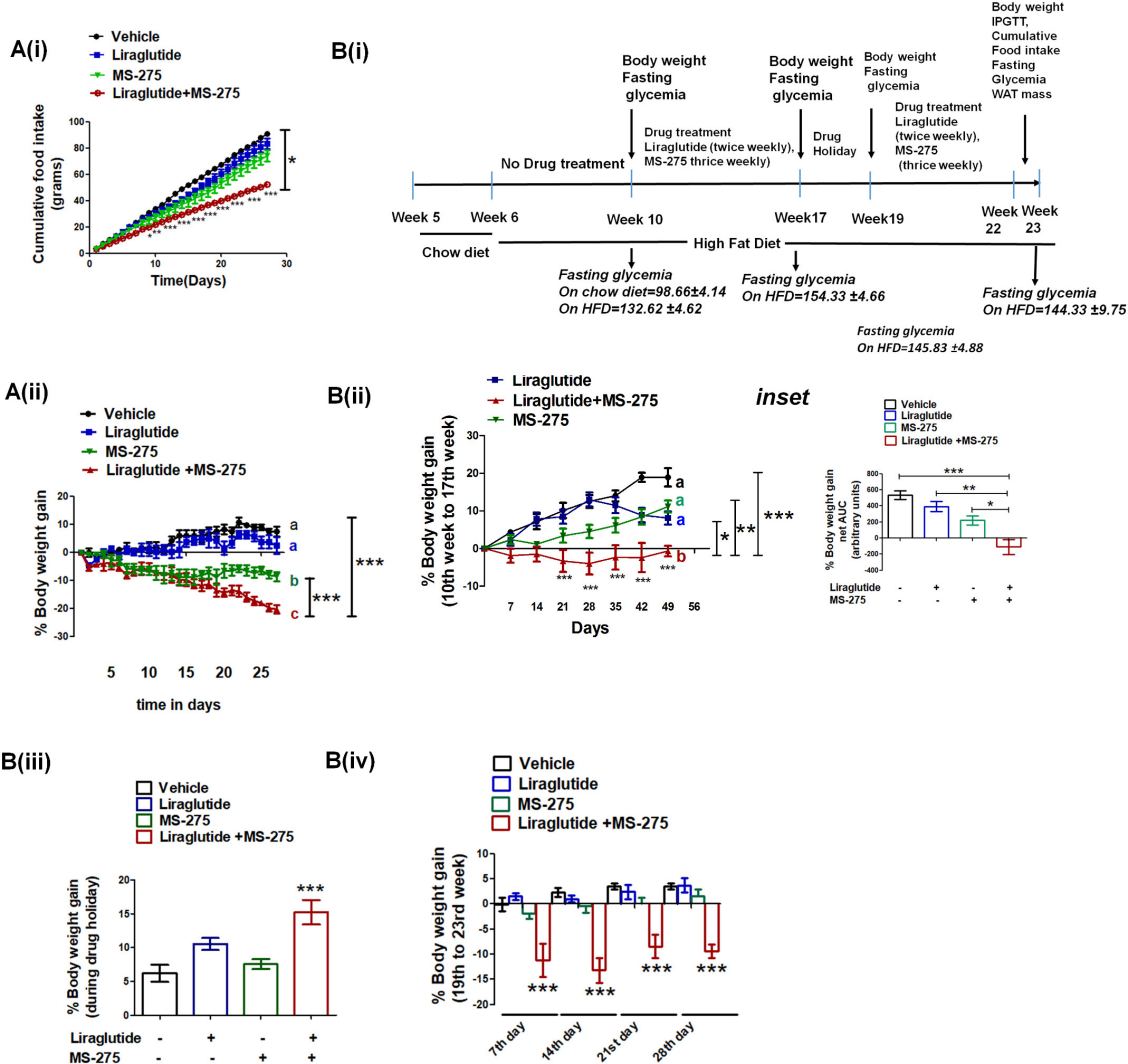
MS—275 and liraglutide reduces calorie uptake and decreases body weight gain. (**A**) Diet-induced obese (DIO) male mice were subjected to 4-week treatment with liraglutide (3 nmol/kg body weight twice weekly), MS-275 (5 mg/kg body weight, every alternate day), and a combined co-therapy of the two drugs. Fig A (i): Effects on the cumulative food intake measured every day. Results are mean ± S.E (n = 4). *p<0.05 as determined by one-way ANOVA, Tukey’s multiple comparison test. ***p<0.001, determined by analysis of variance (two-way ANOVA, Bonferroni post-tests) comparing between the groups receiving vehicle or liraglutide and MS-275 combined therapy at specific time points. Fig A (ii): Effects on the body weight gain measured every day. Results are mean ± S.E (n = 4); mean values with different letters are significantly different at ***p<0.001, one-way ANOVA, Tukey’s multiple comparison test. (**B**) (i) A flow diagram depicting the diet and the treatment regimen, as well as pharmacological parameters measured to assess the impact of liraglutide and MS-275 monotherapy and the combined co-therapy of the two drugs on the progression of diet-induced obesity; (ii) Effect of MS-275, or liraglutide monotherapy and co-therapy on the body weight gain in mice fed on the high-fat diet from the 10th week to the 17th week of their age (n = 6 per group). Data represent mean ± SE; mean values of respective treatment groups represented by different letters that indicate significant statistical difference (***p<0.001, **p<0.01,*p<0.05 as determined by one-way ANOVA, Tukey’s multiple comparison test). ***p<0.001; comparing vehicle and the group receiving liraglutide and MS-275 combined therapy at specific time points was determined by analysis of variance; (two-way ANOVA, Bonferroni posttests). *Inset:* The area under the curve (AUC) of the body weight gain in DIO mice receiving the intraperitoneal injection of MS-275 (5 mg/kg body weight, every alternate day), subcutaneous injection of liraglutide (3 nmol/kg body weight twice weekly) or combined MS-275 and liraglutide co-therapy (n = 6 each group). Data represent mean ± S.E.; ***p<0.001,*p<0.05, as determined by one-way ANOVA, Tukey’s multiple comparison test. (iii) The body weight gain during drug holiday (from week 17 to week 19); the chronic dosing was discontinued and mice were fed ad libitum with the high-fat diet. Body weight was assessed at the end of the drug holiday period. Results represent mean ± S.E, ***p<0.001 determined by one-way ANOVA, Tukey’s multiple comparison test. (iv) The body weight gain post drug holiday after reintroduction of MS-275, or liraglutide monotherapy relative to combined therapy from 19th to 23rd week. Body weight gain was assessed at the end of every week for 4 weeks. ***p<0.001 determined by analysis of variance (factorial ANOVA, Bonferroni post hoc tests) comparing body weight gain between the vehicle control group and the group receiving liraglutide and MS-275 combined therapy. Figure 8—source data 1.a. Source Data Figure 8A.Effect of 4-week treatment of DIO male mice with liraglutide (3 nmol/kg body weight twice weekly), MS-275 (5 mg/kg body weight, every alternate day), and a combined co-therapy of the two drugs on cumulative food intake and body weight gain. b. Source Data- Figure 8B, Impact of liraglutide and MS-275 monotherapy and the combined co-therapy of the two drugs on the progression of diet-induced obesity.

The ultimate therapeutic question pertains to whether these in vitro observations can translate to improved treatment of the disease. We compared the sustained effects of liraglutide and MS-275 on glucose tolerance in C57BL/6 mice fed on a high-fat diet (HFD) (Supplementary file 2-Table 2). We first assessed the acute impact of the liraglutide and MS-275 combinatorial treatment on fasting blood glucose. DIO mice were treated with a single dose of liraglutide (3 nmol/ kg body weight), or MS-275 (5 mg / kg body weight), or a combination of the two drugs at the indicated concentration. After 24 h, the animals received a second dose of the drugs following which they were fasted for 5 h and evaluated for blood glucose concentration. As Figure 7A revealed, the fasting blood sugar level in the control group is 150.2 ± 3.53 mg/dL, whereas upon the combined treatment of liraglutide and MS-275, the blood glucose is reduced to 91.33 ± 3.49 mg/dL (p<0.001, one way ANOVA, Tukey’s multiple comparison test). Although both monotherapy and combined therapy revealed a significant decrease in the blood glucose, mice receiving the combined therapy displayed a superior blood glucose reduction as compared to liraglutide monotherapy (p<0.05, one–way ANOVA Tukey’s multiple comparison test) or MS-275 monotherapy (p<0.01, one–way ANOVA Tukey’s multiple comparison test) (Figure 7A). To test whether this reduction of blood sugar level was sustained after repeated dosing, we administered liraglutide (3 nmol/kg body weight, twice weekly), MS-275 (5mg/kg body weight, thrice weekly), or a combination of the two entities following a dosing regimen provided in the Methods section as well as described in Figure 8B (i). The HFD elevated the fasting blood sugar in the control group to 140.5 ± 12.13 mg/dL as compared to 80.0 ± 4.69 mg/dL in the group on the combined treatment of MS-275 with liraglutide (p<0.001, one-way ANOVA, Tukey’s Multiple Comparison Test). The corresponding fasting blood glucose in the groups receiving MS-275 or liraglutide monotherapy was 96.5 ± 8.63 mg/dL (p<0.05, one-way ANOVA, Tukey’s Multiple Comparison Test) and 89.17 ± 8.77 mg/dL (p<0.01, one-way ANOVA, Tukey’s Multiple Comparison Test) respectively (Figure 7B). The data thus demonstrates efficient blood glucose reduction both by monotherapies as well as by the combined therapy upon repeat dosing. The reduction in fasting blood glucose was consistent with the restoration of normal glucose tolerance in the mice that received the MS-275 monotherapy as well as liraglutide and MS-275 combined therapy (Figure 7C). As the corresponding area under the curve (AUC) revealed, the group that received the MS-275 and liraglutide combined therapy showed statistically significant improved glycemic control as compared to the group receiving normal saline as the vehicle (reduction from AUC 29322 ± 1764 arbitrary units to AUC 14809 ± 1261 arbitrary units; p<0.001, Tukey’s multiple comparison test; Figure 7D). Glucose tolerance was also improved with MS-275 monotherapy as compared to vehicle (reduction from AUC 29322 ± 1764 arbitrary units to AUC 17733 ± 1108 arbitrary units; p<0.01, Tukey’s multiple comparison test; Figure 7D) demonstrating that both MS-275 monotherapy as well as liraglutide and MS-275 combined therapy are efficient in improving glycemic control in DIO rodent model. To explore the mechanism, we assessed the insulin content in the pancreatic tissue isolated from the chow diet-fed animals as well as from the mice on HFD receiving liraglutide and MS-275 monotherapy or the combined therapy or the vehicle alone. As Figure 7E shows, HFD feeding significantly reduced the insulin content as compared to the group on the chow diet, which is restored upon treatment with liraglutide or MS-275 monotherapy as well as liraglutide and MS-275 combined therapy. In coordination with the increase in the insulin content, MS-275 and liraglutide combined treatment enhanced the expression of the key proteins involved in the GLP-1R signaling pathway in vivo. As our data showed, the increase of GLP-1R and Gαs was the highest in mice treated with liraglutide and MS-275 combined therapy (Figure 7F and Figure 7G). These results demonstrated the mechanism for successful translation of the in vitro observations to improved glycemic control in HFD-fed mice with impaired glucose tolerance.

### MS-275 and liraglutide combined therapy decreased food uptake and reduced body weight gain in DIO mice

Based on the enhanced efficacy of the combinatorial therapy of liraglutide and MS-275 in attaining glycemic control, we assessed its effect in reducing diet-induced obesity. We treated DIO mice with normal saline that serves as a vehicle, liraglutide (3 nmol kg^−1^ body weight, twice weekly), MS 275 (5 mg kg^−1^ body weight every alternate day), and a combination of the two drugs and measured body weight gain and food intake every day for a period of 4 weeks. As Figure 8A (i) shows, there is a significant reduction of the cumulative food intake in the mice receiving combined therapy of liraglutide and MS-275 (p<0.05 one-way ANOVA, Tukey’s multiple comparison test). The decrease in the food intake contributes to a negative energy balance resulting in a significant weight loss as we observed a reduction of the body weight gain in mice receiving the dual therapy of liraglutide and MS-275 (Figure 8A (ii), p<0.001 two way ANOVA Bonferroni post-tests). To assess the impact of the therapy on the disease progression and attainment of the DIO phenotype, we acclimated C57BL/6 mice to the HFD for 4 weeks (dosing regimen described in Figure 8B (i)). Following acclimatization, mice were administered with liraglutide (3 nmol kg^−1^, twice a week) along with MS-275 (5 mg kg^−1^, every alternate day) for a period of 7 weeks and were compared for the body weight gain against vehicle control or groups receiving monotherapy of either of the two drugs. Control mice and the groups receiving liraglutide or MS-275 monotherapy showed increased body weight gain. However, in the combined treatment group there was a significant decrease in body weight (Figure 8B ii), (p<0.001, one-way ANOVA, Tukey’s multiple comparison test).

In the next step, we provided a 2-week treatment holiday to all mice in which they continued on ad libitum HFD. In this period, the mice that received combined therapy had enhanced body weight gain relative to their comparators that received liraglutide or MS-275 monotherapy (Figure 8B (iii)). We interpret this result to reflect on the reversibility of the combined treatment, without any signs of acute or chronic safety in the treated mice. After 2 weeks without treatment, at a point when all groups were once again of comparable body weight, treatment was restored for another 4 weeks (Figure 8B (iv)). We measured the weight gain every week and as the data shows, only the group receiving MS-275 and liraglutide combined therapy significantly reduced the body weight (p<0.001 two-way ANOVA Bonferroni post-tests). The data aligns with The SCALE Maintenance randomized clinical study where similar weight gain was noticed after the withdrawal of liraglutide during follow-up after 56-week treatment (Wadden et al., 2013).

### Liraglutide and MS-275 combined therapy decrease visceral adiposity

**Figure 9.**
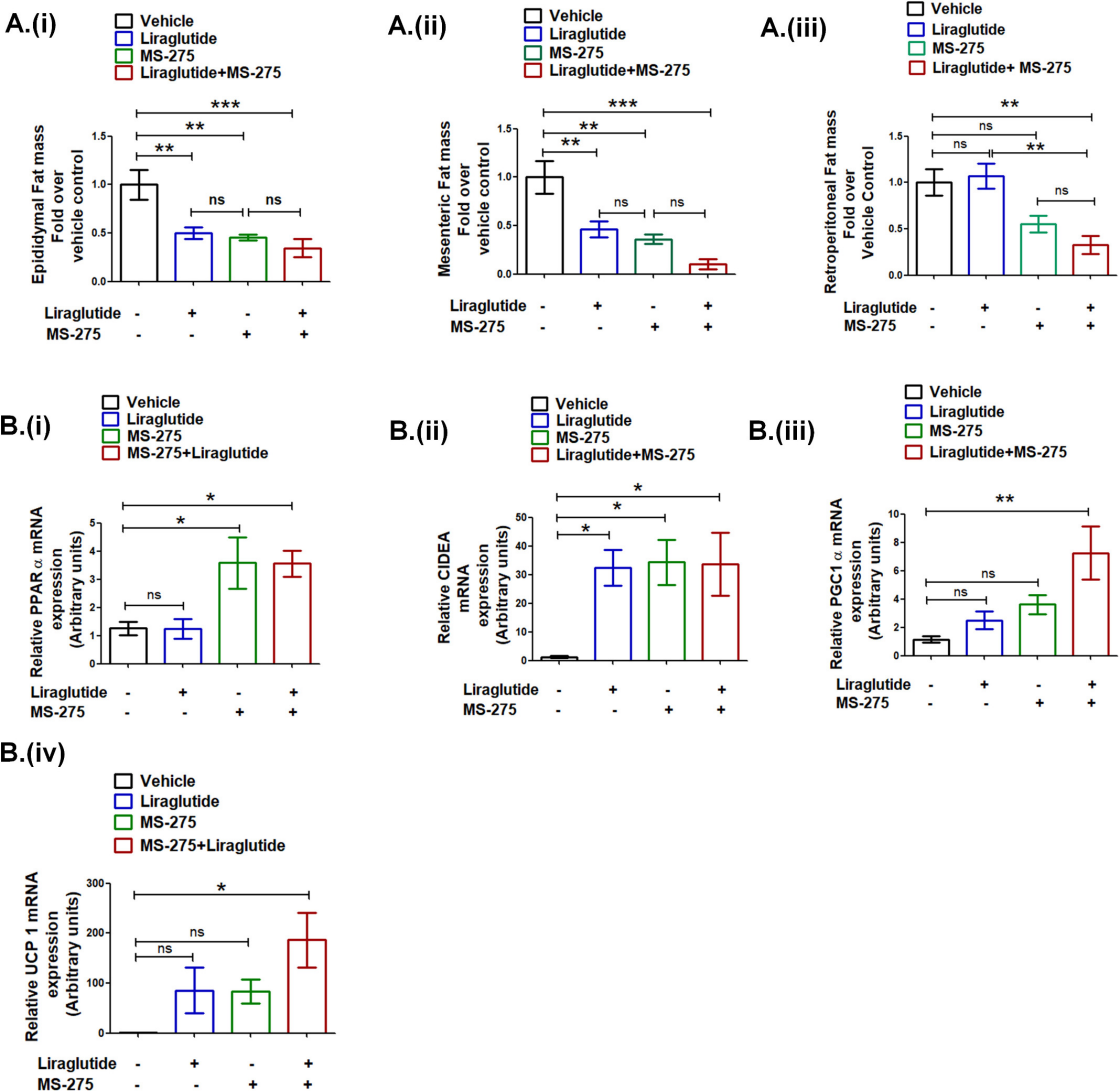
Liraglutide and MS-275 combined therapy reduces visceral obesity. (**A**) The effect of liraglutide and MS-275 monotherapy and the combined co-therapy of the two drugs on (i) epididymal fat mass, (ii) mesenteric fat mass, and (iii) retroperitoneal fat mass; the data expressed as fold over the vehicle control represent mean ± S.E; ***p<0.001,**p<0.01, as determined by one-way ANOVA, Tukey’s multiple comparison test; ns: non-significant. (**B**) The effect of liraglutide and MS-275 monotherapy and the combined co-therapy of the two drugs on (i) PPAR α mRNA expression, (ii) CIDEA mRNA expression, (iii) PGC1α expression, and (iv) UCP1 expression in retroperitoneal WAT as quantified using the 2^-ΔΔC^_T_ method. Results are normalized using 18s rRNA as a reference and represent mean ± S.E. **p<0.01, *p<0.05, as determined by one-way ANOVA, Tukey’s multiple comparison test; ns = non significant. Figure 9—source data 1.a Source data Figure 9A.Effect of MS-275, or liraglutide monotherapy and combined therapy on white adipose tissue mass; (i) epididymal, (ii) retroperitoneal, and (iii) mesenteric WAT in DIO mice b. Source data Figure 9B: Effect of MS-275, or liraglutide monotherapy and combined therapy on relative mRNA expression of PPARα (i), CIDEA (ii), PGC1α (iii), and UCP1 (iv) in retroperitoneal WAT of HFD mice that were quantified using the 2^-ΔΔC^_T_ method.

Having established the decrease in the body weight gain upon combined therapy of liraglutide and MS-275, we sought to ascertain the impact on visceral adiposity. Our data showed a significant reduction of the epididymal WAT upon MS-275 and liraglutide combined therapy as compared to the vehicle control (34.6 ± 0.09%; p<0.001, one-way ANOVA, Tukey’s multiple comparison test) (Figure 9A (i)). The corresponding reduction of the epididymal WAT was 45.3 ± 0.03% and 50.1 ± 0.06% in the case of MS-275 and liraglutide monotherapy, respectively (p<0.01, one way ANOVA, Tukey’s multiple comparison test). The mesenteric WAT in mice upon liraglutide and MS-275 combined therapy was reduced to 10.36 ± 0.05% as compared to the group receiving normal saline as vehicle control (p<0.001, one-way ANOVA, Tukey’s multiple comparison test) (Figure 9A (ii)). In the case of liraglutide and MS-275 monotherapy, mesenteric WAT was reduced to 46.1 ± 0.08% and 36.1 ± 0.04%, respectively (p<0.01, one-way ANOVA, Tukey’s multiple comparison test). The data demonstrate that both monotherapies and the combined therapy reduce epididymal and mesenteric WAT mass as compared to vehicle control. In contrast, we observed no reduction of retroperitoneal WAT mass upon liraglutide monotherapy, whereas upon combined therapy with liraglutide and MS-275 there was a reduction to 32.97 ± 0.09% as compared to the control group receiving normal saline as vehicle control (p<0.01, one-way ANOVA Tukey’s multiple comparison test). The reduction of retroperitoneal WAT upon MS-275 monotherapy is 55.09 ± 0.09% as compared to vehicle control. The results taken together revealed the comparable efficacy of MS-275 monotherapy and liraglutide and MS-275 combined therapy in decreasing visceral obesity in the DIO rodent model. We enquired whether the significant reduction of the retroperitoneal WAT upon liraglutide and MS-275 combined therapy is associated with the expression of genes involved in the energy homeostasis (sequence of the primers described in Supplementary file 3 Table 3). The significant increase in PPAR α gene expression vis-à-vis vehicle control is comparable between the groups on MS-275 monotherapy and MS-275 and liraglutide combined therapy, liraglutide monotherapy was ineffective in altering PPAR α gene expression at the indicated dose (Figure 9B (i)). The increase in CIDEA (Cell Death-Inducing DFFA-like effector A) gene expression is comparable among mice on liraglutide or MS−275 monotherapy as well as the group receiving combined therapy (Figure 9B (ii)). However, we observed a significant increase of PGC1 alpha (Figure 9B (iii)) and UCP1 (Fig 9B (iv)) in retroperitoneal WAT upon MS-275 and liraglutide combined treatment as compared to the vehicle control that exceeds either of the two monotherapies. The data highlight that in addition to its effect on the calorie uptake, liraglutide, and MS-275 combinatorial therapy has a significant impact on the expression of the genes involved in regulating energy homeostasis in visceral adipose tissue.

**Figure 9—figure supplement 1.**
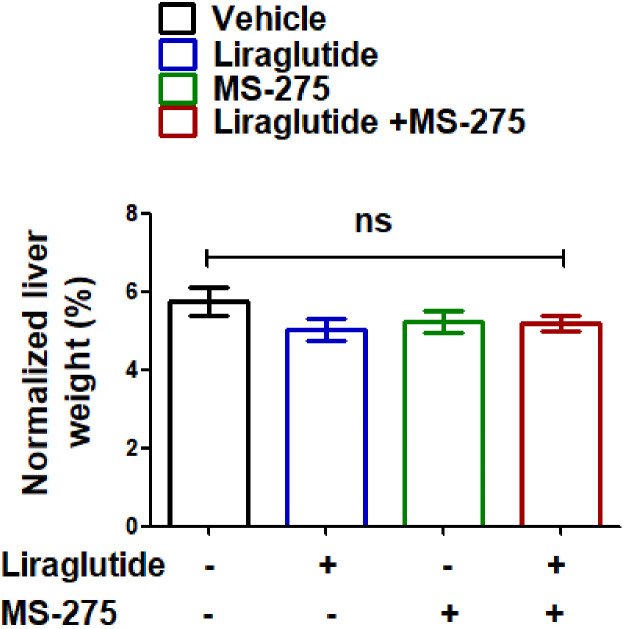
Effect of MS-275, liraglutide monotherapy, and combined therapy or the vehicle control on the liver weight, the data represent mean ± S.E; ns: non-significant.

**Figure 9—figure supplement 2.**
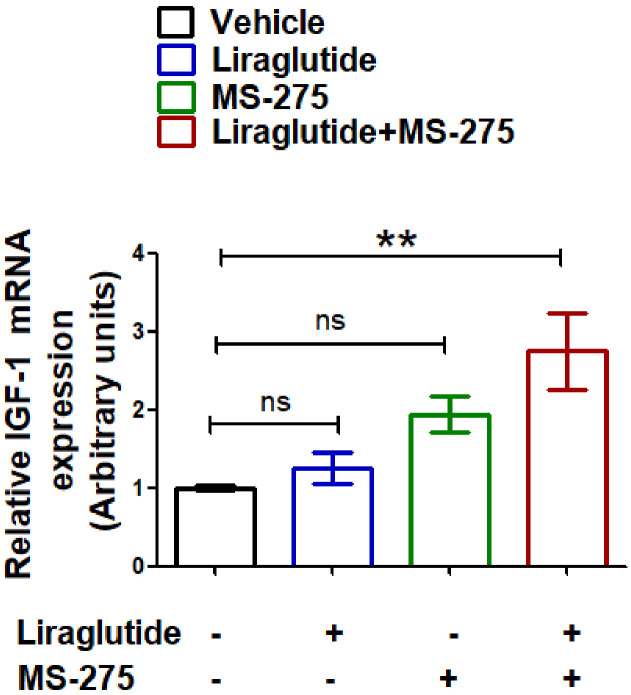
Effect of MS-275, liraglutide monotherapy, and combined therapy or the vehicle control on the IGF1 expression in liver, n = 4; the data represent mean ± S.E. **p<0.01, as determined by one-way ANOVA, Tukey’s multiple comparison test; ns: non-significant.

## Discussion

T2D is a metabolic disease characterized by impaired cellular signaling that affects insulin secretion in pancreatic beta cells and impedes insulin signaling and energy homeostasis at specific peripheral target tissues. The disease is heterogeneous and polygenic (Asalla et al., 2016), and with high familial risk (van Tilburg et al., 2001). The pathophysiology thus represents a complex interaction of the susceptible genes and the environment through epigenetic modifications that influence its occurrence and progression (Raciti et al., 2015). GLP-1R agonists have emerged in the last decade as unique medicines that provide substantial improvements in glycemic control and much-needed improvements in body weight. However, existing therapy seldom achieved full recovery of the glucose levels and even less so the associated abnormalities, especially the excess body weight. Consequently, given the epidemic nature of the disease, the search for the mechanisms in therapeutic action that could beneficially complement incretin action is of great interest (Tschöp et al., 2016; Müller et al., 2018). The findings we report herein suggest an alternative approach to enhance the incretin-based efficacy by regulating the expression of the genes that govern the incretin action through epigenetic modifications.

Through a cell-based assay, unbiased to a specific mechanism of action but directed to small molecules capable of potentiating GLP-1R action in pancreatic beta cells, a small set of Class 1 HDAC inhibitors were identified, of which MS-275 was the most effective. Enhanced GLP-1R action was specific for the post-internalization activated state of the receptor as the inhibition of internalization diminished MS-275-mediated augmentation of GLP-1R signaling. Conversely, the blunting of the endosome maturation enhanced MS-275-induced GLP-1R response that signifies the upregulation of the receptor agonism, while the activated receptor remained internalized at the endosomes. Our present study has shown that MS-275 increased the expression of the GLP-1 receptor, Gα_s_ subunit of the heterotrimeric G protein as well as beta arrestin-1, which are known to participate in the process of sustained endosomal cAMP generation (Thomsen et al., 2016; Girada et al., 2017). The study thus provides the first documentation of a small-molecule-mediated augmentation of sustained endosomal cAMP generation in the context of its regulation of GSIS in pancreatic beta cells.

Coincident with the increase in the Gα_s_ expression, we observed the increase in the expression of Adcy-8; a downstream mediator crucial for GLP-1R-mediated cAMP generation. DNA methylation but not acetylation is known to regulate the Adcy-8 promoter activity in peripheral blood monocytes (Gunawardhana et al., 2014), as well as in high-grade cervical cancers (Shen-Gunther et al., 2016). The RNA seq data presented in this study revealed a decrease in the expression of DNMT-1 gene expression upon MS-275 treatment (log _2_ fold −0.82 p adjusted: 5.38E-17; Figure 2G and H) that aligned with the reduction in the relative presence of methyl cytosine as observed in the case of the calcium-sensing receptor (CaSr) promoter (Alaminos et al., 2004). Our data is indicative of a similar regulatory process for the MS-275-mediated enhancement of Adcy-8 expression in the pancreatic beta cells.

Contextual to the augmentation of sustained cAMP generation upon GLP-1R activation, our previous study, using TIRFM, has demonstrated the regulation of insulin secretion at the level of insulin vesicle fusion (Girada et al., 2017). Our present study has shown that MS-275 treatment modulates the gene expression of the SNARE complex that participates in insulin vesicle fusion. While the expression of the PKA substrate SNAP-25 was enhanced (Figure 4E), we observed no alteration of Synaptotagmin 7 (Figure 4J) that mediated GLP-1R-stimulated GSIS (Wu et al., 2015). Instead, the expression of Synaptotagmin 8 (Figure 4D) and ANO-1 (Figure 4F) was upregulated indicating the formation of a new SNARE interaction upon MS-275 treatment. The exploration of the detailed mechanism, however, would be relevant to a future assessment focused on SNARE regulation upon HDAC1 inhibition.

Our data also described the increase in insulin content upon MS-275 treatment in DIO mice. As a possible mechanism, we observed a substantial reduction of cell death when MS-275-treated BRIN-BD11 pancreatic beta cells were exposed to palmitate. MS-275-mediated prevention of lipotoxicity has previously been reported in MIN-6 and human islets (Plaisance, 2014), although the mechanism was incompletely understood. We observed comparable expression of the rate-limiting enzyme CPT1A and the other key enzymes of the beta-oxidation pathway upon overnight palmitate treatment that implied comparable beta-oxidation flux in the control and MS-275-treated pancreatic beta cells. However, GSEA presented in this study revealed the upregulation of the antioxidant enzymes upon MS-275 treatment (Figure 5F) that prevents the fatty-acid-induced lipotoxicity. Accordingly, we observed decreased ROS production in MS-275-treated cells upon palmitate exposure (Figure 5G) thereby providing the mechanism for MS-275-mediated prevention of pancreatic beta-cell death. Furthermore, altered mitochondrial dynamics and enhanced fragmentation due to fatty acid stress had been linked to pancreatic beta-cell death (Wiederkehr and Wollheim, 2009). As our data reveals, MS-275 partially alleviated the loss of GLP-1R signaling upon the treatment with chemical uncoupler CCCP that caused extensive mitochondrial fragmentation. The observation implies a possible mechanism in the prevention of fatty-acid-induced cell death through the reduction of the reactive oxidants and the preservation of mitochondrial dynamics that may play a crucial role in the preservation of the pancreatic beta-cell mass.

The translation of improved in vitro GLP-1R signaling was extended to in vivo study to determine whether suboptimal therapy with liraglutide, a well-validated specific GLP-1 agonist might provide better metabolic outcomes upon co-treatment with MS-275. We observed enhanced GLP-1R (Figure 7F) and Gαs expression (Figure 7G) in the pancreatic tissue isolated from the mice that received combined MS-275 and liraglutide therapy. Besides, the insulin content was significantly enhanced (Figure 7E) upon MS-275, liraglutide, as well as liraglutide and MS-275 combined therapy which may contribute to the normalization of the acute and chronic fasting blood sugar (Figure 7A–D). MS-275 monotherapy also exhibits improved glycemic control. We hypothesize that the upregulation of incretin receptor signaling upon MS-275 treatment might affect the entero-insular axis and subsequent regulation of incretin action that deserves attention in future research.

**Figure 10.**
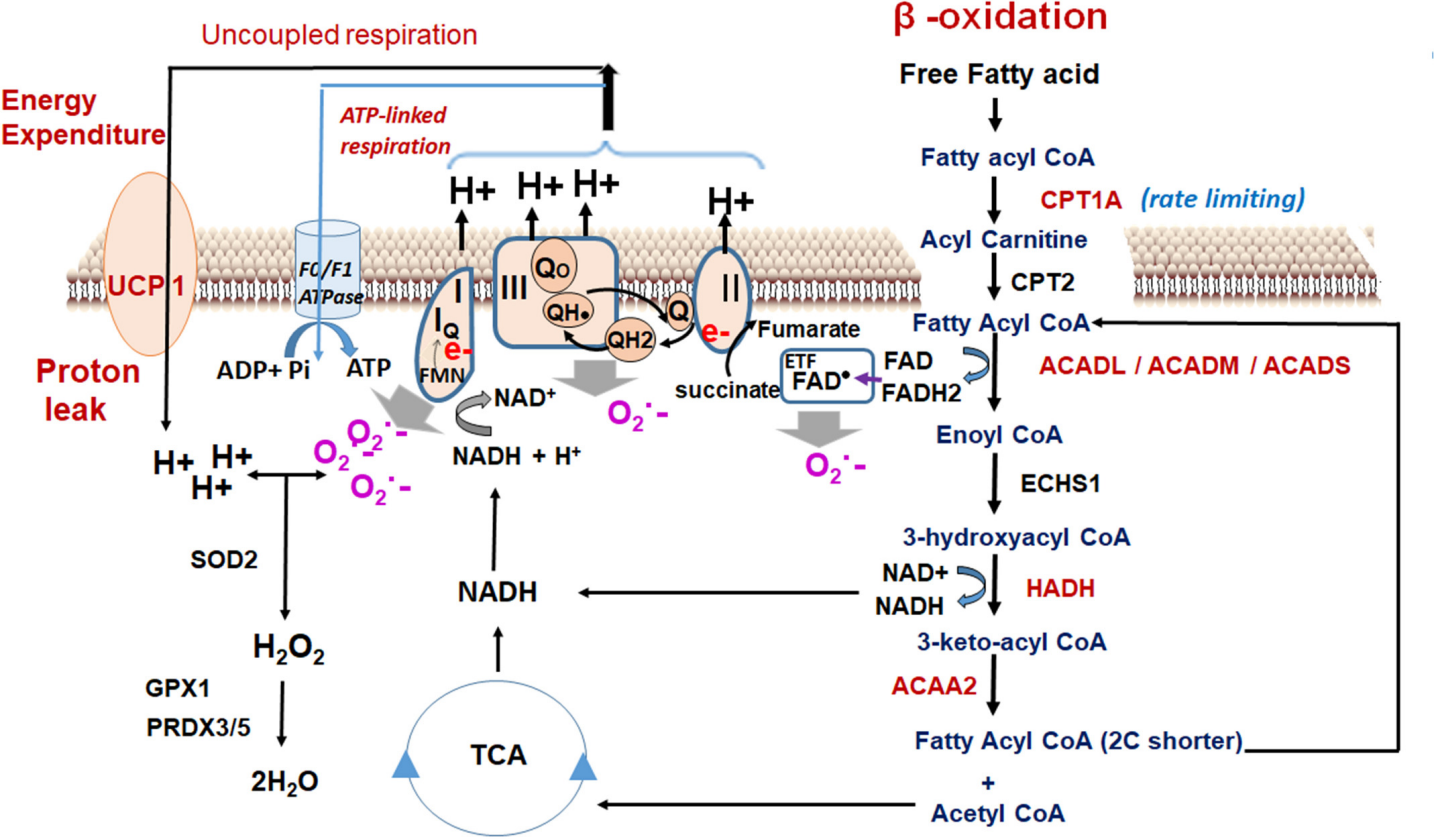
Oxidant quenching cycle drives the proton leak in MS-275-treated adipocytes. The pathway is initiated upon an increase in the β-oxidation flux that results in the superoxide generation. The quenching of the superoxide by antioxidant enzymes at the mitochondrial matrix drives the proton leak and hence the energy expenditure. The enzymes and the pathway that are activated upon MS-275 treatment are marked in red. FAD: Flavin adenine dinucleotide; ETF; Electron- transferring flavoprotein.

Our study revealed that the improvements in the body weight management upon combined treatment of MS-275 and liraglutide in DIO mice could not be replicated alone by either MS-275 or liraglutide. There was a significant reduction in the food intake accounting for the drastic weight loss in the mice receiving the combined therapy. Along with the reduction in calorie intake, we observed significant upregulation of UCP1 in retroperitoneal fat upon liraglutide and MS-275 combined treatment. Contextually, the data presented in this study showed that in addition to UCP1 expression, other factors like enhanced beta-oxidation flux is a key contributor to drive increased calorie expenditure. The real-time respirometry with palmitate as the substrate in cultured adipocytes revealed MS-275-driven enhanced mitochondrial respiration and proton leak that could not be observed upon overnight liraglutide treatment. The in vitro data aligned with our in vivo observation with retroperitoneal WAT where despite the increase in UCP1 upon liraglutide treatment, we did not observe any significant decrease in the adipose mass. The concept is epitomized in the following equation:

(1) ΔE=[UCP1].[Fatty acid].βoxidation flux..........

where ΔE = energy expenditure upon fatty acid oxidation; [UCP1]=UCP1 expression, [Fatty acid]=fatty acid concentration, and β-oxidation flux is the superoxide generator that drives the phenomenon. The data propose that increased beta-oxidation flux and the generated reactive oxidants drives the energy expenditure upon fatty acid oxidation in MS-275-treated cultured adipocytes. ‘The oxidant quenching cycle’ which is initiated with the generation of the superoxides, a by-product of the beta-oxidation pathway, and throttled by the quenching of the superoxides through the enhanced expression and activation of the antioxidant enzymes upon MS-275 treatment, drives the proton leak and the consequent futile cycle causes energy dissipation (Figure 10). The data is in alignment with the concept of mitochondrial ROS-derived thermogenesis as proposed by Spigelman (Chouchani et al., 2017; Chouchani et al., 2016; Chouchani et al., 2019) and Martin Brand (Perevoshchikova et al., 2013) and deserves attention for future exploration. However, the relationship that we proposed between MS-275-driven upregulation of beta-oxidation flux and the energy expenditure is based on our analysis of cultured adipocytes. The lack of in vivo assessment of fatty acid oxidation and energy expenditure is a limitation of our present study. Nonetheless, our in vitro data highlights the significant role of MS-275 in energy expenditure and provides the mechanism that regulates the phenomenon. Moreover, our data aligns with the in vivo observation of MS-275-mediated increased energy expenditure as has been reported in db/db mice (Galmozzi et al., 2013).

MS-275 monotherapy can provide metabolic benefits in rodents (Galmozzi et al., 2013; Ferrari et al., 2017) but has been reported to cause hyperglycemia and hypertriglyceridemia as the dose-limiting toxicities in a few patients suffering from refractory and relapsed acute leukemia (Gojo et al., 2007). In the case of combined therapy of MS-275 and liraglutide, we observed no change in liver weight (Figure 9—figure supplement 1), and there was no fatty liver phenotype in any of the animals receiving liraglutide and MS-275 combined therapy. In the combined treatment group, we observed a 3.13 ± 0.15 fold increase of hepatic IGF-1 (p<0.01, one-way ANOVA, Tukey’s multiple comparison test, Figure 9—figure supplement 2) in the liver tissue that was known to have the anti-inflammatory effect (Hribal et al., 2013). The data indicate that the combined therapy of MS-275 with incretins may ensure an improved metabolic outcome.

Collectively, our results suggest that through Class 1 HDAC inhibition, the functional attributes of the incretin-mediated therapy were significantly enhanced which helped achieve a higher percent normalization of acute fasting blood sugar and a greater reduction in the body weight gain. The adverse effects of non-selective HDAC inhibitors limit their therapeutic applicability beyond cancer therapy (Meier and Wagner, 2014). In this regard, MS-275 being a more selective HDAC inhibitor was better tolerated as monotherapy or when combined with the other forms of cancer therapy in clinical trials for solid tumors and hematological malignancies (Connolly et al., 2017). Despite being relatively mild and manageable at or below the maximally tolerated dose, more study is required to refine the dosing of MS-275 for its application to metabolic diseases. Moreover, combined therapy at the present dosing regimen shows significant advantages in the body weight reduction and glycemic control only upon short-term treatment. In this context, we advocate for a pragmatic approach to select the most suitable partnering incretin, possibly a dual GLP-1R/GIPR agonist such as NNCOO90-2746 (Frias et al., 2017), to achieve the appropriate therapeutic outcome for the long-term management of metabolic diseases like T2D and obesity.

## Materials and methods

Reagents used in this study are described in the following table:

**Key resources table:** 

Reagent type (species) or resource	Designation	Source or reference	Identifiers	Additional information
Cell Line (*Rattus norvegicus*)	BRIN-BD 11 Strain NEDH	ECACC	Cat No: 10033003	A hybrid cell line formed by the electrofusion of a primary culture of NEDH rat pancreatic islets with RINm5F (a cell line derived from a NEDH rat insulinoma)
Transfected construct (conserved CRE sequence; *Homo-sapiens*)	CRE6X-Luc	Kuna et al., 2013	Plasmid vector pcDNA3.1+	A gift from Prof Richard Day, Indiana University
Transfected construct (*Homo-sapiens*)	Β-galactosidase	Kuna et al., 2013	Plasmid vector pcDNA3.1+	
Transfected construct (*Homo-sapiens*)	Rab5A: S34N	Girada et al., 2017	Plasmid vector pcDNA3	EGFP-Rab5A S34N was a gift from Dr. Qing Zhong (Addgene plasmid # 28045);
Transfected construct (*Homo-sapiens*)	GLP-1R_GFP	Kuna et al., 2013	pcDNA3.1+	
Recombinant DNA reagent	pcDNA 3.1+	Invitrogen	Catalog nos. V790-20	
Peptide, recombinant protein	IUB-68	Kuna et al., 2013	Synthesized *in house* (Prof. DiMarchi’s Lab )	Chemically synthesized GIPR agonist
Peptide, recombinant protein	Jant4	Patterson et al., 2011	Synthesized *in house* (Prof. DiMarchi’s Lab )	Chemically synthesized GLP-1R antagonist
Peptide, recombinant protein	Liraglutide (Victoza)	Novo Nordisk		Acylated GLP-1R agonist
A chemical compound, Drug	MS-275	Synthesized *in house* DRILS Chemistry Division		HDAC inhibitor
A chemical compound, Drug	MS-275	Sigma	EPS002	HDAC inhibitor
Commercial assay, kit	Steady lite Plus	Perkin Elmer	6066751	High sensitivity luminescence reporter gene assay system
Commercial assay kit	XF Palmitate Oxidation Stress Test Kit	Agilent	103693–100	Real-time respirometry advanced assay for palmitate-induced oxygen consumption
Commercial assay kit	Seahorse XF Cell Mito Stress Test Kit	Agilent	103015–100	Measures oxygen consumption rate in real-time
Commercial assay kit	Seahorse XF Cell glycolytic Stress Test Kit	Agilent	103344–100	Measures extracellular acidification rate in real-time
Commercial assay, kit	BCA PROTEIN ASSAY KIT	Santa Cruz	sc-200629	Protein estimation
Commercial assay, kit	INSULIN ELISA KIT (RAT/MOUSE)	Merck	EZRMI-13K	Insulin quantification
Commercial assay, kit	cAMP DIRECT-X IMMUNOASSAY ELISA KIT	ARBOR	K019-H1	cAMP immunoassay
Commercial assay, kit	KAPPA 2X SYBR FAST KIT	KAPA Biosystems	KK4601	RT-PCR
Commercial assay, kit	SUPERSCRIPT III cDNA SYNTHESIS KIT	Invitrogen	18080051	First-strand cDNA synthesis
Commercial assay, kit	RNA easy mini kit	Qiagen	74104	
Commercial assay, kit	Plasmid Isolation Kit	Invitrogen	K210005	
Antibody	RPL13A (rabbit polyclonal antibody)	Thermo	PA5-17176	Dilution (1:1000)
Antibody	H3k27 Ac (rabbit polyclonal)	Abcam	Ab4729	Dilution (1:5000)
Antibody	Gαs (mouse monoclonal)	Santa Cruz	Sc-365855	Dilution (1:1000)
Antibody	GLP-1R (mouse monoclonal)	Santa Cruz	Sc-390774	Dilution (1:1000)
Antibody	Β-arrestin ½ (rabbit monoclonal)	CST	CST#4674S	Dilution (1:1000)
Antibody	p44/42 MAPK (Erk1/2) (rabbit polyclonal)	CST	CST#9102	Dilution (1:1000)
Antibody	Β-actin (mouse monoclonal)	Santa Cruz	Sc-47778	Dilution (1:2000)
Antibody	SNAP25 (mouse monoclonal)	Santa Cruz	Sc-376713	Dilution (1:500)
Biological sample (*Rattus norvegicus*)	Pancreatic islets	Vivo Biotech		Freshly isolated from*Rattus norvegicus*
Biological sample (*Mus musculus*)	Visceral adipose tissue, liver, pancreas	In house animal facility at University of Hyderabad, India		Freshly isolated from *Mus musculus*
Software, algorithm	Graphpad Prism 6.0	Prism 6.0		Commercial software

### Methods

#### Animals and treatment

C57BL/6J male mice were purchased from Jeeva Life Sciences, Hyderabad (Cat No: JLS-000664) at 5 weeks of age and were group-housed on a 12:12 hr light-dark cycle at 22°−24°C with free access to standard lab chow diet (Hindustan Liver Ltd, Mumbai, India) and water for 1 week. The animals were then fed ad libitum on a diabetogenic diet, (which is a high-sucrose HFD with 59% kcal from fat *Supplementary material* Table S2) from the th to 24thweek for the assessment of the progression of the diet-induced obesity. At the 10th week, mice were randomized and subjected to pharmacological dosing (six mice/group), with an intraperitoneal injection of vehicle (0.1% DMSO in 0.9% normal saline) or subcutaneous injection of liraglutide (3 mg/kg body weight) twice weekly or intraperitoneal injection of MS-275 (5 mg/kg body weight) every alternate day for a period of 7 weeks until the 17th week. Mono or combinatorial therapy comprising of intraperitoneal injection of MS-275 (5 mg/kg body weight) every alternate day (Monday, Wednesday, and Friday of each week) and subcutaneous injection of liraglutide (3 nmol/kg body weight) twice a week (Tuesday and Saturday) was also initiated at the 10th week and continued until the 17th week. The animals were then subjected to a treatment holiday for a period of 2 weeks after which the therapy was reinitiated for another 4 weeks to assess the metabolic parameters. Figure 8B (i), flow chart, describes the treatment regimen.

For the measurement of the cumulative food intake vis-à-vis body weight gain, C57BL/6J male mice were fed on a HFD for 15 weeks. The mice were then administered the intraperitoneal injection of MS-275 (5 mg kg^−1^ body weight) every alternate day (Monday, Wednesday, and Friday of each week) and subcutaneous injection of liraglutide (3 nmol kg^−1^ body weight) twice a week (Tuesday and Saturday) for a period of 4 weeks during which cumulative food intake and the body weight gain were evaluated every day.

#### Pharmacological and metabolism studies

Acute fasted glucose was assessed on DIO mice that received MS-275 5 mg kg^−1^ body weight and liraglutide 3 nmol kg^−1^ body weight. Twenty-four hour after the first dose the animals received a second dose of the same drugs and fasted for 5 hr following which blood glucose was evaluated. Chronic fasted blood glucose was assessed at the 24thweek following 5 h of fasting. A glucose tolerance test was performed on the 24th week when HFD fed mice were fasted for 5 hr and subjected to intraperitoneal injection of glucose (2 g/kg body weight) (D-glucose [Sigma] 20% w/v in 0.9% normal saline). Blood glucose level from the tail vein was measured using a Roche-Accu Check glucometer just before injection, and 15, 30, 60, 90, and 120 min after injection. For measurement of visceral WAT, animals were sacrificed at the end of the experiment and retroperitoneal, mesenteric, and epididymal fat pads along with liver and blood samples were collected from individual animals and stored at −80°C for further processing.

#### Isolation and culture of rat islets

Pancreatic islets were isolated from Sprague Dawley rat at Vivo Biotech Hyderabad as approved by Institutional Animal Ethical Committee (approval No: VB/IAEC/04/2016/144/Rat/SD) following the protocol of Carter et al., 2009 with modifications. Briefly, the animals were euthanized under anaesthesia after which abdominal incision was performed aseptically and an enzyme solution of Collagenase P (Sigma # Cat No: 11213857001) was injected through the common bile duct below the bifurcation to distend the pancreas. The pancreas was subsequently harvested and digested by an oxygenated enzyme solution comprising of 1 mg/mL collagenase P for 30 min, and the digestion was stopped by an ice-cold quenching-buffer consisting of 10% FBS in HBSS. The islets were dissociated through mechanical shaking and pelleted through a brief spin at 1000 rpm for 2 min. Dissociated islets were further purified through ficoll gradient and cultured in RPMI 1640 containing 2 mM L-Glutamine, 10% FBS, and 1% pen strep at a density of 100 IEQ (islet equivalent)/mL. Experiments were carried out within 72h of initiation of the culture.

All animal studies were approved by and performed according to the guidelines of the Institutional Animal Ethics Committee of the University of Hyderabad, (Approval No: IAEC/UH/151/2017/PPB/P13); Vivo biotech (VB/IAEC/04/2016/144/Rat/SD), and the National Institute of Nutrition (Approval No: P23F/IAEC/NIN/11/2017/PM/C57BL6/J-260(M)).

#### Cell culture

BRIN-BD11 pancreatic beta cells were purchased from the European Collection of Authenticated Cell Cultures cat.no: 10033003. The cell lines have been tested for insulin expression and Glucose-stimulated Insulin secretion. The cells do not have mycoplasma contamination.

BRIN-BD11 pancreatic beta cells (ECACC cat.no: 10033003) were cultured at 37°C with 5% CO_2_ in RPMI medium 1640 GLUTAMAX supplemented with 10% heat-inactivated Fetal Bovine Serum (FBS), 1 mM Sodium pyruvate, 50 µM β-mercaptoethanol, 10 µg/mL gentamycin, 100 units/mL penicillin and 100 µg/mL streptomycin following the protocol previously published from this laboratory (Kuna et al., 2013).

#### Screening of compounds

BRIN-BD11 pancreatic beta cells were transfected with a cAMP-responsive luciferase reported plasmid and beta-galactosidase plasmid at a 1:1 ratio (3.5 µg:3.5 µg) in a 70 mm culture dish following which the cells were seeded into a 96-well plate at a density of 30,000 cells per well. After adherence, the cells were treated with small molecules at a concentration of 10 μM for 18 hr. Cells were then incubated with liraglutide (100 nM) for 4 hr and cAMP generation was evaluated using a multimerized cAMP reporter element luciferase reporter assay.

### Time-course assessment of GLP-1R-mediated cAMP generation

A time-course assessment of GLP-1R-mediated cAMP generation was conducted in BRIN-BD11 pancreatic beta cells using liraglutide (100 nM) and a direct cAMP enzyme immunoassay kit (Enzo Direct cAMP ELISA kit ADI 900066). The cells were seeded at a density of 100,000 cells / well in 24-well plates and after adherence was treated with MS-275 (5 µM) for 16 h. Following incubation with Krebs Ringer’s Buffer (KRB) containing 0.2% BSA and 1.1 mM glucose for 1h, liraglutide (100 nM) was added in KRB media containing IBMX (200 µM) and 5.5 mM glucose. Five-minutes post-treatment, the excess ligand was washed by (Krebs Ringer buffer KRB) and the cAMP generation was determined 5, 15, 30, and 90 min after KRB wash by direct immunoassay following the assay kit procedure.

### CRE-luciferase assay

GLP-1R-mediated signaling was assessed by a multimerized cAMP-responsive element (CRE) luciferase reporter assay, following the method as previously described from this laboratory (Kuna et al., 2013; Asalla et al., 2016 and Girada et al., 2017). The cells were grown in a 70 mm dish until they attain 70% confluence. A cAMP-responsive element-luciferase reporter plasmid encoding the luciferase reporter gene under the control of the minimal promoter and six tandem repeats of the CRE transcriptional response element (CRE 6X-Luc) and a beta-galactosidase plasmid were transfected transiently in 1:1 ratio using lipofectamine 2000, following manufacturer’s instructions. Four hours after transfection, cells were transferred to 96-well Cell Bind plates (Corning) at a density of 30,000 cells per well and treated with or without MS-275 (5 µM) after adherence. After 24 h, the media was removed and cells were treated with incretin receptor agonist in complete medium for another 4h. The medium was then aspirated, cells were lysed, and luciferase activity was measured using steady-lite plus reagent (Perkin Elmer Life and Analytical Science, Waltham, MA). Correction for inter-well variability in transfection was determined by b-galactosidase assay through the addition of 2-nitrophenyl-beta galactopyranoside (Sigma). After incubation for 15 min at 37°C, substrate cleavage was quantified by measuring optical density at 405 nm in an ELISA plate reader (Perkin Elmer, USA) and the corresponding values were used to normalize luciferase activity. The data are expressed as fold change in luciferase activity upon incretin agonist treatment relative to untreated control (basal level).

For Rab5A-S34 N transfection and subsequent cAMP assessment, BRIN-BD11 pancreatic beta cells were transfected with Rab5A S34N mutant, CRE6X-Luc and beta-galactosidase plasmid in 1:1:1 (2.5 µg:2.5 µg:2.5 µg) in 70 mm dish and 24 h post-transfection, assayed for GLP-1R-mediated cAMP generation . . The data is presented as a four-parameter logistic curve analyzed in Graphpad Prism (version 6.0), and each data point is assessed in duplicates. The dose-response curve represents the mean SEM of three independent experiments.

### Glucose uptake assay

Glucose uptake assay was carried out through direct incubation of cultured pancreatic beta cells with a fluorescent D- glucose analog 2-[N-(7-nitrobenz-2-oxa-1,3diazol-4-yl) amino]−2-deoxy-D glucose (NBDG) following the method of Zou *et.al.*(Zou et al., 2005). Briefly, BRIN-BD11 pancreatic beta cells were seeded at a density of 50,000 cells per well in 24-well plate and 24 hr post-seeding were treated with or without MS-275 (5 μM) for16h. The cells were then washed with Krebs-Ringer bicarbonate (KRB) buffer [115 mM NaCl, 4.7 mM KCl, 1.28 mM CaCl2, 1.2 mM KH2P04, 1.2 mM MgSO4, 10 mM NaHCO3, 0.1% (wt./vol) BSA, pH 7.4] and incubated with 300 µL of the same buffer containing 0.1% BSA, without any supplemented glucose for 60 min. The NBDG was added to KRB for 20 min after which the incubation medium was removed, cells washed with ice-cold PBS, and lysed with the lysis buffer containing 0.1% Triton X 100 in dark. The lysates were transferred to the Corning 96 well polystyrene Black microplate and read in triplicate in Victor3 microplate reader at an excitation/emission = 485/535 nm.

### Insulin secretion

1. Cultured pancreatic beta cells: Insulin secretion studies from BRIN-BD11 cells were conducted using a Millipore Rat/Mouse Insulin ELISA kit (cat no. EZRMI-13K) as described by Asalla et al., 2016. In brief, the cells were seeded in 24-multi-well plates at a density of 100,000 cells/well. MS-275 (5 µM, dissolved in 0.1%DMSO) was added after cells have adhered and cultured for 16h in complete medium. Before the insulin release experiment, the cells were washed with KRB buffer and pre-treated with 300 µL of the same buffer containing 0.1% w/v BSA, without any supplemented glucose for 60 min. Insulin secretion was measured in the presence of varying concentrations of glucose following the addition of the GLP-1R agonist liraglutide (0.1 nM) for 30 min. At the end of the stimulation, the medium was collected and cleared by centrifugation. The cell lysates were quantified for protein concentration to normalize the insulin secretion results. Ten microliters of cell supernatant were used for the ELISA. The insulin was measured as ng/mg of protein and expressed as fold increase relative to basal insulin secretion.
2. Upon Bafilomycin treatment: During the pre-treatment of BRIN-BD11 pancreatic beta cells, Bafilomycin A1 (100 nM) was added for 30 mins following which liraglutide (1 nM) was added to the incubation media for 30 min and insulin secretion from cell supernatant was measured as described before. Protein content was determined using the BCA kit (Santa Cruz).
3. Upon Rab5A S34N transfection: BRIN-BD11 pancreatic beta cells were seeded at a density of 100,000 cells/well following transfection with Rab5A-S34N plasmid or empty vector in 24-well tissue culture-treated plates. After the adherence of cells, they were treated with MS-275 (5 µM) for 16h. The cells were then washed with KRB buffer and incubated for 60 min with KRB Buffer containing 0.1% w/v BSA, without any supplemented glucose. The cells were then treated with or without liraglutide (1 nM) for 30 min and insulin secretion from cell-supernatant was assessed as described before. Protein content was determined using the BCA kit (Santa Cruz).
4. Rat islets: Insulin secretion from rat islets was conducted following the method of Llanos et al., 2015. Briefly, 20 islet equivalents (iev) per well were seeded in 24-well plates in RPMI1640 GLUTAMAX medium supplemented with 10% FBS and 1% pen strep, with and without MS-275 (5 µM). After 16h, ievs were incubated in 300 LKRB buffer with 0.2% BSA for 1h, without D-glucose. Before the agonist treatment, the buffer was removed and fresh 300 µL KRB buffer containing 0.2% BSA and D-Glucose at a specified concentration was added to the ievs. The ievs were treated with the GLP-1R agonist liraglutide (1 nM) for 30 min and the supernatant was collected. The islets were lysed with 0.1N HCl and assayed for protein concentration using the BCA kit (Santa Cruz Biotechnology). The supernatant collected was assayed for insulin secretion with a rat/mouse insulin ELISA kit (Merck Millipore)

### Real-time respirometry

#### Pancreatic beta cells

BRIN-BD11 cells were seeded in an XFe 24 cell culture microplate at 20,000 cells/well in 500 μL of complete medium and incubated overnight at 37°C in a CO_2_ incubator, with or without the pre-treatment with MS-275 (5 µM). The cells were washed with the assay medium comprising 114 mM NaCl, 4.7 mM KCl, 1.2 mM KH2PO4, 1.16 mM MgSO4, 20 mM HEPES pH 7.4, 2.5 mM CaCl2 supplemented with 10 mM glucose. Following wash with the assay medium, the cells were incubated for 60 min at 37°C in air. Plates were transferred to a Seahorse Bioscience XFe24 extracellular flux analyzer (controlled at 37°C) and subjected to an equilibration period. To inject Oligomycin, FCCP and Rotenone/Antimycin (Mito stress kit constituents) a constant concentration/variable loading strategy as per the manufacturers’ protocol was followed. The concentration of Oligomycin and FCCP was determined through titration. One assay cycle comprised of a 1 min mix, 2 min wait, and 3 min measurement period. After measuring basal OCR for three cycles, Oligomycin (1 µM) was added to inhibit ATP synthase and thus determine the proportion of respiration used to generate ATP synthesis. After four further assay cycles, carbonyl cyanide-4-(trifluoromethoxy) phenyl hydrazone FCCP (1 µM) was added to determine maximal respiration by mitochondria by uncoupling ATP synthesis from electron transport. After another four assay cycle, Rotenone (0.5 µM) plus Antimycin A (0.5 µM) was added to measure the non-mitochondrial respiratory rate. Three determinations were made for basal and each inhibitor injection. The extracellular acidification rate (ECAR) was simultaneously measured to OCR. Data are expressed as means ± S.E of three independent assessed wells measured in triplicate.

To assess ECAR (glycolytic flux), the XF Glycolysis Stress Test assay was used. To perform each assay a 24-well plate was used where BRIN-BD11 pancreatic beta cells were seeded at a density of 20,000 cells per well in RPMI 1640 GlutaMax medium with and without MS-275 at 37°C in the 5% CO_2_ incubator for 16h. The medium was changed to XF basal medium with added glutamine before the initiation of the experiment. The injection consisted of glucose, Oligomycin, and 2-DG at a concentration of 10 mM, 1 µM, and 50 mM, respectively. After the addition of these reagents, the cartridge was hydrated for 1h in the non-CO_2_ incubator, and -post-incubation was assessed in the XF-24 flux analyzer in the same manner as mentioned in OCR.

#### Adipocytes

3T3 L1 adipocytes at the 7th day of differentiation were subjected to real-time respirometry in XFe 24 analyzer (Seahorse) using XF Palmitate-BSA FAO Substrate following the kit protocols with modifications. The assay medium contains 2.5 mM glucose, 0.5 mM L-carnitine, and the experiment was initiated with the addition of Palmitate-BSA conjugate (500 μM) or the BSA control. Of the four injection ports, port A is loaded with liraglutide, port B: Oligomycin, port C: FCCP; port D: Rotenone/Antimycin final well concentration being 1 μM, 5 μM, and 8 μM, respectively. The period of the experiment is 180 min; liraglutide from port A is added 30 min after initiation of the assay and oligomycin added 90 min after liraglutide injection.

### Palmitate survival assay

BRIN-BD11 cultured pancreatic beta cells were seeded at a density of 10,000 cells/well in a 96-well tissue-culture-treated plate. Post-adherence cells were treated with BSA (control) and Palmitate Conjugate (200, 150, and 100 µM) in the presence or absence of MS-275 (5 µM). The cells were incubated for 24 hr and media was removed. Fresh media (100 µL) was added and 10 µL of 5 mg/mL of MTT (USB chemicals) in 1X PBS was added to each well in dark and incubated for 4 hr at 37°C and 5% CO_2_. After that media was removed and 100 µL of DMSO was added to each well and kept for shaking at 300 rpm on a shaker for 15 min. The colorimetric readout was taken at 570 nm in a multimode plate reader and data was analyzed in Graphpad prism 6.0.

### Measurement of ROS

Cultured pancreatic beta cells were seeded in a tissue-culture-treated 24-well plate at a seeding density of 100,000 cells/well. Following adherence and attainment of morphology, the cells were treated with BSA (control) or palmitate (100 µM and 150 µM) in the presence or absence of MS-275 (5 µM) for 24 hr at 37°C. The media was discarded and cells were then washed with 1X PBS and 10 µM of carboxy-H_2_DCFDA was added to the cells and incubated at 37°C for 30 min. Post-incubation, cells were quickly washed twice with ice-cold PBS and then lysed with 0.1% Triton-X 100 in dark. The lysates were mixed and transferred to a black colored 96-well plate and fluorescence was assessed at excitation/emission at 485/530 nm and protein quantified for normalization.

### RNA sequencing

RNA seq data sets have been deposited in GEO (https://www.ncbi.nlm.nih.gov/geo/query/acc.cgi?acc=GSE139147).

The total RNA (150 ng) was used for preparing libraries for RNA sequencing. The RNA was fragmented and then converted to cDNA according to the kit protocol (NEB #E7770S). The cDNA was end-repaired and further purified using AMPure XP beads (A63880 AMPure XP). The cleaned cDNA was adapter-ligated, purified, and subjected to 12 PCR cycles of amplification using primers provided in the kit (NEB #E7770S). The PCR products were purified using AMPure XP beads. The quantification and size distribution of the prepared library was accomplished using Qubit fluorimeter and Agilent Tape station D1000 Kit (Agilent Technologies) following the manufacturer’s instructions.

### RNA SEQ data analysis

The transcriptome libraries constructed using the NEB adapters were sequenced on Illumina HiSeq at 150 nucleotide read length using the paired-end chemistry. The raw reads were subjected to contamination [structural RNA/low complexity sequences, adapters] removal by mapping with *bowtie* 2–2.2.1. The data set after contamination removal was mapped to the Rattus_norvegicus.Rnor_6.0 using STAR. Reads mapping to genes Rattus_norvegicus.Rnor_6.0 gene list [GTF] were counted using the *feature count* module of *sub reads* package and were normalized in DESeq2-3.5 followed by differential expression analysis.

### Differential gene expression analysis

Differentially expressed genes were selected based on log_2_-ratio change with p-value<0.05 (Student‘s t-test, unpaired). Hierarchical clustering was performed with the programs Cluster (uncentered correlation; average linkage clustering) and Treeview (Eisen et al., 1998).

### Biological interpretation of RNA seq data

#### Gene ontology (GO) annotation

The GO annotation was carried out using the Gorilla web server [http://cbl-gorilla.cs.technion.ac.il]. Term enrichment of differentially regulated genes was calculated based on the rat genome database as a background. GO terms with corrected p<0.05 represents significant enrichment.

#### Gene set enrichment analysis (GSEA)

We carried out a GSEA analysis where all genes were ranked based on their expression ratios (enrichment score). The gene sets with a p-value<0.05 and an FDR value <0.25 were considered to be significantly affected. For the GSEA analyses, we have used the gene sets from various sources: including GO, Biocarta, (http://www.biocarta.com/), KEGG, and Reactome.

#### String analysis

The interactome analysis corresponding to the selected DEGs was retrieved from the STRING database (Franceschini et al., 2013). Genes in the interaction network are represented with nodes, while the interactions between two genes are represented with edges. The selection of the hub gene is based on the score of the nodes, which is calculated by the count of edges launching from a gene in the network.

### Real-time PCR

Total RNA was extracted from BRIN-BD11 cells, cultured human adipocytes, and mouse adipose tissues using TriZol reagent (Life Technologies), and 1 µg of total RNA was used to synthesize cDNA using Superscript III First-Strand cDNA synthesis kit. Specific mRNA was amplified and quantified by quantitative real-time PCR using Quant Studio 5 (A and B Biosystems). Kapa sybr fast universal master mix (Kapa Biosystems) reagent was used to assess the relative abundance of the mRNAs measured using the 2^-ΔΔC^_T_ method (Livak and Schmittgen, 2001). Data were normalized using 18s rRNA as an invariant reference in adipocytes and GAPDH in cultured pancreatic beta cells. Primers used for amplification are listed in Supplementary file 3 Table 3.

### Western blotting

BRIN-BD11 cells were treated with MS-275 (5 µM) overnight in complete medium and were lysed in cell lysis buffer containing protease inhibitor (Sigma) and phosphatase inhibitor (EMD Millipore). Fifteen micrograms of the cell extracts were subjected to western blot analysis using Gα_s_ mouse monoclonal antibody (Cat. No: sc-135914) following standard procedures. ERK (Cat No: CST#9102) immunoblot was used as a loading control. In the case of SNAP 25, immunoblot was carried out using the SNAP 25 antibody (Cat No: SC-376713); the RPL 13a antibody (Cat No: PA5-17176) was used as a loading control. The images are quantified using Image J and expressed as intensity (arbitrary units) measured as the ratio of the protein expression and the loading control.

### Confocal microscopy

The GLP-1 receptor-ligand internalization was captured following the method of Kuna *et.al* (Kuna et al., 2013). Briefly, BRIN-BD11cells were transfected with GLP-1R-GFP using lipofectamine 2000 and plated in six-well plates containing 25 mm diameter glass coverslips (Fisher Scientific). Forty-eight hours later cells were treated with and without MS-275 for 16 h. Later, cells on coverslips were incubated with liraglutide (100 nM) in 200 μL of Krebs-HEPES buffer for 60 min at 4°C in the dark. Cells were then washed in phosphate buffer saline (PBS) and incubated at 37°C for the desired time in complete medium, after which they were fixed in 3% paraformaldehyde, mounted in Vectashield mounting medium (Vector Laboratories), and imaged using a Zeiss LSM 510-META confocal laser scanning microscope (Carl Zeiss, Oberkochen, Germany) equipped with krypton-argon laser sources. Pinhole diameter was maintained at one airy unit. Image acquisition was conducted using a 63X oil immersion objective lens with a 2X optical zoom with the Zenlite 2011 program.

### MS-275 ^1^H NMR data

^1^H NMR (400 MHz, DMSO-d6) of MS-275 (*synthesized in house*) is as follows: δ 9.62 (brs, 1H), 8.59 (s, 1H), 8.53(dd, 1H, *J* = 4.8, 1.2 Hz), 7.97 (t, 1H), 7.92 (d, 2H, *J* = 8.0 Hz), 7.78 (d, 1H, *J* = 8.0 Hz), 7.41 (dd, 1H, *J* = 7.2, 4.8 Hz), 7.36 (d, 2H, *J* = 8.2 Hz), 7.16 (d, 1H, *J* = 7.2 Hz), 6.96 (m, 1H), 6.78 (dd, 1H, *J* = 8.4, 1.2 Hz), 6.58 (m, 1H), 5.09 (s, 2H), 4.89 (s, 2H), 4.28 (d, 2H, *J* = 6.0 Hz); HPLC: 99.67%; Mass: *m/z* = 377.10 [M+H]^+^.

### Statistical analysis

Statistical analysis was performed using Graph Pad Prism 6.0. The data are presented as means ± SEM. The analysis of the results obtained in the in vivo experiments was assessed by one-way ANOVA (Tukey’s multiple comparison test). For IPGTT and body weight gain, two-way ANOVA was used to assess the statistical significance of difference among groups (p<0.05). For cell-based assays, statistical significance was assessed by one-way ANOVA, Welch’s t-test, and Student’s t-test (unpaired).

## Data Availability

RNA seq data sets have been deposited in GEO (https://www.ncbi.nlm.nih.gov/geo/query/acc.cgi?acc=GSE139147). The following dataset was generated: MitraPBeleSMuthaNVKatikaMR2019BRIN-BD11 pancreatic beta cell mRNA profile upon treatment with Class1 HDAC inhibitor MS-275NCBI Gene Expression OmnibusGSE139147

## Additional files

Supplementary file 1. Table 1: Primary hits in small molecule screening library at a concentration of 10 µM. BRIN-BD11 pancreatic beta cells were treated for 18 hr to assess its effect on GLP-1R (liraglutide)-mediated cAMP generation as measured by Cre-Luciferase reporter assay.

Supplementary file 2. Table 2: Composition of high-sucrose high-fat diet fed to C57BL/6 male mice.

Supplementary file 3. Table 3: Sequences of primers for quantitative RT-PCR as described in the table.

Transparent reporting form
