# Supplementary material for: MS-275, a class 1 histone deacetylase inhibitor augments glucagon-like peptide-1 receptor agonism to improve glycemic control and reduce obesity in diet-induced obese mice
Source: eLife. 2020 Dec 22;9:e52212. doi: 10.7554/eLife.52212 (PMC7755393; doi:10.7554/eLife.52212)
Supplement: Figure 2—source data 1. — Western blot pictures (uncut) showing the impact of MS-275 on H3K27 acetylation; RPL-13a immunoblot served as the loading control. [file elife-52212-fig2-data1.docx]

**
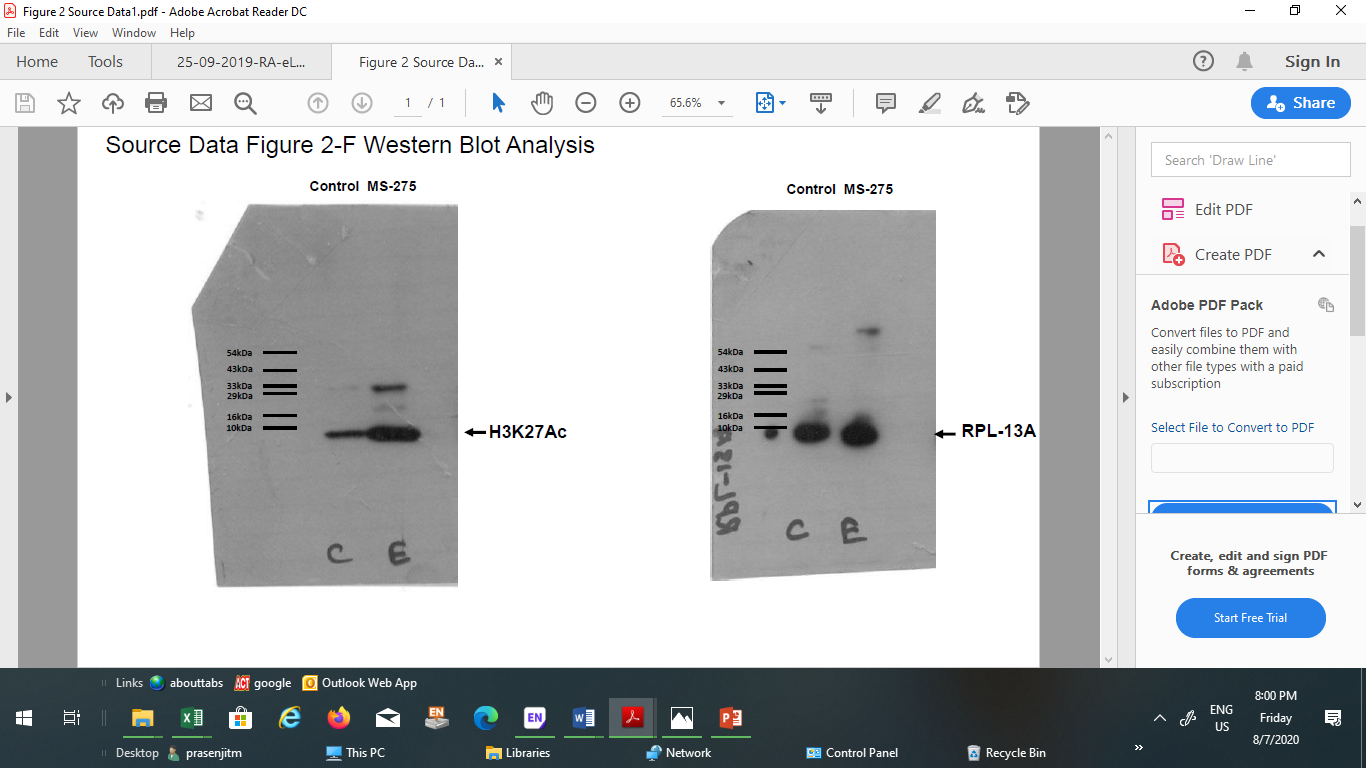
Figure 2 –Source Data 1**

**Source Data Fig 2G:** Western blot pictures (uncut) showing the impact of MS-275 on H3K27 acetylation; RPL-13a immunoblot served as the loading control.
