# Supplementary material for: MS-275, a class 1 histone deacetylase inhibitor augments glucagon-like peptide-1 receptor agonism to improve glycemic control and reduce obesity in diet-induced obese mice
Source: eLife. 2020 Dec 22;9:e52212. doi: 10.7554/eLife.52212 (PMC7755393; doi:10.7554/eLife.52212)
Supplement: Figure 3—source data 1. — Western blot pictures (uncut) showing the impact of MS-275 on Gαs protein expression; ERK immunoblot was considered as the loading control. Source Data Figure 3B: Source Data Figure 3C: Source Data Figure 3D and Figure 3E: Source Data Figure 3F: Source Data Figure 3G: Source Data Figure 3H: Source Data Figure 3K: Source Data Figure 3. [file elife-52212-fig3-data1.docx]

**Figure 3 Source Data 1:**

**Source Data Fig 3A.**

**
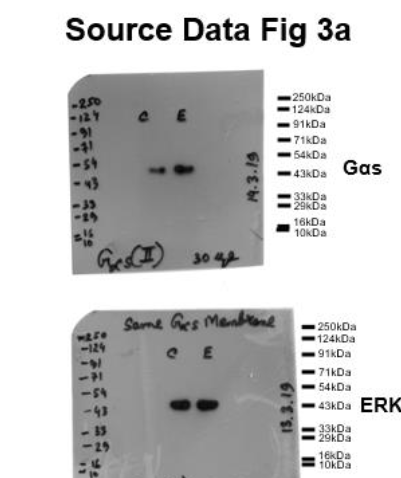
**

**Figure legend: Source Data Fig 3A:** Western blot pictures (uncut) showing the impact of MS-275 on Gαs protein expression; ERK immunoblot was considered as the loading control.
