# Supplementary material for: MS-275, a class 1 histone deacetylase inhibitor augments glucagon-like peptide-1 receptor agonism to improve glycemic control and reduce obesity in diet-induced obese mice
Source: eLife. 2020 Dec 22;9:e52212. doi: 10.7554/eLife.52212 (PMC7755393; doi:10.7554/eLife.52212)
Supplement: Figure 3—source data 4. [file elife-52212-fig3-data4.docx]

**Figure 3 Source Data 4:**

**
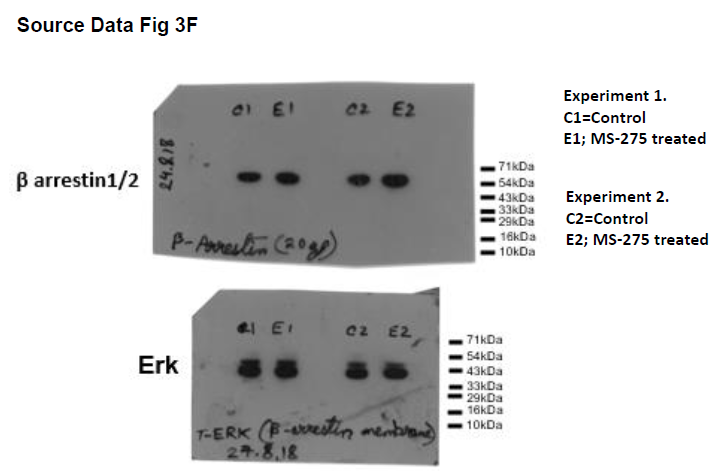
**

**Figure 3 Source Data 4: Source Data 3 F:** Western blot pictures (uncut) showing the impact of MS-275 on beta-arrestin protein expression; ERK immunoblot was considered as the loading control

.
