# Supplementary material for: MS-275, a class 1 histone deacetylase inhibitor augments glucagon-like peptide-1 receptor agonism to improve glycemic control and reduce obesity in diet-induced obese mice
Source: eLife. 2020 Dec 22;9:e52212. doi: 10.7554/eLife.52212 (PMC7755393; doi:10.7554/eLife.52212)
Supplement: Figure 4—source data 2. [file elife-52212-fig4-data2.docx]

**Figure 4 -Source Data 2**


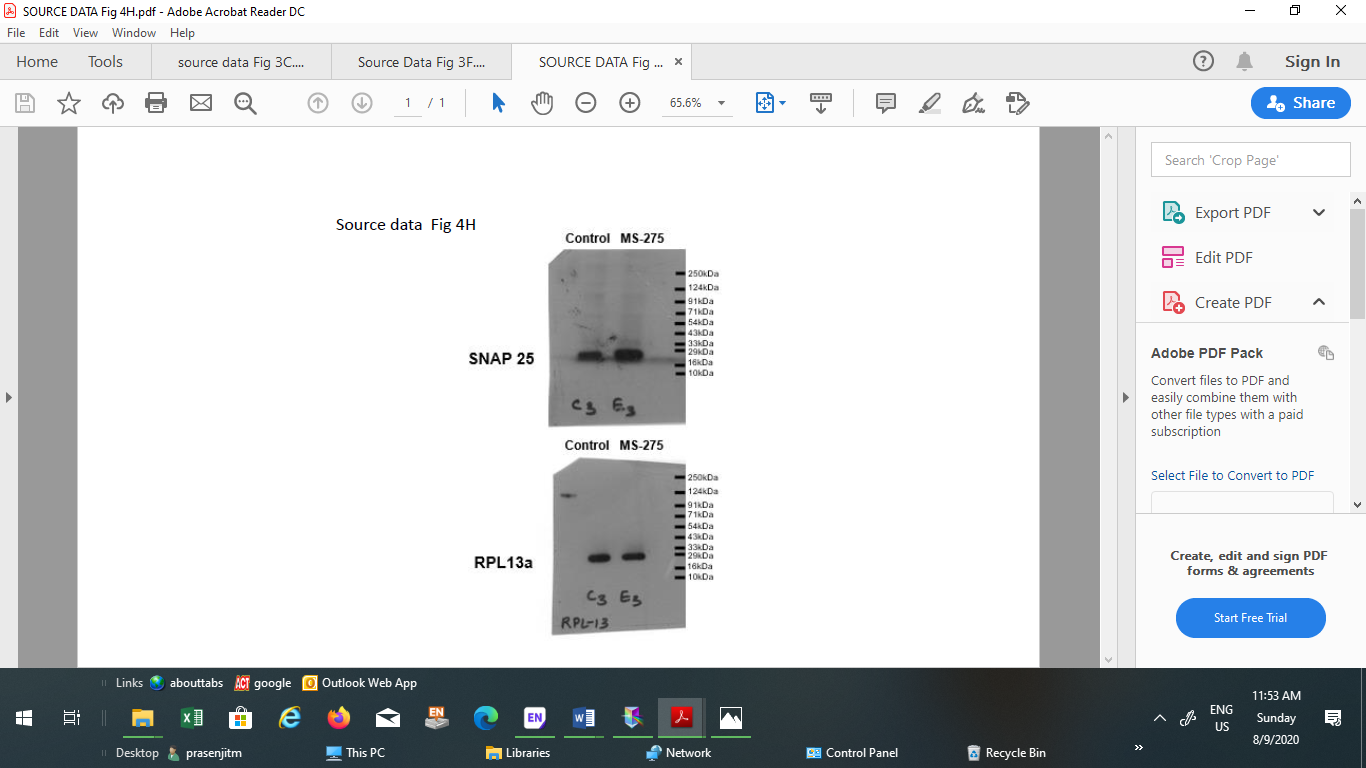


**Figure 4 Source Data 2: Source Data Figure 4H:** Western blot pictures (uncut) showing the impact of MS-275 on SNAP 25 protein expression in cultured pancreatic beta cells; RPL-13a immunoblot was considered as the loading control
