# Supplementary material for: MS-275, a class 1 histone deacetylase inhibitor augments glucagon-like peptide-1 receptor agonism to improve glycemic control and reduce obesity in diet-induced obese mice
Source: eLife. 2020 Dec 22;9:e52212. doi: 10.7554/eLife.52212 (PMC7755393; doi:10.7554/eLife.52212)
Supplement: Figure 7—source data 2. — Western blot pictures (uncut) showing the impact of the vehicle, liraglutide, MS-275, and combined liraglutide and MS-275 co-therapy on GLP-1R protein expression in pancreatic tissue pooled from each group; beta-actin immunoblot served as the loading control. b. Source Data Figure 7G: Western blot pictures (uncut) showing the impact of the vehicle, liraglutide, MS-275, and combined liraglutide and MS-275 co-therapy on Gαs protein expression in pancreatic tissue pooled from each group. Beta-actin immunoblot served as the loading control. [file elife-52212-fig7-data2.docx]

**Figure 7 Source Data 2:**

**a. Source Data Fig 7F**

**GLP-1R**

**B
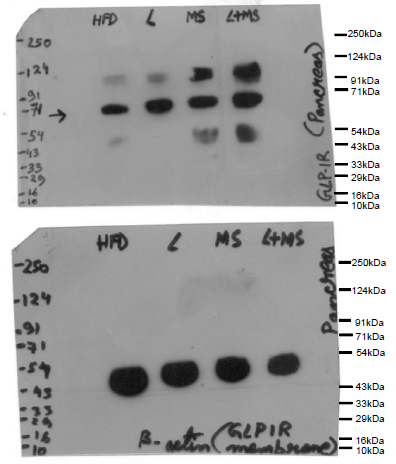
eta**

**Actin**

**Figure 7 Source Data 2: Source Data Fig 7F:** Western blot pictures (uncut) showing the impact of the vehicle, Liraglutide, MS-275, and combined Liraglutide and MS-275 co-therapy on GLP-1R protein expression in pancreatic tissue pooled from each group; Beta-actin immunoblot served as the loading control.

**b. Source Data Figure 7G:** l.

**
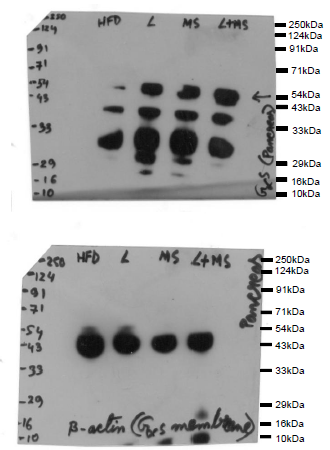
**

**Gαs**

**Β-actin**

**Figure 7 Source Data 2: Source Data Fig 7G:** Western blot pictures (uncut) showing the impact of the vehicle, Liraglutide, MS-275, and combined Liraglutide and MS-275 co-therapy on Gαs protein expression in pancreatic tissue pooled from each group. Beta-actin immunoblot served as the loading control.
