# Supplementary material for: MS-275, a class 1 histone deacetylase inhibitor augments glucagon-like peptide-1 receptor agonism to improve glycemic control and reduce obesity in diet-induced obese mice
Source: eLife. 2020 Dec 22;9:e52212. doi: 10.7554/eLife.52212 (PMC7755393; doi:10.7554/eLife.52212)
Supplement: Supplementary file 1. — BRIN-BD11 pancreatic beta cells were treated for 18 hr to assess its effect on GLP-1R (liraglutide)-mediated cAMP generation as measured by Cre-Luciferase reporter assay. [file elife-52212-supp1.docx]

**Supplementary File 1**

**Supplementary File 1: Table1**

| **Compound** | **Structure** | **Nature** | **Liraglutide treatment** | **cAMP generation (fold change)** | **St. Dev** |
| --- | --- | --- | --- | --- | --- |
| PBS |  | Vehicle control | - | **1** | **0.2** |
|  |  |  | + | **8.2** | **2.8** |
| MS-275 | 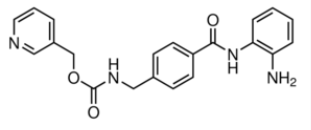 | Class1 HDAC inhibitor | - | **3.7** | **0.1** |
|  |  |  | + | **31.7** | **3.1** |
| Trichostatin A | 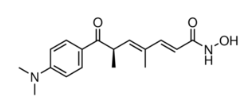 | Class1 HDAC inhibitor | - | **1.9** | **0.1** |
|  |  |  | + | **15.2** | **0.6** |
| SAHA | 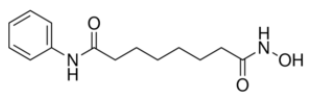 | Class1 HDAC inhibitor | - | **1.3** | **0.1** |
|  |  |  | + | **17.8** | **0.1** |
| AR-42 | 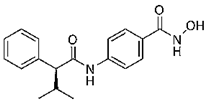 | Class1 HDAC inhibitor | - | **1.5** | **0.1** |
|  |  |  | + | **23.6** | **1.4** |
| Valproic acid | 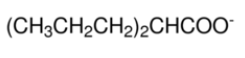 | Class1 HDAC inhibitor | - | **0.6** | **0.2** |
|  |  |  | + | **7.0** | **0.3** |

**Supplementary File 1: Table1 legend:**

Primary hits in small molecule screening library at a concentration of 10µM. BRIN-BD11 pancreatic beta cells treated for 18 hours to assess its effect on GLP-1R (liraglutide) mediated cAMP generation. Results are reported as fold-increase over vehicle control in a cAMP-responsive luciferase reporter assay. Data represented as mean ±SD (n=2 replicates per treatment).
