# Supplementary material for: MS-275, a class 1 histone deacetylase inhibitor augments glucagon-like peptide-1 receptor agonism to improve glycemic control and reduce obesity in diet-induced obese mice
Source: eLife. 2020 Dec 22;9:e52212. doi: 10.7554/eLife.52212 (PMC7755393; doi:10.7554/eLife.52212)
Supplement: Supplementary file 2. [file elife-52212-supp2.docx]

**Supplementary File 2: Table 2: High Sucrose High Fat Diet**

| **Composition** | **Gram%** | **Kcal%** |  |
| --- | --- | --- | --- |
| Protein | 25.375 | 19.88 |  |
| Fat | 33.6 | 59.0 |  |
| Carbohydrate | 26.95 | 21.12 |  |
| Total |  | 100 |  |
| **Ingredients** | **Gram** | **kcal** | **Gram/kg of diet** |
| Casein 30 mesh | 200 | 800 | 250 |
| L-Cysteine | 3 | 12 | 3.75 |
| Lard | 250 | 2250.74 | 312.5 |
| Soybean oil | 19.4 | 225 | 24.25 |
| Sucrose | 185.6 | 742.18 | 232 |
| Corn starch | 30 | 119.91 | 37.5 |
| Maltodextrin 10 | 0 | 0 | 0 |
| Cellulose BW200 | 50 | 0 | 62.5 |
| AIN Mineral mix | 13 | 0 | 16.25 |
| Di Calcium phosphate | 13 | 0 | 16.25 |
| Calcium Carbonate | 5.5 | 0 | 6.875 |
| Potassium Citrate1H20 | 16.5 | 0 | 20.625 |
| AIN Vitamin Mix | 12 | 40 | 15 |
| Choline Bitartrate | 2 | 0 | 2.5 |
| **Total** | 800 gram |  | 1000 gram |

**Supplementary File 2: Table 2:**

Composition of high sucrose high fat diet fed to C57BL/6 mice. Typical analysis of cholesterol in lard equals 72mg/100 grams. Cholesterol mg/kg of diet equals 225mg.
