# Supplementary material for: MS-275, a class 1 histone deacetylase inhibitor augments glucagon-like peptide-1 receptor agonism to improve glycemic control and reduce obesity in diet-induced obese mice
Source: eLife. 2020 Dec 22;9:e52212. doi: 10.7554/eLife.52212 (PMC7755393; doi:10.7554/eLife.52212)
Supplement: Supplementary file 3. [file elife-52212-supp3.docx]

**Supplementary File 3 Table 3:**

**Sequences of Primers for Quantitative RT-PCR**

| **Target gene** | **Species** | **Primer name** | **Sequence 5’-3’** |
| --- | --- | --- | --- |
| **18S rRNA** | Mouse | m-18S *f* | ACTCAACACGGGAAACCTCAC |
|  |  | m-18S *r* | GGACATCTAAGGGCATCACAG |
| **GAPDH** | Rat | R-GAPDH *f* | AGTTCAACGGCACAGTCAAG |
|  |  | R-GAPDH *r* | TACTCAGCACCAGCATCACC |
| **GLP-1R** | Rat | R-GLP1R *f* | TATTGGCTCATCATACGCTTG |
|  |  | R-GLP1R *r* | GTCTGCATTTGATGTCGGTCT |
| **β-arrestin1** | Rat | R-Barr-1 f | AGAGTCTATGTGACGCTGA |
|  |  | R-Barr-1 r | ACAAACAGGTCCTTGCGAAA |
| **β-arrestin2** | Rat | R-Barr-2 f | AGGACCGGCTGCTGAGGAAGCT |
|  |  | R-Barr-2 r | AGGCCTTTCCTGTATCCTCT |
| **Glut2** | Rat | R-Glut2 *f* | TCAGCCAGCCTGTGTATGCA |
|  |  | R-Glut2 *r* | TCCACAAGCAGCACAGAGACA |
| **Adcy8** | Rat | R-Adcy-8*f* | CAGTCTGGGCCTGAGGAAATT |
|  |  | R-Adcy-8*r* | AAGTCAGGTTCTTCAAGGGTA |
| **Syt8** | Rat | R-Syt8 *f* | CTTGCTGCTGGAGTCCTCTT |
|  |  | R-Syt8 r | CCAGGCCCACAGTCTCTTTA |
| **Syt7** | Rat | R-Syt7 f | TACAACCCCTCTGCCAACTC |
|  |  | R-Syt7 r | AGCCACACCTTCACATAGGG |
| **Ano1** | Rat | R-ANO1 f | GCAGGGAAGCTGAGTTTTTC |
|  |  | R-ANO1 r | CTGGATGGGGTCTGTGATCT |
| **UCP-1** | Mouse | M-UCP-1*f* | GGCATTCAGAGGCAAATCAGCT |
|  |  | M-UCP-1*r* | CAATGAACACTGCCACACCTC |
| **PPARα** | Mouse | M-Pparα-*f* | AGCTCACAGAATTTGCCAAG |
|  |  | M- Pparα-*r* | TTCCATGATGTCACAGAACG |
| **Pgc1α** | Mouse | M-Pgc1α-*f* | GCGGTTCTCACAGAGACACT |
|  |  | M-Pgc1α-*r* | CTAAGACCGCTGCATTCATT |
| **Pgc1α** | Rat | R-PGC1α-*f* | TGGAGTGACATAGAGTGTGC |
|  |  | R-PGC1α-*r* | GGGCTCATTGTTGTACTGGT |
| **Tfam** | Rat | R-Tfam-*f* | CGGCAGAAACGCCTAAAGAA |
|  |  | R-Tfam-*r* | AGGTGACTCATCCTTAGCCC |
| **ACAA2** | Rat | R-ACAA2-*f* | GCATGTGGGTTTACGTGTGG |
|  |  | R-ACAA2-*r* | CCGAAGCGCACATTTCTGAC |
| **CPT1A** | Rat | R-CPT1A-*f* | TTATCGTGGTGGTGGGTGTG |
|  |  | R-CPT1A-*r* | CCCAGAGCCCTGTACCAAAG |
| **CPT1A** | Mouse | M-CPT1A-f | TTATCGTGGTGGTGGGTGTG |
|  |  | M-CPT1A-*r* | ACGCCACTCACGATGTTCTT |
| **ACAA2** | Mouse | M-ACAA2-f | ACACCCTTTGGAGCTTACGG |
|  |  | M-ACAA2-*r* | TGCCCACGATGACACTATCG |

**Supplementary Table 3 legend:** Sequences of Primers for Quantitative RT-PCR as described in the table.
