# Supplementary material for: MS-275, a class 1 histone deacetylase inhibitor augments glucagon-like peptide-1 receptor agonism to improve glycemic control and reduce obesity in diet-induced obese mice
Source: eLife. 2020 Dec 22;9:e52212. doi: 10.7554/eLife.52212 (PMC7755393; doi:10.7554/eLife.52212)
Supplement: Peer review history

# Decision letter

**DOI:** 10.7554/eLife.52212.sa1

**Affiliations:** University of California, San FranciscoUnited States

In the interests of transparency, eLife publishes the most substantive revision requests and the accompanying author responses.

**Acceptance summary:**

Thank you for your hard work and flexibility in meeting the high standards of our reviewers and editors for publication in *eLife*. You have produced a valuable manuscript illustrating the metabolic changes that can be anticipated in vitro and more importantly in vivo with the mono- and combination therapies that you have studied. Given the high prevalence and morbidity and associated mortality of the twin epidemics of diabetes and obesity affecting our societies worldwide, your paper will be impactful in opening up new therapeutic options.

**Decision letter after peer review:**

[Editors’ note: the authors submitted for reconsideration following the decision after peer review. What follows is the decision letter after the first round of review.]

Thank you for choosing to send your work entitled "MS-275, A Class 1 HDAC Inhibitor Augments Sustained GLP-1 Receptor Agonism to Improve Glycemic Control and Reduce Obesity." for consideration at *eLife*. Your submission has been assessed by a Senior Editor in consultation with a member of the Board of Reviewing Editors. Although the work is of interest, we are not convinced that the findings presented have the potential significance that we require for publication in *eLife*.

Specifically, the reviewers have all brought up substantial experimental issues that would require a large series of more studies to be done along with a rewriting and refocusing of the manuscript. All feel the manuscript addresses interesting and important topics in metabolic health and signal transduction. But substantial efforts are needed to improve its overall priority and the reliability of the study conclusions. The study will be improved by attention to the comments of the reviewers.

Reviewer #1:

The study by Bele et al. identifies the class 1 HDAC inhibitor, a small molecule called MS-275, as enhancer of the GLP-1R agonist (Liraglutide) action. The work shows that the combined MS-275 and Liraglutide treatment has additive beneficial metabolic effects both in vitro and in vivo, and suggests that this combined therapy could improve the insulin secretion in vitro, and be used for treatment of obesity and glucose intolerance. MS-275 has been previously described to be sufficient for restoring the glucose-stimulated insulin secretion in presence of cytokines (doi: 10.1016/j.chembiol.2012.05.010.), and to prevent palmitate-induced β cell death (e.g. doi: 10.1371/journal.pone.0198016.). On the other hand, the finding that MS-275 can potentiate the effect of the GLP-1R agonist is novel, and could be of therapeutic relevance for potentiating the effects of the GLP-1R agonists. While I find the study of potential interest, in my view there are number of analysis, or controls that are missing, which are important to support the conclusion of the study for both main claims – the increased energy expenditure, and the enhanced glucose-stimulated insulin secretion.

1) The in vivo work shows marked improvement in the glucose tolerance and reduced weight gain during high fat diet feeding following the double (MS-275 +Liraglutide) treatment. While this could be of therapeutic relevance, authors should investigate markers of the liver damage and systemic inflammation during the treatment;

2) The data shown in Figure 7 shows increase in the Ucp1 expression. To claim that the fat browning is indeed affected, authors should quantify the rest of the browning markers in both the classical intrascapular brown fat, and the subcutaneous and visceral WAT, and provide at least H&E sections of the various fat depots;

3) Pertinent to point 2, one of the main conclusions of the work is that the energy expenditure is increased. To claim this, authors should investigate the oxygen consumption rates (OCR), or provide metabolic cage measurements. Ucp1 quantification is indicative of increased energy expenditure, but it is not sufficient to support that claim. Similarly, additional browning markers (PPARa, PGC1a) and the OCRs should be investigated in the primary differentiated adipocytes (shown in Figure 5) following the double treatment. The food intake should be shown for each week of the in vivo intervention;

4) The glucose phenotype in vivo should be complemented with insulin tolerance test, which would allow authors to discriminate the effect on the insulin sensitivity, versus the insulin secretion (also see point 5);

5) The in vivo study should be complemented with insulin measurements during the GTT, as well as with insulin quantification in isolated pancreatic islets from the treated mice, in support of the in vitro part of the study;

6) The gene expression profile observed after the in vitro treatment of the cultured β cells should be confirmed, or further investigated in isolated islets from the mouse experiment.

Reviewer #2:

In this manuscript Bele et al. show that inhibition of class I HDACs with the small molecule MS-275 (Entinostat) potentiates the effects of GLP-1R agonists on insulin secretion. They also observed that other class I HDAC inhibitors potentiate GLP-1R-mediated signaling. They hypothesized that "GLP-1R efficacy might be enhanced by increasing the expression of the auxiliary proteins supporting GLP-1R-mediated cAMP generation", implying that GLP-1 therapy could be enhanced. They show that "MS-275 enhances the expression of the genes involved in the GLP-1R signaling cascade which amplifies the efficacy of GLP-1R agonist liraglutide to improve glucose tolerance and enhance energy expenditure thereby providing a new direction of treatment of type2 diabetes and obesity".

The observation that inhibition of class I HDACs improves glucose tolerance and energy expenditure is not new. Others have previously shown that inhibition of HDACs, and in particular of class I HDACs, positively affects glucose and lipid metabolism by improving non-shivering thermogenesis in brown fat and by inducing browning of white fat (Galmozzi et al., 2013; Ferrari et al., 2017; Liao et al. Endocrinology 2018 vol. 159 pp. 2520-2527), and by enhancing skeletal muscle metabolic capacity through mitochondrial biogenesis (Gao et al. Diabetes 2009 vol. 58 pp. 1509-1517; Galmozzi et al., 2013). In addition, the main message the authors want to deliver is not clear. At the beginning of the manuscript the focus is on β cells and the mechanisms of insulin secretions. However, the Authors switched the focus on adipose tissue and energy expenditure. This makes the story somewhat confusing, as it is not clear what the improved insulin release elicited by liraglutide+MS-275 has to do with the enhanced energy expenditure in adipose tissue and consequently with reduced BW.

More specifically, I have the following major comments:

1) The concentration of liraglutide differs in experiments in Figure 1B (100 nM) and in Figure 1C (10 nM)? Why? Is there a rationale?

2) The time course experiment in Figure 1E, aimed at demonstrating the prolonged GLP-1R signaling, is not convincing. In the first place, it is not clear why liraglutide does not increase cAMP levels when incubated in the absence of MS-275. A time course experiment showing liraglutide initial increase of cAMP levels followed by reduced cAMP levels should be performed. Then, the authors should show that at time points when liraglutide alone does no longer enhance cAMP levels, pre-treatment with MS-275 extends the duration of increased cAMP levels (i.e., prolonged GLP-1R signaling in spite of receptor desensitization).

3) In Figure 3H-J, the Authors report enhanced glycolytic activity and glucose uptake. How was the expression of Glut2 in cells treated with MS-275? Any effect detected on Glut2 mRNA and/or protein levels? Furthermore, being mitochondrial ATP production important for GSIS, what about the expression of genes for mitochondrial proteins like TCA cycle, ETC, mitochondrial biogenesis (e.g., Ppargc1a, Tfam)?

4) In GSIS experiments, the concentration of liraglutide differed in BRIN-BD11 pancreatic β cells (0.1 nM, Figure 4B) vs. that used in cultured rat islets (1 nM, Gig. 4C): why? Also, in Materials and methods section the authors report 10 nM liraglutide in both experiments with BRIN-BD11 pancreatic β cells and cultured rat islets. Please, clarify what concentrations were used in the GSIS experiments.

5) In Figure 4G, the Authors try to provide an explanation of how MS-275 protects against palmitate-induced cell death by showing increased expression of some genes encoding enzymes of fatty acid β-oxidation. Some of these genes are upregulated (e.g., Acaa2, Hadh). However, the expression of Cpt1b and Cpt1c is reduced in cells treated with MS-275. Since, Cpt1b and Cpt1c catalyze the rate limiting step in FA β-oxidation, how do the authors reconcile these data with their hypothesis? Considering that malonyl-CoA levels regulate the activity of Cpt1, how was the expression of Acaca and Acacb, the enzymes catalyzing the conversion of acetyl-CoA to malonyl-CoA?

6) The data showing the effects of MS-275 on human adipocytes in Figure 5C are not novel. Other groups have shown that inhibition of HDACs induces (or class I HDACs are involved in) thermogenesis in adipose tissue (Galmozzi et al., 2013; Ferrari et al., 2017; Yuliana et al., Int J Mol Sci 2018 vol. 19 pp. 2436; Liao et al. Endocrinology 2018 vol. 159 pp. 2520-2527). Basically, here the authors show that the effects on human adipocytes are due to MS-275, whereas the combination of liraglutide+MS-275 does not elicit any additional effect. What is the novelty then?

7) In the legend to Figure 6B the Authors stated that "mean values of respective treatment groups represented with different letters indicates significant statistical difference", thus implying that the group treated with combination of MS-275+liraglutide did not show different glucose levels in the blood vs. liraglutide or MS-275 alone but only when compared against the group treated with vehicle alone. I do not see then the advantage of the combination therapy vs. the treatments with the two drugs alone and this in vivo study does not support the observations of in vitro experiments shown in previous figures.

8) On the same line, the IP-GTTs show differences only between liraglutide+MS-275 or MS-275 alone vs. liraglutide alone while no difference when comparing liraglutide+MS-275 vs. MS-275 alone. It seems that MS-275 does most of the job on glycemic control (Figure 6C and 6D) and that the combination therapy is not so effective when compared to MS-275 alone. In the Discussion the Authors infer that "the combination of MS-275 and Liraglutide therapy provided improvement in glucose tolerance that could not be matched by individual treatment with either entity at the indicated dose", however, when looking at Figure 6C and 6D this does not seem to be the case.

9) As for the body weight, it seems that there were no differences between combination therapy and monotherapies (Figure 6E the letters are the same in the three pharmacological treatments and the only difference was vs. the vehicle group). Interestingly, the group with combination therapy shows the highest increase of body weight during the two weeks suspension of the therapies (Figure 6F) and the greatest reduction of BW during the last four weeks of pharmacological treatments (Figure 6G). This suggests a significant advantage of the combination therapy against monotherapies. However, when assessing the normalized adipose mass, only retroperitoneal adipose tissue shows lower values in the liraglutide+MS-275 group vs. monotherapies (Figure 6Hii), while there are no differences in epididymal (Figure 6Hi) or mesenteric fat (Figure 6Hiii). The authors actually stated that all fat depots were reduced in combination therapy vs. monotherapies, however when looking at the figures this seems to be true only for retroperitoneal fat. This observation raises an important question: since only retroperitoneal fat is reduced more significantly with the combination therapy, how can the reduction of BW observed with liraglutide+MS-275 in Figure 6G be explained? It seems that the reduction of retroperitoneal fat alone cannot be sufficient to account for the reduction of BW.

10) Data in Figure 7 are not convincing. In panel A the Authors show that MS-275 alone and liraglutide+MS-275 increase glycerol release in murine adipocytes suggesting increased lipolysis. Nonetheless, this observation seems to be due to MS-275 alone rather than to the combination therapy. In panel B, instead, only the combination therapy shows increased Ucp1 mRNA level in retroperitoneal fat. How do the authors explain this discrepancy? Furthermore, independent investigations have already shown that inhibition of HDAC activity increases energy expenditure via enhanced thermogenesis and reduces BW in obese mice (Gao et al. Diabetes 2009 vol. 58 pp. 1509-1517; Galmozzi et al., 2013; Ferrari et al., 2017). Therefore, the effects on BW observed by the Authors could be explained mostly by MS-275 rather than the combination therapy. The Authors did not take into account the effect of MS-275 alone and the interpretation of their results is not convincing.

Reviewer #3:

Bele et al. describe work evaluating the effect of the HDAC inhibitor MS-275 on GLP-1R agonism in β cells, in adipocytes, and in a diet-induced obesity mouse model. The experimental data appear to be solid, and the findings that HDAC inhibition enhance the effects of liraglutide-induced GLP-1 signaling in the β cell are novel.

1) The main concern is that some of these results are close enough to previous reports as to question whether they should be included. In particular, the finding that MS-275 protects β cells from palmitate-induced apoptosis has been reported by Plaisance et al., 2014. The authors do indeed cite this paper, but the earlier paper was performed in mouse and human β cells, while this work was in a rat cell model. That difference seems quite minimal.

2) Similarly, MS-275 has been characterized extensively in a db/db mouse model in Galmozzi et al., 2013. Again, the authors cite the paper, but they should provide additional commentary in the Discussion about the differences with this study. The mechanisms by which HDAC inhibition improve glucose homeostasis and protect β cells from apoptosis have been described more fully in the literature than the authors give credit for.

[Editors’ note: further revisions were suggested prior to acceptance, as described below.]

Thank you for submitting your article "MS-275, A Class 1 HDAC inhibitor Augments GLP-1R Agonism to Improve Glycemic Control and Reduce Obesity in DIO Mice" for consideration by *eLife*. Your article has been reviewed by three peer reviewers, and the evaluation has been overseen by a Reviewing Editor and Anna Akhmanova as the Senior Editor. The reviewers have opted to remain anonymous.

The reviewers have discussed the reviews with one another and the Reviewing Editor has drafted this decision to help you prepare a revised submission.

We would like to draw your attention to changes in our revision policy that we have made in response to COVID-19 (https://elifesciences.org/articles/57162). Specifically, we are asking editors to accept without delay manuscripts, like yours, that they judge can stand as *eLife* papers without additional data, even if they feel that they would make the manuscript stronger. Thus the revisions requested below only address clarity and presentation.

Your paper addresses the metabolic effects of a Class 1 HDAC inhibitor in combination with GLP1 Receptor agonist.

With the guidance of statistical experts, please perform the statistical comparisons requested by the second reviewer, as detailed in the review. Please also strongly consider the comment of Reviewer 3 in which the reviewer requests that you remove the words related to synergy of the effects of the two compounds in the sub-title of the paper.

The following revisions are requested of the authors.

Summary:

All three previous reviewers have gone carefully through the revised manuscript and bring up the following key points that need to be addressed promptly by the authors for the finalized manuscript. Your attention is requested to the following points.

Revisions:

1) There are couple of points where the data is insufficient to claim the conclusions made in the study:

The importance of the energy expenditure and fat browning is not sufficiently investigated. Ucp1 and Cidea expression in visceral WAT is several orders of magnitude lower compared to BAT or subcutaneous WAT. It is unlikely that the 2 fold increase in Ucp1 and Cidea expression in the visceral WAT of the double compared to the single treatment, contributes to an increased energy expenditure and overall reduction of the body weight and fat mass. Additionally, authors choose not to investigate the OCR in their in vivo experiments. While H & E sections of fat depots upon single treatment may exist, the point was to see if the synergistic treatment promotes browning of fat, leading to increased energy expenditure. An increase in the Ucp1 and Cidea expression in the visceral fat is not sufficient to claim a meaningful potentiation of the fat browning that can enhance the energy expenditure. This remains a major weakness of the study.

Can you temper your conclusion without providing more data and without requiring a repeat review of your paper?

2) Can you efficiently revise the following parts of your paper to meet the reviewer's standard of statistical analysis and clarity of presentation, without requiring a repeat review of the paper?

a) In Figure 1F, the authors should add the statistical significance of the effects of liraglutide alone vs. basal (white bars), which is missing in the present version.

b) In their response to comment 7 to Figure 6B of the first version of the manuscript, the authors explained that mice treated with combined therapy display lower fasting glycemia. Are the differences between combination therapy and either liraglutide or MS-275 alone statistically significant? From their response, it seems that the combination therapy elicited a statistically significant reduction of fasting glycemia only when compared to vehicle alone, but not when compared to the monotherapies. It is suspected that the problem is mathematical/statistical, as the n=6 is too low to assume normality of distribution and for the statistical analysis they used a non-parametric method. The decision to use a nonparametric test should not be simply based on a normality test (e.g., D'Agostino-Pearson omnibus test or Shapiro-Wilk test). An automated decision simply taken from such tests may not be correct. In Figure 7A of the current version of the manuscript, the two groups of mice treated with monotherapies clearly show a reduction of fasting glycemia, however such differences are not statistically significant. Most likely, these two groups treated with monotherapies would reach significance by increasing the n. Likewise, for figures with data on IP-GTT and reduction of fat pads. Actually, statistical analysis with raw data of Figure 7 and 9 applying two different normality tests gave different results: the D'Agostino-Pearson omnibus test was not applicable because the "n" should be at least 8; however, when using the Shapiro-Wilk test, which works well if every value is unique, the four groups passed the normality test for fating glycemia (Figure 7A and B), IP-GTT (Figure 7D), and all fat depots in Figure 9, as opposed to what the Authors showed in the Excel files with raw data to Figure 7. Therefore, since data passed the Shapiro-Wilk test, when using ANOVA with Tukey's multiple test to reanalyze data in figure 7D (IP-GTT), it turned out that the two monotherapies are significantly different vs. control just like the combined therapy. Notably, the combination therapy was not statistically different vs. the two monotherapies. Likewise, with epididymal and mesenteric fat, while with retroperitoneal fat the combination therapy showed significant difference only vs. control group or liraglutide group, but not vs. MS-275 group (Figure 9). Only when considering fasting glycemia the combination therapy turned out to be significantly different from the monotherapies (Figure 7A and B). All these considerations raise serious questions about the conclusions drawn by the authors (i.e., combination therapy is better than monotherapies). It is a key point to assess the real advantage of the combination therapy over monotherapy, when the final read out is GTT and fat depot reduction. The authors should reconsider their interpretation revising their statistical analyses. With some parameters the combination therapy seems to be more effective than monotherapies (BW and fasting glycemia). However, when considering other parameters (fat depots, IP-GTT) the combined therapy does not yield better outcome.

With the input of statistician(s), can you address these two concerns efficiently?

3) The point of the manuscript appears to be the synergism between these two interventions (even comprising the running title), there should be included calculations of a combination index (CI) for the two treatments. This is not present in the manuscript, and it is uncertain that the combinatorial effects in Figure 9B are truly synergy. There may be a disconnect between that running title and the text in the rest of the manuscript, which doesn't quite play up the synergy as much. Can you remove the running title and/or provide the calculation requested?

[Editors' note: further revisions were suggested prior to acceptance, as described below.]

Thank you for resubmitting your work entitled "MS-275, A Class 1 HDAC inhibitor Augments GLP-1R Agonism to Improve Glycemic Control and Reduce Obesity in DIO Mice" for further consideration by *eLife*. Your revised article has been evaluated by Anna Akhmanova (Senior Editor) and a Reviewing Editor.

The manuscript has been significantly improved, but there are some remaining issues that need to be addressed before acceptance, as outlined below:

The reviewer has checked the stats and requests the following considerations. There are some statistical analyses and conclusions that need attention and corrections.

1) Bele et al. revised the statistical analyses of results applying the Shapiro-Wilk test for normality of distribution. However, it seems that the way the normality test was run is not completely appropriate as they calculated normality including data of all groups rather than calculating the normality group by group. The final output of statistically significant differences changes slightly in some instances.

a) For example, when analyzing the AUC of the GTT they come to the conclusion that "the glucose tolerance of the mice fed on HFD become comparable to the control group fed on the chow diet thereby demonstrating efficient the glucose-lowering ability of MS-275 and liraglutide combined therapy". Actually, the Shapiro-Wilk test for normality of distribution, considering one outlier value in the control HFD group (38130) shows normal distribution of values. It follows that the only statistically significant differences are "Ctrl vs. MS", "Ctrl vs. L+M" and "Ctrl vs. Chow", while all other comparisons were not statistically significant. Since all the treatments (i.e., both monotherapies and the combined therapy) were not statistically significant different vs. chow diet and because the treatment with MS-275 alone was not significantly different vs. the combined therapy, the conclusion should be that MS-275 monotherapy and combined therapy are both efficient.

b) Likewise, for the Shapiro-Wilk test for normality of distribution in the comparisons for epididymal, mesenteric and retroperitoneal fat. When calculating the normality for distribution, the authors pooled all the data while the normality should be calculated for each experimental group. This way, the normality test was passed with all groups and all three fat depots. For epididymal and mesenteric fat, both monotherapies and combined therapy reduce fat mass vs. vehicle controls, and there is no difference between combined therapy and both monotherapies. For retroperitoneal fat, only combined therapy reduces fat mass, MS-275 and combined therapies are significantly different vs. liraglutide, while combined therapy does not differ from MS-275. In sum, in no instances the combined therapy seems to be superior to MS-275 monotherapy in reducing any of the fat depots.

c) The asterisks for statistical significances should be modified accordingly in all figures.

d) In addition, I recommend running the normality tests considering each group separately and not pooling data of all groups together also for the comparisons of gene expression."

# Reply: Author response

**DOI:** 10.7554/eLife.52212.sa2

[Editors’ note: The authors appealed the original decision. What follows is the authors’ response to the first round of review.]

Reviewer #1:

The study by Bele et al. identifies the class 1 HDAC inhibitor, a small molecule called MS-275, as enhancer of the GLP-1R agonist (Liraglutide) action. The work shows that the combined MS-275 and Liraglutide treatment has additive beneficial metabolic effects both in vitro and in vivo, and suggests that this combined therapy could improve the insulin secretion in vitro, and be used for treatment of obesity and glucose intolerance. MS-275 has been previously described to be sufficient for restoring the glucose-stimulated insulin secretion in presence of cytokines (doi: 10.1016/j.chembiol.2012.05.010.), and to prevent palmitate-induced β cell death (e.g. doi: 10.1371/journal.pone.0198016.). On the other hand, the finding that MS-275 can potentiate the effect of the GLP-1R agonist is novel, and could be of therapeutic relevance for potentiating the effects of the GLP-1R agonists. While I find the study of potential interest, in my view there are number of analysis, or controls that are missing, which are important to support the conclusion of the study for both main claims – the increased energy expenditure, and the enhanced glucose-stimulated insulin secretion.

1) The in vivo work shows marked improvement in the glucose tolerance and reduced weight gain during high fat diet feeding following the double (MS-275 +Liraglutide) treatment. While this could be of therapeutic relevance, authors should investigate markers of the liver damage and systemic inflammation during the treatment;

To address the reviewer’s concerns we provide the following reply:

a) The HFD diet we provided (% kcal: protein 19.88, fat 59.0, carbohydrate 21.12, (Supplementary file 2—table 2)) did not increase the liver weight (Figure 9—figure supplement 1). We did not observe a fatty liver phenotype in control or those mice receiving monotherapy or dual therapy.

b) Following the reviewer’s suggestion, we conducted gene expression studies where we noted a significant increase of IGF1 in the liver upon the combined treatment with liraglutide and MS-275 (Figure 9—figure supplement 2). IGF1 has a direct anti-inflammatory effect on hepatic cells (1) and liver IGF1 has been reported to decrease during hepatic steatosis (2). We observed a 3.13± 0.15 fold increase (p<0.05) in the hepatic tissue of C57/BL6 mice that received dual therapy with liraglutide and MS-275 as reported in the study.

c) We would also wish to add that

i) Liraglutide has previously been reported to evoke an anti-inflammatory response. Luo et.al reported that the increased of α-SMA, IL-1β, TNF-α, and NF-κB in the liver of diet-induced obese diabetic mice was attenuated with liraglutide treatment (3). Liraglutide also reduces hepatic inflammation in rats with Diet-Induced Non-alcoholic Fatty Liver Disease (4).

ii) Liraglutide is a marketed drug for diabesity and MS-275 is currently in Phase3 with no report of liver damage or systemic inflammation having been reported in clinical trials. The new results pertaining to liver weight (Figure 9—figure supplement 1) and IGF-1 expression (Figure 9—figure supplement 2) are provided, and the information is added in the Discussion section of the revised text.

2) The data shown in Figure 7 shows increase in the Ucp1 expression. To claim that the fat browning is indeed affected, authors should quantify the rest of the browning markers in both the classical intrascapular brown fat, and the subcutaneous and visceral WAT, and provide at least H&E sections of the various fat depots;

3) Pertinent to point 2, one of the main conclusions of the work is that the energy expenditure is increased. To claim this, authors should investigate the oxygen consumption rates (OCR), or provide metabolic cage measurements. Ucp1 quantification is indicative of increased energy expenditure, but it is not sufficient to support that claim. Similarly, additional browning markers (PPARa, PGC1a) and the OCRs should be investigated in the primary differentiated adipocytes (shown in Figure 5) following the double treatment. The food intake should be shown for each week of the in vivo intervention;

We thank the reviewer for this comment. This is a cluster of comments and for convenience, we provided our reply to the comments sequentially.

a) Following the reviewer’s suggestion, OCR on differentiated adipocytes has been conducted. We used palmitate as a substrate (5) and used the Seahorse XF palmitate oxidation stress kit to assess the energy expenditure from fatty acid oxidation. As the results show, MS-275 promotes mitochondrial respiration in cultured differentiated adipocytes when palmitate was used as substrate contributing to the increase in ATP-linked respiration and Proton leak, which we did not observe upon liraglutide treatment.

The data presented in the revised manuscript enabled us to derive the following equation: 𝛥𝐸 = [𝑈𝐶𝑃1]. [𝐹𝑎𝑡𝑡𝑦 𝑎𝑐𝑖𝑑]. 𝛽𝑜𝑥𝑖𝑑𝑎𝑡𝑖𝑜𝑛 𝑓𝑙𝑢𝑥……….. (1) where ΔE = energy expenditure; [ Fatty acid] = Fatty acid concentration; [UCP1] = UCP 1 expression and β-oxidation flux is the superoxide generator at the mitochondrial matrix that initiates the oxidant quenching cycle driving the proton leak and the subsequent energy expenditure (Figure 10, revised manuscript)

The new data is provided as new Figure 6 and the corresponding text is added in the Results as well as in the Discussion. Figure 10 summarizes the model explaining the energy expenditure.

b) The reviewer has asked for an assessment of food intake through each week of in vivo intervention. Following the reviewer’s request, we repeated the bodyweight gain experiment and measured food intake vis-à-vis bodyweight gain every day for a period of 4 weeks. As Figure 8A (i) shows there is a significant reduction of food intake and subsequent reduction of body weight in mice receiving combined therapy of liraglutide and MS-275. The new data is provided as Figure 8A (i) and Figure 8A (ii) and the corresponding text is added.

c) As suggested by the reviewer we provided the gene expression data of PPAR α, PGC1 α, CIDEA, along UCP 1. However, we focused on visceral WAT as in this manuscript we are addressing the mechanism of reduction of weight gain upon liraglutide and MS-275 combined therapy. The new data is provided as (Figure 9B (i), (ii), (iii), and (iv)) and the corresponding text updated.

d) The H and E sections of fat depot upon MS-275 treatment (6) and liraglutide treatment (7) exist in the literature and repetition was therefore not conducted in this current manuscript.

4) The glucose phenotype in vivo should be complemented with insulin tolerance test, which would allow authors to discriminate the effect on the insulin sensitivity, versus the insulin secretion (also see point 5);

We politely state that we never claimed in this manuscript that MS-275 enhances the GLP-1R mediated insulin sensitivity. The manuscript is focused on MS-275 mediated augmentation of GLP-1R signaling that is manifested in the promotion of GSIS (Figure 1 and Figure 4). The context of insulin sensitivity needs an in-depth analysis of the mechanism which would be addressed in a separate study.

5) The in vivo study should be complemented with insulin measurements during the GTT, as well as with insulin quantification in isolated pancreatic islets from the treated mice, in support of the in vitro part of the study;

We thank the reviewer for the suggestion to measure insulin content. We, therefore, estimated the insulin content in the pancreatic tissue isolated from the chow diet-fed animals as well as from mice on HFD receiving liraglutide and MS-275 monotherapy, combined therapy, or the vehicle alone. HFD feeding significantly reduced the insulin content as compared to the group on chow diet. Combined treatment of HFD fed mice with MS-275 and liraglutide restored the insulin content to normal level (Figure 7E, p<0.05 Kruskal Wallis non-parametric test). We observed a partial restoration of the insulin content in HFD-fed mice treated with liraglutide or MS-275 as monotherapy. The new data is added as Figure 7E and the corresponding text is added in the Results. While we appreciate the suggestion for measuring insulin content, we politely point out that the focus of the manuscript is on the augmentation of GLP-1R signaling by Class1 HDAC inhibitor MS-275. At the dose of liraglutide, and more importantly, the regimen employed in the study, we do not think that real-time insulin measurement during IpGTT would provide any additional relevant information.

6) The gene expression profile observed after the in vitro treatment of the cultured β cells should be confirmed, or further investigated in isolated islets from the mouse experiment.

We thank the reviewer for the suggestion and conducted experiments following such advice. Our studies on cultured pancreatic β cells revealed that MS-275 stimulates GLP-1R mediated sustained cAMP generation and we report enhancement of Gαs and GLP-1R expression upon MS-275 treatment that augments GLP-1R induced cAMP generation. Following the reviewer’s suggestion, we also evaluated Gαs and GLP-1R protein expression in pancreatic tissue isolated from mice treated with liraglutide and MS-275 as monotherapy, or in combined therapy. Figure 7G reveals a 10.96 fold increase of Gαs expression upon liraglutide treatment, a 16.43-fold increase upon MS-275 treatment, and 21.75 fold increase upon liraglutide and MS-275 combined therapy, as compared to the HFD mice receiving saline as vehicle control. Similarly, we observed a 1.77 and 1.74 fold increase of GLP-1R expression upon liraglutide and MS-275 monotherapy and a 3.58-fold increase upon liraglutide and MS-275 combined therapy as compared to the HFD mice receiving vehicle control (Figure 7F). The results show that MS-275 enhances GLP-1R and Gαs expression both in vitro and in vivo to augment incretin receptor agonism. The new results are added as Figure 7F and Figure 7G and the corresponding text is added in the Results.

Reviewer #2:

[…]

More specifically, I have the following major comments:

1) The concentration of liraglutide differs in experiments in Figure 1B (100 nM) and in Figure 1C (10 nM)? Why? Is there a rationale?

Liraglutide is a GLP-1R agonist and Jant-4 is the antagonist of the receptor (9). The experiment studies the competition between an agonist and an antagonist in the presence of MS-275 to assess a signaling response. We carried out the titration of the agonist and antagonist to determine the liraglutide concentration of 10nM, as at this concentration of agonist Jant4 at 10 μM could completely reduce liraglutide mediated cAMP generation to the basal response. The experiment has been conducted to show that a GLP-1R antagonist can significantly reduce MS-275 mediated potentiation of liraglutide action.

2) The time course experiment in Figure 1E, aimed at demonstrating the prolonged GLP-1R signaling, is not convincing. In the first place, it is not clear why liraglutide does not increase cAMP levels when incubated in the absence of MS-275. A time course experiment showing liraglutide initial increase of cAMP levels followed by reduced cAMP levels should be performed. Then, the authors should show that at time points when liraglutide alone does no longer enhance cAMP levels, pre-treatment with MS-275 extends the duration of increased cAMP levels (i.e., prolonged GLP-1R signaling in spite of receptor desensitization).

We thank the reviewer for the comment. We incorporated new data to address the concerns and provide greater clarity. Also, we divide our reply into two parts (a and b) to adequately address the concern.

As a preamble to our reply, we request the reviewer to evaluate the time course experiment in the context of recent findings that not all GPCRS, especially Class B GPCRs, desensitize upon internalization as they continue generating cAMP at the plasma membrane and endosomes (10), (11), (12). The non-canonical concept of GPCR signaling considers endosomes as more than a conduit for GPCR trafficking as it supports the formation of the complex with the internalized receptor, β arrestin-1, and Gαs to generate sustained cAMP generation at endosomes (13). GLP-1R mediated cyclic AMP generation at endosomes was first reported from our laboratory (14) and we followed up with another article in Molecular Metabolism in 2017 (8) where we described that prolonged association of Gαs with the internalized activated receptor at Rab 5 endosomes contributes to the process. While cAMP generation is noted at the plasma membrane and the endosome for both of the incretin receptors (14), (8), (15), the nature of this signaling and as to whether it is continuous or discrete; the nature of when and how this signaling is terminated; and most importantly whether there is any ligand bias or specificity has yet to be elucidated.

We address the reviewer’s concerns based on this prior information.

a) The reviewer stated that it is not clear why liraglutide does not increase cAMP levels when incubated in the absence of MS-275.

Liraglutide increases cAMP in the absence of MS-275 at 5 min (from 252.15±49.53 to 1173.64±126.45 pmol cAMP/mg protein), 15 min (from 252.15±49.53 to 1462.54±106.66 pmol cAMP/mg protein), 30 min (from 252.15±49.53 to 1815.42±52.07) pmol cAMP/mg protein and 90 min (from 252.15±49.53 to 1446.05±144.93 pmol cAMP/mg protein) post ligand binding and internalization (Figure 1F clear bars). Comparing control and MS-275 treatment, we observed a significant increase in cAMP generation at 30 and 90-minute post internalization using liraglutide as the ligand for GLP-1R. The experiment has been carried out to highlight that MS-275 causes a significant increase in the cAMP generation when the activated receptor has achieved substantial intracellular localization at 30 min and 90 min post internalization. The data is in synchrony with Figure 1G which shows MS-275 significantly enhances GLP1R mediated cAMP generation when endosomal maturation is halted upon Bafilomycin treatment. The data highlight the efficacy of MS-275 to stimulate GLP-1R- mediated cAMP generation upon internalization. For greater clarity, we expressed Rab5A S34N in the pancreatic β-cell to block GLP1R internalization upon activation (16). As Figure 1H shows, Rab5A S34N expression reduces MS-275 stimulated GLP-1R signaling that impacts GLP-1R induced GSIS (Figure 4D). The new data is provided as Figure 1H and Figure 4D and the corresponding text is added on the main text.

b) The reviewer suggested that a time course experiment showing liraglutide initial increase of cAMP levels followed by reduced cAMP levels should be performed. Then, the authors should show that at time points when liraglutide alone does no longer enhance cAMP levels, pre-treatment with MS-275 extends the duration of increased cAMP levels.

We highly appreciate the suggestion. However, we would like to politely inform that unlike GLP-1Tmr (14) and Exendin-4 (8), we found that trafficking of liraglutide with the activated receptor follows different kinetics. We observe substantial cAMP generation even at 90-minute post internalization (Figure 1F). The corresponding localization of GLP-1R GFP as punctate dots in the cytoplasm was observed post the 90-minute time point (Figure 1E) which is in contrast to our previous observation with GLP-1Tmr where the activated receptor was found to be at the lysosomes at 90 min time point.

The study provides the first documentation of small molecule mediated augmentation of sustained GPCR signaling post internalization of the receptor. More importantly, we relate it to a functional consequence (GSIS). Since the context of the manuscript is on the functional assessment of GLP-1R upon MS-275 treatment, we believe that the determination of signal termination is beyond the scope of the present study. Contextually, we point out that as of today the termination of GPCR mediated endosomal cAMP generation has been established only in the case of parathyroid hormone receptor and that an entire article was devoted to describing the phenomenon (17)

We carried out a new kinetics assessment extending the time course till 90min (Figure 1F). We incorporated new data showing that prevention of internalization of activated receptor reduced MS275 mediated augmentation of GLP-1R signaling (Figure 1H) and consequently reduces GLP-1R induced GSIS (Figure 4D). The new data is provided as Figure 1H and Figure 4D; we believe that the corresponding text would provide additional clarity to the concept we proposed.

3) In Figure 3H-J, the Authors report enhanced glycolytic activity and glucose uptake. How was the expression of Glut2 in cells treated with MS-275? Any effect detected on Glut2 mRNA and/or protein levels? Furthermore, being mitochondrial ATP production important for GSIS, what about the expression of genes for mitochondrial proteins like TCA cycle, ETC, mitochondrial biogenesis (e.g., Ppargc1a, Tfam)?

a) We observe a significant increase in GLUT2 mRNA expression upon MS-275 treatment (Figure 3K)

b) We already provided the data that MS-275 does not alter mitochondrial respiration (Figure 3—figure supplement 1), but non-mitochondrial respiration is upregulated. Accordingly, there is no increase in the expression of mitochondrial genes ( NES=-1.629, Figure 3—figure supplement 1)

c) The expression of Ppargc1a and Tfam also remain unaltered in pancreatic β cells upon treatment with MS-275 as determined by real-time PCR (Figure 3—figure supplement 1)

4) In GSIS experiments, the concentration of liraglutide differed in BRIN-BD11 pancreatic β cells (0.1 nM, Figure 4B) vs. that used in cultured rat islets (1 nM, Gig. 4C): why? Also, in Materials and methods section the authors report 10 nM liraglutide in both experiments with BRIN-BD11 pancreatic β cells and cultured rat islets. Please, clarify what concentrations were used in the GSIS experiments.

a) The rat islet equivalents we prepared did not respond to 0.1nM liraglutide and hence 1nM concentration has been used.

b) The concentration mentioned in the Materials and methods section is a typographical error that has been corrected. We thank the reviewer for pointing this out.

5) In Figure 4G, the Authors try to provide an explanation of how MS-275 protects against palmitate-induced cell death by showing increased expression of some genes encoding enzymes of fatty acid β-oxidation. Some of these genes are upregulated (e.g., Acaa2, Hadh). However, the expression of Cpt1b and Cpt1c is reduced in cells treated with MS-275. Since, Cpt1b and Cpt1c catalyze the rate limiting step in FA β-oxidation, how do the authors reconcile these data with their hypothesis? Considering that malonyl-CoA levels regulate the activity of Cpt1, how was the expression of Acaca and Acacb, the enzymes catalyzing the conversion of acetyl-CoA to malonyl-CoA?

We thank the reviewer for the comment and provide additional data to address the queries. MS-275 treatment enhances the expression of key genes (Cpt-1A, ACADL/ACADM/ACADS, HADH, and ACAA2) in the β-oxidation pathway of pancreatic β cells. Upon overnight palmitate treatment, the expression of these genes becomes comparable in control and MS-275 treated cells indicating comparable β-oxidation flux (Figure 5B-E). However, in MS-275 treated cells there is upregulation of antioxidant enzymes such as Prdx1, Prdx4, Prdx6 as well as Gpx2, Txnrd1, and Txnrd3 that play a critical role in the cytoplasm to reduce peroxides and alleviate oxidative stress (Figure 5F). Consequently, we observed a decrease in the ROS generation as measured by carboxy-H_2_DCFDA assay (Figure 5G). The data as explained in Figure 5H describes the mechanism by which MS-275 prevents palmitate-induced death of pancreatic β cells. The new data is provided as Figure 5B, Figure 5H and the corresponding text are added in the Results and also in the Discussion.

6) The data showing the effects of MS-275 on human adipocytes in Figure 5C are not novel. Other groups have shown that inhibition of HDACs induces (or class I HDACs are involved in) thermogenesis in adipose tissue (Galmozzi et al., 2013; Ferrari et al., 2017; Yuliana et al., Int J Mol Sci 2018 vol. 19 pp. 2436; Liao et al. Endocrinology 2018 vol. 159 pp. 2520-2527). Basically, here the authors show that the effects on human adipocytes are due to MS-275, whereas the combination of liraglutide+MS-275 does not elicit any additional effect. What is the novelty then?

The reviewer’s comment has dealt with a very important perspective in drug discovery and we are indeed happy to elaborate on the subject. We added new data to probe the reviewer’s concern and epitomized the concept to an equation to provide greater clarity. As a preamble to our reply, we politely request the reviewer to assess the concept from the angle of pharmacodynamics, an essential branch of drug discovery where the impact of a single drug or drug combination is evaluated in the context of a particular biological response. A typical drug combination can display additivity, synergy, or interference.

MS-275–mediated enhanced oxidative metabolism and white adipose tissue (WAT) browning (6, 18) has been reported in the literature. However, the reports on the effect of GLP-1R signaling on energy expenditure were contradictory. While in some studies GLP-1 analogs have been shown to contribute to the fatty acid oxidation and WAT browning (19), other studies in animal models and humans report no change or even a decrease in energy expenditure upon incretin treatment (20, 21). So the objective of our study is to explore whether in regulating energy expenditure there is any synergy or additivity in response to two drugs used in combination, or whether one drug masks the effect of the other (interference).

The criteria assume further significance for two reasons:

i) We observed augmentation of GLP-1R mediated cAMP generation by MS-275 in pancreatic β cells ( indicative of an additive/synergistic relationship between two drugs) ii) Both liraglutide (a marketed drug for diabesity) and MS-275 (Phase 3 clinical trial for cancer) have been reported to reduce fat mass.

However, in the revised manuscript we replaced human adipocyte data for browning markers with the data on mouse visceral WAT as it reflects more closely the in vivo phenotype at the dosage regimen adapted in the study. The data we present (Figure 9B) describes a classical example of drug interactions in combinatorial therapy. As Figure 9B (i) shows, liraglutide has no effect on PPAR α mRNA at the dose we used in the study. The effect on combined therapy is completely an MS-275 effect. Figure 9B (ii) reflects on CIDEA expression. Here both liraglutide and MS-275 enhance CIDEA expression but on combination therapy, the CIDEA expression is the same as either monotherapy reflecting at the interference between liraglutide and MS-275 in regulating CIDEA expression. In the case of PGC1 α (Figure 9B (iii)) and UCP 1 (Figure 9B (iv)), we observe synergy at the indicated dose, with the effect of combined therapy being more than the linear additive effect of monotherapy.

To explore whether this synergy has any physiological consequence we carried out real-time respirometry with 3T3L1 adipocytes using palmitate as the substrate. As Figure 6A (i) shows, liraglutide exerted no effect on oxygen consumption rate (OCR) both in control and MS-275 treated cultured adipocytes when palmitate was used as substrate. We observed increased maximal respiration (Figure 6A (ii)), as well as ATP-linked respiration (Figure 6A (iii)) and the OCR linked to proton leak (Figure 6A (iv)) in MS-275- treated adipocytes. There was no such increase in ATP-linked respiration (Figure 6B (ii)) or proton leak (Figure 6B(iii)) with overnight liraglutide treatment. Replacement of palmitate with fat-free BSA diminished MS-275 mediated proton leak, which indicated that the β-oxidation flux and subsequent generation of reactive oxidants function as a throttle that drives energy expenditure. The data is consistent with the existing hypothesis that UCP 1 expression, though necessary, is not sufficient as other effectors are required to promote calorie burning (22).

The data evolves to an equation that states 𝛥𝐸 = [𝑈𝐶𝑃1]. [𝐹𝑎𝑡𝑡𝑦 𝑎𝑐𝑖𝑑]. 𝛽𝑜𝑥𝑖𝑑𝑎𝑡𝑖𝑜𝑛 𝑓𝑙𝑢𝑥 …. where ΔE = energy expenditure; [UCP1] = UCP 1 expression; [Fatty acid]=fatty acid concentration; and β-oxidation flux is the superoxide generator that drives the phenomenon. We propose that increased β-oxidation flux and the reactive oxidants generated drive energy expenditure in MS-275 treated adipocytes. “The oxidant quenching cycle” which is initiated with the generation of the superoxide is a by-product of the β-oxidation pathway, and it is throttled by the quenching of superoxides by the antioxidant enzymes that drive the proton leak, causing energy dissipation (Figure 10).

The reviewer enquired about the novelty:

We politely respond that the novelty in the study is the derivation of the equation and experimental validation that enhanced UCP 1 expression may not be the only criteria to drive increased energy expenditure. The electrochemical force that originates through superoxide generation in β-oxidation flux and driven by the oxidant quenching propels the proton leak enabling energy dissipation. This new concept is described in Figure 6A and Figure 6B and the text described in the manuscript.

7) In the legend to Figure 6B the Authors stated that "mean values of respective treatment groups represented with different letters indicates significant statistical difference", thus implying that the group treated with combination of MS-275+liraglutide did not show different glucose levels in the blood vs. liraglutide or MS-275 alone but only when compared against the group treated with vehicle alone. I do not see then the advantage of the combination therapy vs. the treatments with the two drugs alone and this in vivo study does not support the observations of in vitro experiments shown in previous figures.

On this point, we do not agree with the reviewer. However, we understand the reviewer’s concern and provide additional data to bring greater clarity.

i) We politely request to consider the raw fasting glucose values. The fasting blood glucose for

a)

combined therapy = 80.00±4.69 mg/dL

b)

Liraglutide = 89.17±8.77 mg/dL

c)

MS-275 = 96.50±8.63 mg/dL

The numbers show that the reduction of blood glucose is enhanced upon combined treatment.

ii) We carried out a statistical comparison for the difference between the groups as the reviewer suggested. We first evaluated the normality of the distribution. Since n=6 for each group is too small to assume normality (D'Agostino and Pearson omnibus normality test or Shapiro-Wilk normality test) we employed a nonparametric method Kruskal Wallis test (Dunn’s multiple comparisons) and the significance was ascertained at the level of p<0.05. As the data shows, only liraglutide and MS275 combined treatment group show a significant difference with respect to vehicle control. The new statistical evaluation is incorporated in the revised manuscript.

iii) To experimentally demonstrate the advantage of the combined therapy over the monotherapy we added new data on fasting blood glucose that has been evaluated in the acute mode at a time point when t_max_ of the two drugs are aligned. We decided on the acute dosing regimen based on the reported pharmacokinetics of liraglutide and MS-275 that has been extensively studied. In a new set of experiments the DIO mice were treated with a single dose of liraglutide (3nmol/kg body weight), or MS-275 (5mg /kg body weight), or a combination of the two drugs at the indicated dose. After 24h, the animals received a second dose following which they fasted for 5h and blood glucose was evaluated. As the data shows, mice that received liraglutide and MS-275 monotherapy or combined therapy had fasting blood sugar at 106.5±3.46 mg/dL, 110.8±2.86 mg/d and 91.33±3.49 mg/dL respectively as compared to 150.2±3.53 mg/dL that was observed in vehicle control. As Figure 7A reveals the group receiving combined treatment of liraglutide and MS-275 has significantly reduced fasting blood sugar as compared to vehicle control (p<0.001, Kruskal Wallis non-parametric test). The new data demonstrate the advantage of combined therapy and it is described in Figure 7A and the corresponding text is added in the manuscript.

8) On the same line, the IP-GTTs show differences only between liraglutide+MS-275 or MS-275 alone vs. liraglutide alone while no difference when comparing liraglutide+MS-275 vs. MS-275 alone. It seems that MS-275 does most of the job on glycemic control (Figure 6C and 6D) and that the combination therapy is not so effective when compared to MS-275 alone. In the Discussion the Authors infer that "the combination of MS-275 and Liraglutide therapy provided improvement in glucose tolerance that could not be matched by individual treatment with either entity at the indicated dose", however, when looking at Figure 6C and 6D this does not seem to be the case.

We disagree with the reviewer; however, we understand the concern and added more data for greater clarity. First of all, we would like to point out that we already have stated in our manuscript that MS-275, unlike liraglutide, is not an incretin receptor agonist and we are not comparing the efficacy of two agonists

We politely request the reviewer to consider the following context

a) Mammalian glucose homeostasis is regulated by an endogenous incretin system that comprises GLP1/GIP, which are the endogenous ligands for the corresponding GPCRS GLP-1R and GIPR. The receptor-ligand interaction activates the second messenger cAMP signaling pathway to stimulate GSIS. Liraglutide is a long-acting GLP-1R agonist and like the endogenous ligand GLP-1, it binds to GLP-1R activating the signaling cascade.

b) Incretin secretion is impaired in type 2D and the insulin content is reduced with the progressive loss of pancreatic β-cell mass.

c) We provided new data that shows that insulin content in islets is reduced to 32.26±0.031 % upon HFD feeding as compared to chow diet control. In mice on combined treatment or MS-275 and liraglutide monotherapy, the insulin content is 92.02±0.05%, 87.38±0.05%, and 74.16 ±0.09% respectively (Figure 7E). There is also significant upregulation of GLP-1R and Gαs expression upon MS275 and liraglutide combined therapy as compared to monotherapy (Figure 7F and 7G).

As incretin secretion from entero-endocrine cells is reduced in type2D the objective here is to test whether the liraglutide function could be augmented upon MS-275 treatment. In this study we show upregulation of the GLP-1R signaling pathway in vitro as well as in vivo. We have chosen IpGTT, as intraperitoneal glucose load dose is believed not to stimulate incretin secretion from entero-endocrine cells through activation of the entero-insular axis

With this background, we request the reviewer to consider the results in Figure 7C and D. As the glucose excursion data shows a clear decrease reflective of increased efficacy when using the two agents simultaneously.

Mean AUC for vehicle control = 29,322±1,764

Mean AUC for combined therapy = 14,809 ±1,261

Mean AUC for liraglutide alone = 22,595 ±3,701

Mean AUC for MS-275 alone = 17,733 ±1,108

The numbers show that animals receiving combined therapy have better glycemic control than those receiving either of the monotherapy. We report that glucose excursion on combined therapy is less than even the chow diet control group and points out that reported literature shows that MS275 alone, at twice the dose used in this study, could not reduce to normoglycemia (Figure 2D, Galmozzi et.al, 2013).

To address the reviewer’s emphasis on the statistical comparison between the groups we carried out Kruskal Wallis test (Dunn’s multiple comparison test) as the data (n=6) is considered too small to be considered as normally distributed (D'Agostino and Pearson omnibus normality test or Shapiro-Wilk normality test). The significance was ascertained at p<0.05. As Figure 7D shows only liraglutide and MS-275 combined therapy shows a significant difference at the dose we used for the study, as compared to the vehicle control.

However, our study has a limitation that we mention in the revised Discussion. MS-275 monotherapy may activate the entero-insular axis and stimulate GLP-1 secretion. In this context, GLP-1R/GIPR double knockout mice would be an ideal control, which, unfortunately, we do not have access.

The new data on insulin content (Figure 7E), GLP1R, and Gαs expression (Figure 7Fand G) and the corresponding text has been added in the revised manuscript. The limitation of the study regarding MS-275-mediated assessment of the entero-insular axis is mentioned.

9) As for the body weight, it seems that there were no differences between combination therapy and monotherapies (Figure 6E the letters are the same in the three pharmacological treatments and the only difference was vs. the vehicle group). Interestingly, the group with combination therapy shows the highest increase of body weight during the two weeks suspension of the therapies (Figure 6F) and the greatest reduction of BW during the last four weeks of pharmacological treatments (Figure 6G). This suggests a significant advantage of the combination therapy against monotherapies. However, when assessing the normalized adipose mass, only retroperitoneal adipose tissue shows lower values in the liraglutide+MS-275 group vs. monotherapies (Figure 6Hii), while there are no differences in epididymal (Figure 6Hi) or mesenteric fat (Figure 6Hiii). The authors actually stated that all fat depots were reduced in combination therapy vs. monotherapies, however when looking at the figures this seems to be true only for retroperitoneal fat. This observation raises an important question: since only retroperitoneal fat is reduced more significantly with the combination therapy, how can the reduction of BW observed with liraglutide+MS-275 in Figure 6G be explained? It seems that the reduction of retroperitoneal fat alone cannot be sufficient to account for the reduction of BW.

We do not agree with the reviewer on these points. We divided the reviewer’s comment into four parts and sequentially address them. We added new data to offer greater support and clarity.

a) As for the bodyweight, it seems that there were no differences between combination therapy and monotherapies (Figure 6E the letters are the same in the three pharmacological treatments and the only difference was vs. the vehicle group).

We are very sorry for our typological error with the letters. We corrected it in the revised version, (6E is 8D in the revised version). The significant difference is obvious relative to vehicle control. There is an increase in the body weight gain whereas with dual therapy there is a decrease in the body weight gain (the trend line is on the different quadrant). Dual therapy is significantly different from vehicle control as well as from the other two monotherapies (***p<0.001 as compared to vehicle control; **p<0.01 as compared to liraglutide monotherapy, * p<0.05 as compared to MS-275 monotherapy, one-way ANOVA, Tukey’s multiple comparisons). Figure 8B(ii) represents the body weight gain from the 10-17^th^ week in the progression of diet-induced obesity and the letters signifying statistical differences are corrected. An inset has been added depicting the net AUC of the bodyweight gain corresponding to each therapy. The corresponding text is described in the Results.

b) Interestingly, the group with combination therapy shows the highest increase of body weight during the two weeks suspension of the therapies (Figure 6F)

Figure 6F, (Figure 8B (iii), revised version). Regarding the comment of the reviewer we politely point out that the data aligns with The SCALE Maintenance randomized clinical study where similar weight gain was noticed after the withdrawal of liraglutide post 56- week treatment during follow up (23). Figure 6F (Figure 8B in the revised version) depicts this reversibility (i.e. highest increase of body weight during the two weeks in the suspension of the therapies ) signifies that the reduction we observed in 10 to 17^th^ week of treatment is a consequence of GLP-1R agonism, not due to drug-related toxicities.

c) Figure 6G: The reviewer has enquired the rationale behind the greatest reduction of BW during the last four weeks of pharmacological treatments (Figure 6G).

We thank the reviewer for the comment. We repeated the entire experiment and measured the body weight gain vis-à-vis food intake. In the new study design, the mice on the high-fat diet were not exposed to drug treatment until they attain a hyperglycemic state and obese phenotype. Post attainment of diabesity the mice were treated with saline (vehicle control), liraglutide, MS-275, and liraglutide + MS275 combined for a period of 4 weeks. The weight gain and food intake were measured every day during these 4 weeks. As Figure 8A (ii) (new data, revised version) shows, there was a significant reduction of body weight gain in groups treated with liraglutide and MS-275 combined therapy (-20.41±2.98%) The decrease was significant as compared to MS-275 monotherapy where we observed a reduction of -8.69 ±3.35% (p<0.001). To determine the rationale for the decrease in bodyweight gain we measured the cumulative food intake (Figure 8A (i), new data, revised version). As the data shows, there is a significant decrease in cumulative food intake only in the group treated with liraglutide and MS-275 combination. The new data is presented in Figure 8A (i) and Figure 8A (ii) and the corresponding text has been added to the Results.

d) Figure 6H, reviewer only appreciated the significant decrease in retroperitoneal fat but not in epididymal (Figure 6Hi) or mesenteric fat and infers that reduction of retroperitoneal fat alone cannot be sufficient to account for the reduction of BW.

The data is represented as fold over control and presented as a bar graph with detailed statistical analysis and significance level at α=0.05 revised manuscript to convince the reviewer The data demonstrates that there is a reduction of fat mass in all visceral fat depots assessed. Epididymal Fat: combined therapy reduces the epididymal fat mass to 0.34±0.09 fold as compared to vehicle control. The reduction is 0.50 ±0.06 fold in mice receiving liraglutide monotherapy and 0.45±0.02 fold in mice receiving MS-275 monotherapy. For statistical comparison between the groups we carried out the Kruskal Wallis test (Dunn’s multiple comparison test) as the data (n=6) is considered too small to be considered as normally distributed (D'Agostino and Pearson omnibus normality test or Shapiro-Wilk normality test). The significance was ascertained at p<0.05. As Figure 9A (i) revealed, only the group receiving combined therapy exhibited a significant reduction of epididymal fat mass (**p<0.01, Kruskal Wallis non-parametric test). Mesenteric Fat: combined therapy causes a 10-fold reduction as mesenteric fat mass is reduced to 0.10±0.05 fold upon combined therapy as compared to vehicle control. In the case of liraglutide and MS275 monotherapy, the reduction is 0.45-±0.08 fold and 0.36 ± 0.04 fold respectively. As Figure 9A (ii) revealed, only the group receiving combined therapy exhibited a significant reduction of mesenteric fat mass (***p<0.001, Kruskal Wallis non-parametric test). Retroperitoneal Fat: combined therapy reduces the retroperitoneal fat mass to 0.32 ±0.09 fold as compared to vehicle control. In the case of MS-275 monotherapy, the reduction is 0.55 ±0.09 fold. However, with liraglutide monotherapy, there is no reduction of retroperitoneal fat mass. As Figure 9A (iii) revealed, only the group receiving combined therapy exhibited a significant reduction of retroperitoneal fat mass (*p<0.05, Kruskal Wallis non- parametric test).

The data thus shows that all the visceral fat depots are significantly reduced upon MS-275 and liraglutide combined therapy. The reduction of fat mass is presented as fold over control in Figure 9A(i), Figure 9A(ii), and Figure 9(A iii), and the corresponding text is added in the manuscript.

10) Data in Figure 7 are not convincing. In panel A the Authors show that MS-275 alone and liraglutide+MS-275 increase glycerol release in murine adipocytes suggesting increased lipolysis. Nonetheless, this observation seems to be due to MS-275 alone rather than to the combination therapy. In panel B, instead, only the combination therapy shows increased Ucp1 mRNA level in retroperitoneal fat. How do the authors explain this discrepancy? Furthermore, independent investigations have already shown that inhibition of HDAC activity increases energy expenditure via enhanced thermogenesis and reduces BW in obese mice (Gao et al. Diabetes 2009 vol. 58 pp. 1509-1517; Galmozzi et al., 2013; Ferrari et al., 2017). Therefore, the effects on BW observed by the Authors could be explained mostly by MS-275 rather than the combination therapy. The Authors did not take into account the effect of MS-275 alone and the interpretation of their results is not convincing.

Figure 7 (Figure 10 in the revised manuscript) has been modified with the incorporation of the new data. However, before addressing the reviewer’s concern we evaluated the references that the reviewer cited

Reviewer #3:

Bele et al. describe work evaluating the effect of the HDAC inhibitor MS-275 on GLP-1R agonism in β cells, in adipocytes, and in a diet-induced obesity mouse model. The experimental data appear to be solid, and the findings that HDAC inhibition enhance the effects of liraglutide-induced GLP-1 signaling in the β cell are novel.

Authors thank the reviewer for commenting on the data as solid and the effects of liraglutide-induced GLP-1 signaling in the pancreatic β-cell as novel.

1) The main concern is that some of these results are close enough to previous reports as to question whether they should be included. In particular, the finding that MS-275 protects β cells from palmitate-induced apoptosis has been reported by Plaisance et al., 2014. The authors do indeed cite this paper, but the earlier paper was performed in mouse and human β cells, while this work was in a rat cell model. That difference seems quite minimal.

We politely state that the data we presented is all about the mechanism of palmitate-induced pancreatic β-cell death, species specificity is of tangential importance. We added new data to provide a novel mechanism in the prevention of pancreatic β-cell death that complements the observation on the restoration of insulin content in vivo (Figure 7E revised manuscript). Plaisance et.al (25) reported palmitate induced cell death, but the mechanism was incompletely understood. In our present manuscript, we have shown that palmitate treatment generates ROS. However, antioxidant enzymes like Prdx1, Prdx4, Prdx6 as well as Gpx2, Txnrd1, and Txnrd3 are upregulated upon MS-275 treatment (Figure 5F) that quench ROS generation. Consequently, we observed a decrease in ROS generation as measured by carboxy-H_2_DCFDA assay upon MS-275 treatment (Figure 5G). The data as explained in Figure 5H describes the mechanism by which MS-275 prevents palmitate-induced death of cultured pancreatic β cells. The new data is provided in (Figure 5B-H) and the corresponding text is described in the Results, and also in the Discussion.

2) Similarly, MS-275 has been characterized extensively in a db/db mouse model in Galmozzi et al., 2013. Again, the authors cite the paper, but they should provide additional commentary in the Discussion about the differences with this study. The mechanisms by which HDAC inhibition improve glucose homeostasis and protect β cells from apoptosis have been described more fully in the literature than the authors give credit for.

We disagree with the reviewer as Class1 HDAC inhibitor mediated regulation of incretin signaling has never been reported. This manuscript is not about the characterization of MS-275, rather the present document establishes three concepts that are previously unreported.

a) The manuscript deals with improved glucose homeostasis through small molecule mediated augmentation of sustained incretin receptor signaling (small molecule has been identified by unbiased screening that happens to be an HDAC inhibitor). We provided additional data in the revised manuscript to show that MS-275 augments sustained incretin receptor signaling upon receptor internalization that modulated GSIS and reports that this is the first documentation of small molecule mediated augmentation of sustained incretin receptor signaling in the context of a particular physiological response.

b) MS-275 has been used as a tool to derive the equation of energy expenditure

𝐸 = [𝑈𝐶𝑃1].[𝐹𝑎𝑡𝑡𝑦 𝑎𝑐𝑖𝑑]. 𝛽𝑜𝑥𝑖𝑑𝑎𝑡𝑖𝑜𝑛 𝑓𝑙𝑢𝑥

We humbly state that the equation is yet unreported and represents a new concept (described in Figure 6A and Figure 6B) and the text described in the manuscript. However, since the model we proposed aligns with Spiegelman’s seminal observation on mitochondrial ROS (26), (22) we have suitably cited them.

c) The third fundamental contribution is MS-275 mediated augmentation of antioxidant enzymes that quench reactive oxidant species. We have shown that MS-275 reduces ROS generated upon overnight palmitate exposure that has not been earlier reported (Figure 5F, 5G, 5H). Using RNA seq we have shown that the antioxidant enzyme isoforms (Prdx1, Prdx4, Prdx6) that localize in the cytoplasm are upregulated providing a mechanism of ROS quenching and indicative of the reduction of ROS due to peroxisomal beta-oxidation (Figure 5H). A pioneering review of Jean Jonas(27) has helped in the formulation of the concept presented in the study and has been cited in the text. The new data is provided as Figure 5B-Figure 5H and the corresponding text are added in Results section and also in the Discussion.

[Editors’ note: what follows is the authors’ response to the second round of review.]

Revisions:

1) There are couple of points where the data is insufficient to claim the conclusions made in the study:

The importance of the energy expenditure and fat browning is not sufficiently investigated. Ucp1 and Cidea expression in visceral WAT is several orders of magnitude lower compared to BAT or subcutaneous WAT. It is unlikely that the 2 fold increase in Ucp1 and Cidea expression in the visceral WAT of the double compared to the single treatment, contributes to an increased energy expenditure and overall reduction of the body weight and fat mass. Additionally, authors choose not to investigate the OCR in their in vivo experiments. While H & E sections of fat depots upon single treatment may exist, the point was to see if the synergistic treatment promotes browning of fat, leading to increased energy expenditure. An increase in the Ucp1 and Cidea expression in the visceral fat is not sufficient to claim a meaningful potentiation of the fat browning that can enhance the energy expenditure. This remains a major weakness of the study.

Can you temper your conclusion without providing more data and without requiring a repeat review of your paper?

We revised our conclusion as suggested and removed energy expenditure in the context of the reduction of body weight gain and the decrease in adipose fat mass.

Following changes have been made in the conclusion in the revised manuscript:

i) We removed energy expenditure in the context of the reduction of body weight gain and a decrease in adipose fat mass. We just reported the significant upregulation of UCP1 in retroperitoneal WAT upon combined therapy of liraglutide and MS-275.

ii) We stated that the statistically significant reduction in the food intake accounts for the drastic weight loss in the mice receiving combined therapy.

iii) We clarified that MS-275 is the key contributor to energy expenditure and the in vitro data we provided explains the mechanism that regulates the phenomenon.

iv) We stated that the energy equation we proposed is derived from the in vitro data.

v) We clearly stated in the manuscript that the lack of in vivo assessment of energy expenditure is a limitation of the study.

The Results and the Discussion section has been rewritten to accommodate the revised conclusion.

2) Can you efficiently revise the following parts of your paper to meet the reviewer's standard of statistical analysis and clarity of presentation, without requiring a repeat review of the paper?

a) In Figure 1F, the authors should add the statistical significance of the effects of liraglutide alone vs. basal (white bars), which is missing in the present version.

To comply with the reviewer’s request we plotted Figure 1F differently and presented as Figure 1—figure supplement 3 to show the statistical significance of liraglutide alone vs basal both in control and MS-275 treated cells (Figure 1—figure supplement 3).

The information is added in the text and a new supplementary figure is added as supplementary information.

b) In their response to comment 7 to Figure 6B of the first version of the manuscript, the authors explained that mice treated with combined therapy display lower fasting glycemia. Are the differences between combination therapy and either liraglutide or MS-275 alone statistically significant? From their response, it seems that the combination therapy elicited a statistically significant reduction of fasting glycemia only when compared to vehicle alone, but not when compared to the monotherapies. It is suspected that the problem is mathematical/statistical, as the n=6 is too low to assume normality of distribution and for the statistical analysis they used a non-parametric method. The decision to use a nonparametric test should not be simply based on a normality test (e.g., D'Agostino-Pearson omnibus test or Shapiro-Wilk test). An automated decision simply taken from such tests may not be correct. In Figure 7A of the current version of the manuscript, the two groups of mice treated with monotherapies clearly show a reduction of fasting glycemia, however such differences are not statistically significant. Most likely, these two groups treated with monotherapies would reach significance by increasing the n. Likewise, for figures with data on IP-GTT and reduction of fat pads. Actually, statistical analysis with raw data of Figure 7 and 9 applying two different normality tests gave different results: the D'Agostino-Pearson omnibus test was not applicable because the "n" should be at least 8; however, when using the Shapiro-Wilk test, which works well if every value is unique, the four groups passed the normality test for fating glycemia (Figure 7A and B), IP-GTT (Figure 7D), and all fat depots in Figure 9, as opposed to what the Authors showed in the Excel files with raw data to Figure 7. Therefore, since data passed the Shapiro-Wilk test, when using ANOVA with Tukey's multiple test to reanalyze data in Figure 7D (IP-GTT), it turned out that the two monotherapies are significantly different vs. control just like the combined therapy. Notably, the combination therapy was not statistically different vs. the two monotherapies. Likewise, with epididymal and mesenteric fat, while with retroperitoneal fat the combination therapy showed significant difference only vs. control group or liraglutide group, but not vs. MS-275 group (Figure 9). Only when considering fasting glycemia the combination therapy turned out to be significantly different from the monotherapies (Figure 7A and B). All these considerations raise serious questions about the conclusions drawn by the authors (i.e., combination therapy is better than monotherapies). It is a key point to assess the real advantage of the combination therapy over monotherapy, when the final read out is GTT and fat depot reduction. The authors should reconsider their interpretation revising their statistical analyses. With some parameters the combination therapy seems to be more effective than monotherapies (BW and fasting glycemia). However, when considering other parameters (fat depots, IP-GTT) the combined therapy does not yield better outcome.

With the input of statistician(s), can you address these two concerns efficiently?

In compliance with the suggestions, we consulted the statistician (mentioned in the acknowledgment) to address the concerns. As mentioned by the reviewer, we tested for normality using the Shapiro-Wilk normality test before the application of a statistical test. Wherever the distribution is normal, we applied one-way ANOVA (Tukey’s multiple comparisons) for statistical comparison and when the distribution is non-normal, Kruskal –Wallis multiple comparisons are applied. The distribution of the data is provided in the respective source data. To meet the clarity of the presentation, the reviewer’s question on the statistical significance of the differences between combination therapy and either liraglutide or MS-275 monotherapy is described for glucose-lowering; body-weight reduction, and the reduction of visceral adiposity in the revised manuscript.

Here the Null hypothesis (H_0_) is that the data follow a normal distribution and the alternative hypothesis (H_1_) is that the data does not follow a normal distribution. If p> α at the significance level of 0.05 we fail to reject the null hypothesis; on the contrary, if p< α at the significance level of 0.05 we reject the null hypothesis.

The case-specific analysis of the Shapiro-Wilk normality test is as follows:

Fasting glycemia acute (Figure 7A):

The distribution of the data is shown in Figure 7—source data 1. As the data reveals the distribution represents a Gaussian curve with the mean (114.7) concurring with the median (111.0) and p=0.0530052 (Shapiro-Wilk normality test). Since p> α at the significance level of 0.05, we consider the distribution normal and the parametric test, one-way ANOVA, Tukey’s multiple comparisons have been employed.

We provided the fasting sugar values in the text and mentioned that though both monotherapy and combined therapy revealed a significant decrease in the blood glucose, mice receiving the combined therapy displayed a superior blood glucose reduction as compared to liraglutide monotherapy (p<0.05, one –way ANOVA Tukey’s multiple comparison test) or MS-275 monotherapy (p<0.01, one –way ANOVA Tukey’s multiple comparison test)

The statistical interpretation is added in the text.

Fasting glycemia repeat dosing (Figure 7B):

The distribution of the data is shown in Figure 7—source data 1. As the data shows, the shape of the curve is asymmetrical (right-skewed) and the mean (101.54) does not concur with the median (94.5). Moreover p=0.0293958 (Shapiro-Wilk normality test). Since p< α at the significance level of 0.05, we accept the alternative hypothesis (H_1_) and consider the distribution non-normal. Accordingly, the Kruskal -Wallis non- parametric test is employed to ascertain the statistical difference.

We provided the fasting glucose values in the text and mentioned that though the group receiving the combined therapy only displayed a statistically significant reduction of fasting blood glucose as compared to the vehicle control (Kruskal Wallis non-parametric test; p<0.05), we did not observe a significant difference between monotherapies and combined therapy when blood glucose was assessed after repeat dosing.

The statistical interpretation is incorporated in the Results section.

IpGTT AUC (Figure 7D):

The distribution of the data is shown in Figure 7—source data 1.The shape of the curve is asymmetrical (right-skewed) p=0.00127996 (Shapiro-Wilk normality test). Since p< α at the significance level of 0.05, we accept the alternative hypothesis (H_1_) and consider the distribution non-normal. Accordingly, the Kruskal -Wallis non- parametric test is employed to ascertain the statistical difference. We stated that as the corresponding area under the curve (AUC) revealed, only the group that received the MS-275 and liraglutide combined therapy showed statistically significant improved glycemic control (p<0.05; Kruskal Wallis nonparametric test) in comparison to the group receiving normal saline as the vehicle (Figure 7D). Glucose tolerance was improved with MS-275 monotherapy as well. and we did not observe a statistically significant difference in glucose tolerance between MS-275 monotherapy and the combined therapy (Figure 7*C,* Figure 7*D*), However, when treated with the combined therapy, the glucose tolerance of the mice fed on HFD become comparable to the control group fed on the chow diet thereby demonstrating efficient the glucose-lowering ability of MS-275 and liraglutide combined therapy.

The statistical analysis and the clarification are incorporated in the Results section.

We also candidly acknowledge that our study has a limitation that we mentioned in the Discussion. MS-275 monotherapy may activate the entero-insular axis and stimulate GLP-1 secretion. In this context, GLP-1R/GIPR double knockout mice would be an ideal control to demonstrate statistical segregation between monotherapy and combined therapy, which, we do not have access to.

Insulin content (Figure 7E):

The distribution of the data is shown in Figure 7—source data 1. Shapiro-Wilk normality test revealed p=0.002642. Since p< α at the significance level of 0.05 we accept the alternative hypothesis (H_1_) and consider the distribution nonnormal. Accordingly, the Kruskal -Wallis non- parametric test is employed to ascertain the statistical difference

The statistical analysis and the corresponding clarification are incorporated in the Results section .

VISCERAL WAT:

Epididymal WAT Figure 9A (i) and Mesenteric WAT Figure 9A (iI)

The distribution of the epididymal WAT and mesenteric WAT is shown in Figure 9— source data 1 (Figure 9A). In the case of epididymal WAT, the distribution is asymmetrical (right-skewed), and as the Shapiro-Wilk normality test revealed p=0.00682397. Since p<α at 0.05, we considered the distribution non-normal. Accordingly, the Kruskal -Wallis non- parametric test is employed to ascertain the statistical difference.

In the case of the distribution of Mesenteric WAT, the curve is asymmetrical (right-skewed) and as the Shapiro-Wilk normality test revealed, p=0.0116927. Since p<α at 0.05, we considered the distribution non-normal. Accordingly, the Kruskal -Wallis non- parametric test is employed to ascertain the statistical difference.

We provided the fold reduction values in the text and mentioned that though the group that received combined therapy only showed the statistically significant reduction of epididymal and mesenteric WAT as compared to the vehicle control, we did not observe the statistically significant difference when the epididymal and mesenteric WAT reduction was compared between the combined therapy and either of the monotherapies.

Retroperitoneal white adipose tissue (WAT) (Figure 9*A (iii)*):

The distribution is perfectly Gaussian (Figure 9—source data 1); the Shapiro-Wilk normality test reveals p value = 0.220761; since p>α, we accept H_0_ and considered the distribution normal. Accordingly, one-way ANOVA (Tukey’s multiple comparison test) was employed.

We provided the fold reduction of the retroperitoneal WAT of the treated groups and corresponding statistical significance vis-à-vis vehicle control and also mentioned that the reduction of retroperitoneal fat mass upon combined therapy or MS-275 monotherapy is statistically significant as compared to the group receiving only liraglutide therapy.

The statistical analysis and the clarification are incorporated in the Results section.

Retroperitoneal WAT gene expressions:

Expression of PPAR α, CIDEA, PGC1α, and UCP1.

As Figure 9—source data 1 shows, the distribution of all the genes is asymmetric, and since p<α at 0.05 we consider the distribution non-normal and accordingly the Kruskal-Wallis non-parametric test is applied to ascertain statistical significance in all the four cases.

The statistical analysis and the clarification are incorporated in the Results section.

3) The point of the manuscript appears to be the synergism between these two interventions (even comprising the running title), there should be included calculations of a combination index (CI) for the two treatments. This is not present in the manuscript, and it is uncertain that the combinatorial effects in Figure 9B are truly synergy. There may be a disconnect between that running title and the text in the rest of the manuscript, which doesn't quite play up the synergy as much. Can you remove the running title and/or provide the calculation requested?

We accept the suggestion of the reviewer.

Figure 9B pertains to the gene expression data measured at a single concentration of MS-275 (5μM) or liraglutide (100nM) or a combination of the two drugs. To apply the combination index (CI) formula, the necessary isobologram has to be constructed (1). In the case of Figure 9B, CI could not be applied without additional data points. The term synergy has been used as, following the definition, the total effect was found to be greater than the sum of the effect exerted by the individual components, in this case, the two medicinal agents. However, rather than debating on this point, we accept the request to remove synergy from the running title as well as from the article.

[Editors' note: further revisions were suggested prior to acceptance, as described below.]

The reviewer has checked the stats and requests the following considerations. There are some statistical analyses and conclusions that need attention and corrections.

1) Bele et al. revised the statistical analyses of results applying the Shapiro-Wilk test for normality of distribution. However, it seems that the way the normality test was run is not completely appropriate as they calculated normality including data of all groups rather than calculating the normality group by group. The final output of statistically significant differences changes slightly in some instances.

We complied with the suggestion of the reviewer and calculated normality (Shapiro-Wilk test) group by group (Figure 7—source data 1, Figure 9—source data 1). We agree with the reviewer that the data when calculated group by group passes the normality test and accordingly, for comparison between the groups, one–way ANOVA, Tukey’s multiple comparison test has been employed in the revised manuscript.

a) For example, when analyzing the AUC of the GTT they come to the conclusion that "the glucose tolerance of the mice fed on HFD become comparable to the control group fed on the chow diet thereby demonstrating efficient the glucose-lowering ability of MS-275 and liraglutide combined therapy". Actually, the Shapiro-Wilk test for normality of distribution, considering one outlier value in the control HFD group (38130) shows normal distribution of values. It follows that the only statistically significant differences are "Ctrl vs. MS", "Ctrl vs. L+M" and "Ctrl vs. Chow", while all other comparisons were not statistically significant. Since all the treatments (i.e., both monotherapies and the combined therapy) were not statistically significant different vs. chow diet and because the treatment with MS-275 alone was not significantly different vs. the combined therapy, the conclusion should be that MS-275 monotherapy and combined therapy are both efficient.

Fasting hyperglycemia repeat dosing (Figure 7B): Shapiro –Wilk test shows normal distribution (Figure 7—source data 1); accordingly, the comparison between the groups was carried out by one–way ANOVA, Tukey’s multiple comparison test. We stated that the data thus demonstrates efficient blood glucose reduction both by monotherapies as well as by the combined therapy upon repeat dosing.

IPGTT (AUC) (Figure 7D): Shapiro –Wilk test shows normal distribution (Figure 7— source data 1), (considering one outlier value in the control HFD group (38130) as suggested by the reviewer). Accordingly, the comparison between the groups was carried out by one–way ANOVA, Tukey’s multiple comparison test. We stated that Glucose tolerance was also improved with MS-275 monotherapy as compared to vehicle (reduction from AUC 29322 ± 1764 arbitrary units to AUC 17733 ± 1108 arbitrary units; p<0.01, Tukey’s multiple comparison test) (Figure 7*D*) demonstrating that both MS-275 monotherapy as well as liraglutide and MS-275 combined therapy are efficient in improving glycemic control in DIO rodent model.

b) Likewise, for the Shapiro-Wilk test for normality of distribution in the comparisons for epididymal, mesenteric and retroperitoneal fat. When calculating the normality for distribution, the authors pooled all the data while the normality should be calculated for each experimental group. This way, the normality test was passed with all groups and all three fat depots. For epididymal and mesenteric fat, both monotherapies and combined therapy reduce fat mass vs. vehicle controls, and there is no difference between combined therapy and both monotherapies. For retroperitoneal fat, only combined therapy reduces fat mass, MS-275 and combined therapies are significantly different vs. liraglutide, while combined therapy does not differ from MS-275. In sum, in no instances the combined therapy seems to be superior to MS-275 monotherapy in reducing any of the fat depots.

Shapiro –Wilk test shows the normal distribution for all groups in the case of epididymal, mesenteric, and retroperitoneal WAT (Figure 9—source data 1) and accordingly comparison between the groups was carried out by one–way ANOVA, Tukey’s multiple comparison test. We mentioned that both monotherapies and the combined therapy reduce epididymal and mesenteric WAT mass as compared to vehicle control. We also stated that the results taken together revealed the comparable efficacy of MS-275 monotherapy and liraglutide and MS-275 combined therapy in decreasing visceral obesity in the DIO rodent model.

c) The asterisks for statistical significances should be modified accordingly in all figures.

The asterisks for statistical significance has been modified accordingly in all figures.

d) In addition, I recommend running the normality tests considering each group separately and not pooling data of all groups together also for the comparisons of gene expression."

As suggested, we carried out normality tests considering each group separately for the comparisons of gene expression (Figure 9—source data 1).

i) PPAR α: Values for the vehicle, Liraglutide, and Liraglutide +MS-275 normally distributed, Values in the MS-275 group is also normally distributed considering one outlier (MS-3:8.0657). Since vehicle, Liraglutide, and combined therapy group are normally distributed and deviation from normality in MS-275 group is not extreme (Pearson 1931 and Blanca 2017) we carried out one–way ANOVA, Tukey’s multiple comparison test for comparison between the groups. We stated that the significant increase in PPAR α gene expression vis-à-vis vehicle control is comparable between the groups on MS-275 monotherapy and MS-275 and liraglutide combined therapy, liraglutide monotherapy was ineffective in altering PPAR α gene expression at the indicated dose.

ii) CIDEA: Shapiro –Wilk test shows the normal distribution for all groups and hence we carried out one–way ANOVA, Tukey’s multiple comparison test for comparison between the groups. We mentioned that the increase in CIDEA (Cell Death-Inducing DFFA-like effector A) gene expression is comparable among mice on liraglutide or MS -275 monotherapy as well as the group receiving combined therapy.

iii) PGC1 α: Vehicle, Liraglutide, and MS-275 are normally distributed; in the case of Lira+MS275, the computed p-value is 0.034. Since deviation from normality is not extreme (Pearson 1931 and Blanca 2017) we carried out one–way ANOVA, Tukey’s multiple comparison test for comparison between the groups. Comparison between the groups was marked by asterisks in the figures for the significant difference and with letters ns where the difference is non-significant

iv) UCP1: In the case of vehicle control and MS-275, the data is normally distributed; since the deviation from normality is not extreme (Pearson 1931 and Blanca 2017) in the case of Liraglutide and Liraglutide + MS-275, we carried out one–way ANOVA, Tukey’s multiple comparison test for comparison between the groups. Comparison between the groups was marked by ‘asterisks’ in the figures for the significant difference and with letters ‘ns’ where the difference is non-significant.

References:

1. M. L. Hribal et al., Insulin-like growth factor-I, inflammatory proteins, and fibrosis in subjects with nonalcoholic fatty liver disease. J Clin Endocrinol Metab 98, E304-308 (2013).

2. A. Adamek, A. Kasprzak, Insulin-Like Growth Factor (IGF) System in Liver Diseases. Int J Mol Sci 19 (2018).

3. Y. Luo et al., Liraglutide Improves Non-Alcoholic Fatty Liver Disease In Diabetic Mice By Modulating Inflammatory Signaling Pathways. Drug Des Devel Ther 13, 4065-4074 (2019).

4. H. Gao et al., The Glucagon-Like Peptide-1 Analogue Liraglutide Inhibits Oxidative Stress and Inflammatory Response in the Liver of Rats with Diet-Induced Non-alcoholic Fatty Liver Disease. Biol Pharm Bull 38, 694-702 (2015).

5. K. Mahdaviani, I. Benador, O. Shirihai, Assessment of Brown Adipocyte Thermogenic Function by High-throughput Respirometry. Bio Protoc 5 (2015).

6. A. Galmozzi et al., Inhibition of class I histone deacetylases unveils a mitochondrial signature and enhances oxidative metabolism in skeletal muscle and adipose tissue. Diabetes 62, 732742 (2013).

7. E. Zhu et al., Liraglutide suppresses obesity and induces brown fat-like phenotype via Soluble Guanylyl Cyclase mediated pathway in vivo and in vitro. Oncotarget 7, 81077-81089 (2016).

8. S. B. Girada et al., Galphas regulates Glucagon-Like Peptide 1 Receptor-mediated cyclic AMP generation at Rab5 endosomal compartment. Mol Metab 6, 1173-1185 (2017).

9. J. T. Patterson, P. Li, J. W. Day, V. M. Gelfanov, R. D. Dimarchi, A hydrophobic site on the GLP1 receptor extracellular domain orients the peptide ligand for signal transduction. Mol Metab 2, 86-91 (2013).

10. D. Calebiro et al., Persistent cAMP-signals triggered by internalized G-protein-coupled receptors. PLoS Biol 7, e1000172 (2009).

11. S. Ferrandon et al., Sustained cyclic AMP production by parathyroid hormone receptor endocytosis. Nat Chem Biol 5, 734-742 (2009).

12. R. Irannejad et al., Conformational biosensors reveal GPCR signalling from endosomes. Nature 495, 534-538 (2013).

13. A. R. Thomsen et al., GPCR-G Protein-beta-Arrestin Super-Complex Mediates Sustained G Protein Signaling. Cell 166, 907-919 (2016).

14. R. S. Kuna et al., Glucagon-like peptide-1 receptor-mediated endosomal cAMP generation promotes glucose-stimulated insulin secretion in pancreatic beta-cells. Am J Physiol Endocrinol Metab 305, E161-170 (2013).

15. S. Ismail et al., Internalized Receptor for Glucose-dependent Insulinotropic Peptide stimulates adenylyl cyclase on early endosomes. Biochem Pharmacol 120, 33-45 (2016).

16. S. Asalla et al., Restoring Mitochondrial Function: A Small Molecule-mediated Approach to Enhance Glucose Stimulated Insulin Secretion in Cholesterol Accumulated Pancreatic beta cells. Sci Rep 6, 27513 (2016).

17. T. N. Feinstein et al., Retromer terminates the generation of cAMP by internalized PTH receptors. Nat Chem Biol 7, 278-284 (2011).

18. A. Ferrari et al., Attenuation of diet-induced obesity and induction of white fat browning with a chemical inhibitor of histone deacetylases. Int J Obes (Lond) 41, 289-298 (2017).

19. S. Kooijman et al., Central GLP-1 receptor signalling accelerates plasma clearance of triacylglycerol and glucose by activating brown adipose tissue in mice. Diabetologia 58, 26372646 (2015).

20. H. J. van Eyk et al., Liraglutide decreases energy expenditure and does not affect the fat fraction of supraclavicular brown adipose tissue in patients with type 2 diabetes. Nutr Metab Cardiovasc Dis 30, 616-624 (2020).

21. M. Horowitz et al., Effect of the once-daily human GLP-1 analogue liraglutide on appetite, energy intake, energy expenditure and gastric emptying in type 2 diabetes. Diabetes Res Clin Pract 97, 258-266 (2012).

22. E. T. Chouchani, L. Kazak, B. M. Spiegelman, Mitochondrial reactive oxygen species and adipose tissue thermogenesis: Bridging physiology and mechanisms. J Biol Chem 292, 1681016816 (2017).

23. T. A. Wadden et al., Weight maintenance and additional weight loss with liraglutide after lowcalorie-diet-induced weight loss: the SCALE Maintenance randomized study. Int J Obes (Lond) 37, 1443-1451 (2013).

24. M. Li, S. Yang, P. Bjorntorp, Metabolism of different adipose tissues in vivo in the rat. Obes Res 1, 459-468 (1993).

25. V. Plaisance et al., The class I histone deacetylase inhibitor MS-275 prevents pancreatic beta cell death induced by palmitate. J Diabetes Res 2014, 195739 (2014).

26. E. T. Chouchani et al., Mitochondrial ROS regulate thermogenic energy expenditure and sulfenylation of UCP1. Nature 532, 112-116 (2016).

27. L. P. Roma, J. C. Jonas, Nutrient Metabolism, Subcellular Redox State, and Oxidative Stress in Pancreatic Islets and beta-Cells. J Mol Biol 432, 1461-1493 (2020).
